# Trypanocidal Activity of Flavanone Derivatives

**DOI:** 10.3390/molecules25020397

**Published:** 2020-01-17

**Authors:** Gabriela Maciel Diogo, Josimara Souza Andrade, Policarpo Ademar Sales Junior, Silvane Maria Fonseca Murta, Viviane Martins Rebello Dos Santos, Jason Guy Taylor

**Affiliations:** 1 Chemistry Department, ICEB, Federal University of Ouro Preto, Campus Universitário Morro do Cruzeiro, Ouro Preto 35400-000, MG, Brazil; gabriela.diogo1@gmail.br (G.M.D.); josimara.souza28@hotmail.com (J.S.A.); vivianesantos@ufop.edu.br (V.M.R.D.S.); 2 René Rachou Institute, FIOCRUZ, Belo Horizonte 30190-002, MG, Brazil; policarpoasjunior@cpqrr.fiocruz.br (P.A.S.J.); silvane@cpqrr.fiocruz.br (S.M.F.M.)

**Keywords:** Chagas disease, flavanones, flavonoids, *Trypanosoma cruzi*, chromanones, in vitro, nitroreductase

## Abstract

Chagas disease, also known as American trypanosomiasis, is classified as a neglected disease by the World Health Organization. For clinical treatment, only two drugs have been on the market, Benznidazole and Nifurtimox, both of which are recommended for use in the acute phase but present low cure rates in the chronic phase. Furthermore, strong side effects may result in discontinuation of this treatment. Faced with this situation, we report the synthesis and trypanocidal activity of 3-benzoyl-flavanones. Novel 3-benzoyl-flavanone derivatives were prepared in satisfactory yields in the 3-step synthetic procedure. According to recommended guidelines, the whole cell-based screening methodology was utilized that allowed for the simultaneous use of both parasite forms responsible for human infection. The majority of the tested compounds displayed promising anti-*Trypanosoma cruzi* activity and the most potent flavanone bearing a nitrofuran moiety was more potent than the reference drug, Benznidazole.

## 1. Introduction

The Brazilian researcher Carlos Ribeiro Justiniano das Chagas discovered Chagas disease (also known as American trypanosomiasis) in 1909 and revealed that the etiologic agent to be the parasite *Trypanosoma cruzi (T. cruzi)* [1]. Chagas disease is classified by the World Health Organization (WHO) as a neglected disease along with dengue, rabies, African trypanosomiasis, among others [2,3,4]. In a recent survey conducted between 2008 and 2017, over 90% of reported cases of Chagas disease in the world were confirmed in South America [5]. A common form of transmission occurs through contact of the mucosa of the open wound with contaminated feces of the triatomine bug [5,6,7]. However, the transmission is not only caused by direct contact with the parasite, but it can also be caused by indirect contact occurring by ingestion of contaminated food containing feces of the triatomine bug. Indeed, studies suggest that the most probable forms of transmission were oral transmission (72%), the minority being caused by vector transmission (9%) and the remaining 19% were unidentified forms of transmission [5].

In the acute phase of Chagas disease, symptoms like fever, discomfort, and facial edema are common but may disappear spontaneously between four to six weeks post-infection. Treatment at this stage is still possible, but commonly these symptoms go unnoticed and the disease transitions many years later into the chronic phase when the heart is already severely compromised (chagasic cardiomyopathy) and available drugs are no longer effective. Furthermore, the parasite and its major vectors can also cause lesions in the liver and in the nervous and lymphatic systems [6].

Treatment of Chagas disease is restricted to Benznidazole and Nifurtimox, both of which were introduced in the market in the 1960s and 1970s. Both of these drugs have a few drawbacks such as: (i) moderate efficacy in the acute phase and low efficacy in the chronic phase of the disease; (ii) side effects such as digestive disorders, fevers, muscle pain, loss of appetite, amnesia, hematological disorders (Nifurtimox), and hypertensive dermatitis (Benznidazole); (iii) require follow-up under medical supervision for longer periods of time; (iv) absence of pediatric doses; (v) not being indicated for pregnant patients. Nifurtimox was withdrawn from the Brazilian market due to its high toxicity, making Benznidazole the only available treatment [6,7].

**Figure 1:**
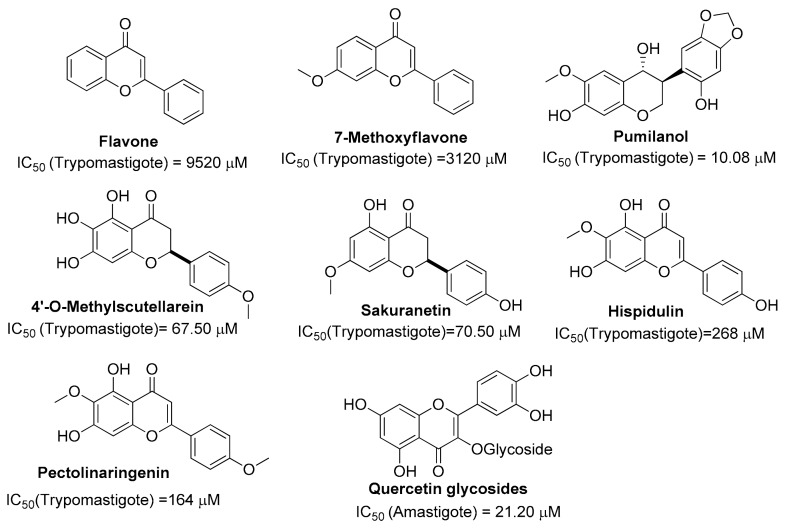
Structure of flavonoids with anti-*Trypanosoma cruzi* activity.

Many plant extracts have rendered flavonoids with anti-*trypanosoma cruzi* activity (Figure 1). Ambrozin et al. isolated and evaluated the activity of two flavonoids from the leaf extract belonging to the Rutaceae (*Conchocarpus heterophyllus*) family against the trypomastigote form of *T. cruzi* Ganapaty et al. isolated from the roots of *Tephrosia pumila* (Fabaceae) Pumilanol, which displayed promising anti *T. cruzi* activity and low cytotoxicity when compared to the reference compound [8]. Grecco et al. isolated four flavonoids (4′-O-Methylscutellarein, Sakuranetin, Hispidulin, and Pectolinaringenin) from the *Baccharis retusa* (Asteraceae) plant and found two of these flavonoid compounds to be more potent than the reference drug [9,10,11,12]. Marin et al. isolated quercetin glycosides from *Delphinium gracile* (Ranunculaceae), which were also evaluated against the amastigote form of *Trypanosoma cruzi.* Although this compound displayed high trypanocidal activity, it was more toxic than the reference drug compound [13].

The aforementioned studies have shown that flavonoids exhibit promising trypanocidal activities. Thus, motivated by the negative economic and social impact caused by Chagas disease, lack of alternative chemotherapies, especially in the chronic phase and the existence of naturally resistant *T. cruzi* strains, this work describes the synthesis and trypanocidal activity of 3-benzoyl-flavanones.

## 2. Results

### 2.1. Synthesis of 3-Benzoyl Flavanones

Esterification of 2-hydroxyacetophenones with substituted benzoyl chlorides afforded initially the corresponding esters, which were immediately subjected to a Baker–Venkataraman rearrangement in the presence of KOH to afford the desired 1,3-diketones **1**. All of the ester and 1,3-diketone intermediates are known compounds and were, therefore, confirmed by comparison of their NMR spectral data and melting points with literature values (see experimental section).

**Scheme 1:**
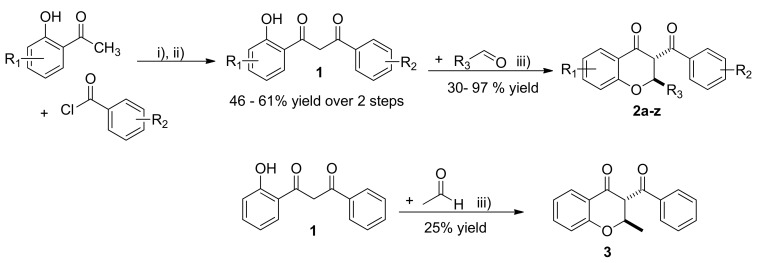
Synthetic route for the preparation of flavanone derivatives: (**i**) pyridine, rt, 1 h; (**ii**) pyridine, KOH, 50 °C, 1 h; (**iii**) morpholine (10 mol%), ethanol, reflux, 1 h.

**Figure 2:**
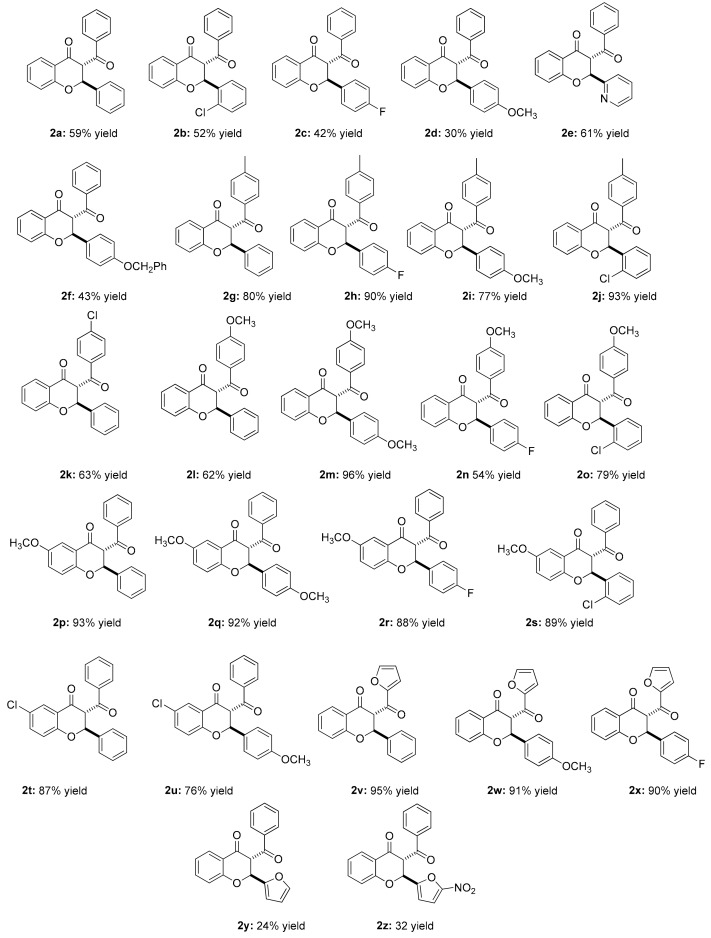
Synthesized 3-benzoyl-flavanones.

Subsequently, 3-benzoyl-flavanone derivatives **2a**–**z** were synthesized by a domino aldol condensation intramolecular *oxi*-Michael reaction between β-diketones and aldehydes in the presence of morpholine (Scheme 1). In total, 28 3-benzoyl-flavanone derivatives were obtained in satisfactory yields ranging from 30 to 97%, and 16 of these are unpublished compounds (Figure 2). In order to determine what, if any, the importance of an aryl group at position 2 would play in the overall trypanocidal activity of the target compounds, 3-benzoyl-2-methylchroman-4-one **3** was prepared by reacting **1** with acetaldehyde (Scheme 1).

**Scheme 2:**
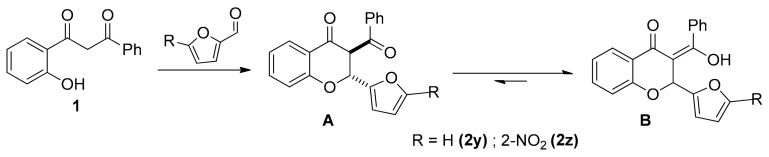
Equilibrium for keto–enol tautomerism.

All flavanone derivatives were characterized by infrared (IR) spectroscopy, ^1^H and ^13^C Nuclear Magnetic Resonance spectroscopy, and high-resolution mass spectrometry. In the ^1^H NMR spectrum, 3-benzoyl-flavanones characteristically display two doublet resonances corresponding to the methine hydrogens present at the dihydropyranone ring (~5.17 ppm and 6.02 ppm, respectively). Rao et al. described the synthesis of both *cis* and *trans* 7-methoxy-2,3-dimethylchroman-4-one and showed that the coupling constant for the *cis* compound to be approximately 3.4 Hz and the *trans* being larger around 11.5 Hz [14]. The *trans* configuration was, therefore, attributed to compounds **2a**–**z** based on comparison of their coupling constants with similar compounds in the literature given that the coupling constant for 3-benzoyl-flavanones was 12 Hz. Notably, the ^1^H NMR spectra for compounds **2y** and **2z** were in solution observed exclusively in their keto-enol tautomeric form (Scheme 2). The presence of a furan ring possibly favors form **B** by stabilization of an intramolecular hydrogen bond between the furan oxygen and enol hydroxyl group (Scheme 2). The enol form for similar 3-benzoylchromanones has also been observed in CDCl_3_ by other groups [15,16].

**Scheme 3:**
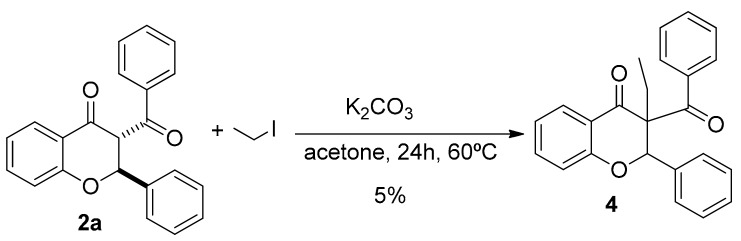
Alkylation of compound **2a**.

It is well established that both Benznidazole and Nifurtimox are prodrugs that require the enzyme nitroreductase for their trypanocidal effects. For this reason, compound **2z** was synthesized bearing a nitrofuran for the purpose of evaluating possible improvement in activity via a known biological mechanism of action. In order to maintain equivalency with Nifurtimox, the nitro group was introduced to position 5 of the furan ring. Furthermore, compound **2a** was alkylated with iodoethane and converted to compound **4** to afford a flavanone with greater lipophilic character (Scheme 3).

**Scheme 4:**
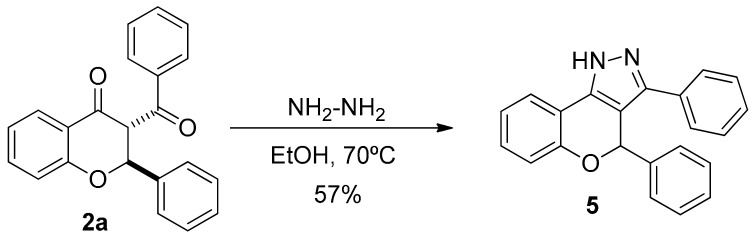
Synthesis of compound **5**.

Given that tricyclic compounds have demonstrated excellent trypanocidal activities [17,18], the chromenopyrazol **5** was obtained by condensation of hydrazine with **2a** (Scheme 4) and evaluated for anti-*T. cruzi* activity.

Thus, with the target compounds in hand, in vitro bioassays using trypomastigote and amastigote forms of Y-strain *T. cruzi* were carried out.

### 2.2. Evaluation of In Vitro Anti-T. cruzi Activity

Flavanone derivatives were evaluated for anti-*T. cruzi* activity against the intracellular forms of the parasite. Although the use of epimastigotes present in the midgut vector may be utilized for initial screening [19,20], we have instead opted for the simultaneous use of trypomastigote and intracellular amastigotes forms that are present during both acute and chronic phases of the disease [21,22]. This approach is in accordance with the Drugs for Neglected Diseases Initiative guidelines (DNDi) [23]. Moreover, it also has the advantage of evaluating bioactive compounds in infected cells while at the same time monitoring their effects on both the relevant forms of the parasite in the same system. Benznidazole was used as a positive control against *T. cruzi* and cytotoxicity was determined in mammalian L929 cells (Table 1). The bioavailability profile of the flavanones was evaluated by applying the Lipinski rule and data related to the risk of toxicity (mutagenicity, tumorigenicity, irritability, and effects on the reproductive system) carried out through online programs “Molinspiration” and “OSIRIS property explorer” (Table 1). The majority of the tested compounds displayed in varying degrees some anti-*T. cruzi* activity and only 6 of the 29 compounds tested were found to be inactive. Initially, compound **2a** was evaluated for trypanocidal activity and this result used for comparison in order to assess structure–activity relationships. The 50% inhibitory concentration (IC_50_) for **2a** (8.8 µM) was only slightly less potent than Benznidazole. Nevertheless, this preliminary result motivated us to investigate other analogs bearing substituents in order to improve potency and selectivity. The importance of the aryl group at the 2-position was demonstrated by comparison of trypanocidal activity of compounds **2a** and **3**. Indeed, the absence of the benzene ring resulted in a completely inactive compound and, for this reason, the aryl group was maintained or substituted for other heteroaromatic moieties. Compounds **2a**–**f** all possess a benzoyl group at the 3-position but bear different substituents at the aryl group located at the 2-position. Among these, only the introduction of an anisole moiety (**2d**) significantly improved anti-*T. cruzi* activity, resulting in a doubling in potency when compared to **2a**. Moreover, the selectivity of **2d** was almost 4 times greater than **2a**. The importance of methoxy substituents for trypanocidal activity has been noted in other studies [17,24]. In the case of tricyclic coumarins, a 6–7-fold improvement in trypanocidal activity was observed with the introduction of methoxy substituents. These compounds resembled more closely the chemical structure of the trypanocidal natural product (brevifolin carboxylate) that these structures were based on [17]. Some enhancement in the trypanocidal activity of hybrid coumarin-chalcone compounds was also noted when the methoxy groups were introduced into either the 2 or 5 positions of the benzene ring [24]. The inclusion of a pyridine moiety in compound **2e** was supported by previous reports describing the anti-*T. cruzi* activity of 2-pyridyl derivatives and their capacity to inhibit cruzain catalytic activity [25,26]. However, on this occasion, compound **2e** exhibited unremarkable anti-*T. cruzi* activity and was also not very selective. No improvements in anti-*T. cruzi* activity were also observed for flavanones substituted at the benzoyl moiety (**2g**–**2o** and **2v**–**2x**). The introduction of a methoxy or a chloro substituent at the 6-position of the chromanone nucleus (**2p**–**2u**) also did not provide more active compounds than **2a**, although some improvements in selectivity were observed. Given that the activation of nitroheterocyclic drugs by *T. cruzi* has been shown to be associated with the formation of reactive radical species responsible for the death of the parasite, it was no surprise that the most potent flavanone **2z** (IC_50_ = 2.6 μM) was superior to the reference compound (3.8 μM). These results are in agreement with studies showing that in general nitrofuryl derivatives are highly potent anti-*T. cruzi* compounds that will often display comparable trypanocidal activities to Nifurtimox [27,28]. The physicochemical drug descriptors of the molecular properties for the synthesized compounds were calculated by Molinspiration software (Table 1). The majority of the tested compounds satisfy the Lipinski rule with no violations and therefore displaying potentially good bioavailability. There were no linear correlations observed between molecular hydrophobicity and bioactivity. All Log *p*-values for the bioactive flavanones were approximately 3–4 times greater than Benznidazole and all values were less than 5, which satisfies Lipinski’s rule of five and suggests potentially good permeability across cell membranes. Interestingly, compound **2z** presented the highest total polar surface area (TPSA) value, which is below the limit of 140 A^2^ (Lipinski’s rule) and notably was also the most closely related TPSA value to reference drug Benznidazole. The risk of theoretical toxicity revealed that only **2z** had a low risk of the mutagenic effect but unlike Benznidazole, it was not high risk for reproductive effects.

## 3. Materials and Methods

All commercial reagents were used as received. Anhydrous solvents were purchased from Sigma-Aldrich. Flash column chromatography was performed using silica gel 200–400 Mesh. TLC analyses were performed using silica gel plates, using ultraviolet light (254 nm), phosphomolybdic acid, or vanillin solution for visualization. Melting points are uncorrected and were recorded on a Buchi B-540 apparatus. For NMR data, the chemical shifts are reported in *δ* (ppm) referenced to residual solvent protons and ^13^C signals in deuterated chloroform. Coupling constants (*J*) are expressed in Hertz (Hz). Infrared spectra were obtained on a Thermo Scientific Nicolet 380 FT-IR apparatus (600–4000 cm^−1^, Nicolet Instrument Corp., Madison, WI, USA) using attenuated total reflection (ATR). Mass spectra were obtained by GC-MS, Shimadzu QP-2010 Plus model (Shimadzu, Kyoto, Japan) and High-Resolution Mass Spectra were obtained on a Shimadzu HPLC-ESI-IT-TOF. SMILES notations of the flavanone derivatives were inputted into an online software and subjected to molecular properties prediction by Molinspiration software (software version v2015.01). ^1^H and ^13^C NMR spectra of these compounds are available in the supplementary materials.

### 3.1. Typical Procedure for the Synthesis of Diketones ***1***

In a round bottom flask (50.0 mL) equipped with stir bar, substituted 2-hydroxyacetophenone (5 mmol), pyridine (15.0 mL), and substituted benzoyl chloride (2.5 mmol) were added at 0 °C. The reaction was allowed to warm to room temperature and left to stir for 1 h. Upon completion, the reaction was quenched with 3.0 M HCl solution (25 mL) at 0 °C and the precipitate that formed was filtered and recrystallized from hot methanol to afford esters as white solids. Next, the esters (2 mmol) were reacted with pyridine (20.0 mL) and KOH (3 mmol) in a round bottom flask (50.0 mL) equipped with stir bar at 50 °C for 30 min. At the end of the reaction, the mixture was poured into a flask containing an aqueous solution of 10% *v*/*v* acetic acid (20.0 mL). The yellow precipitate that formed was filtered and recrystallized from hot ethanol. All of the ester and 1,3-diketone intermediates are known compounds and were, therefore, confirmed by comparison of their NMR spectral data and melting points with literature values.2-acetylphenyl benzoate (**1a**) [29], 1-[2-(4-chlorobenzoyloxy)-phenyl]-ethanone (**1b**) [30], 2-acetylphenyl-4-methylbenzoate (**1c**) [30], 2′-(4-methoxybenzoyloxy)acetophenone (**1d**) [31], benzoic acid 2-acetyl-5-methoxyphenyl ester (**1e**) [30], 1-[2-(4-chlorobenzoyloxy)-phenyl]-ethanone (**1f**) [31], 2-acetylphenyl furan-2-carboxylate (**1g**) [32]; 1-(2-hydroxyphenyl)-3-phenyl-1,3-propanedione (**1h**) [33], 1-(4-chloro-phenyl)-3-(2-hydroxy-phenyl)-propane-1,3-dione (**1i**) [34], 1-(2-hydroxyphenyl)-3-(4-methylphenyl)propane-1,3-dione (**1j**) [35], 1-(2-hydroxyphenyl)-3-(4-methoxy-phenyl)-propane-1,3-dione (**1k**) [35], 1-(2-hydroxy-5-methoxyphenyl)-3-phenylpropane-1,3-dione (**1l**) [36], 1-(4-chloro-phenyl)-3-(2-hydroxy-phenyl)-propane-1,3-dione (**1m**) [34], 1-(2-hydroxyphenyl)-3-(furan-2-yl)propane-1,3-dione (**1n**) [37].

### 3.2. Typical Procedure for the Synthesis of Flavanones ***2a**–**z*** and ***3***

In a round bottom flask (50 mL), β-diketone (1 mmol), substituted benzaldehydes (1.2 mmol), morpholine (10 mol%), and ethanol (10 mL) were added. The mixture was allowed to stir for approximately 3 h at 70 °C. Upon completion, the reaction was cooled in an ice bath until a precipitate was formed. The product was filtered and washed with cold ethanol and finally, recrystallized with 70% aqueous ethanol.

Synthesis of trans-3-benzoyl-2-phenylchroman-4-one (**2a**): Product obtained as a white solid in 59% [38]. m.p.: 142–145 °C. R_f_: 0.6 (ethyl acetate/hexane 3:7). IR (cm^−1^): 3038, 2900, 1700, 1668, 1606, 1578, 1460, 1447, 1231, 1031. ^1^H-NMR (400 MHz, CDCl_3_): *δ* 5.17 (d, *J =* 12.0 Hz, 1 H), 6.02 (d, *J* = 12.0 Hz, 1 H), 7.09−7.13 (m, 2 H), 7.31–7.37 (m, 3 H), 7.40−7.44 (m, 2 H), 7.50−7.60 (m, 4 H), 7.79−7.81 (m, 2 H), 7.95−7.97 (m, 1 H). ^13^C-NMR (100 MHz, CDCl_3_): *δ* 59.7, 81.9, 118.2, 120.5, 121.9, 127.4, 128.6, 128.6, 128.8, 129.1, 133.6, 136.7, 137.1, 137.7, 161.3, 189.9, 196.1. HRMS (ESI-TOF) *m*/*z* [M − H] Calculated for C_22_H_15_O_3_: 327.1027. Found: 327.1040.

Synthesis of trans-3-benzoyl-2-(2-chlorophenyl)chroman-4-one (**2b**): Product obtained as a white solid in 52% [39]. m.p.: 157−160 °C. R_f_: 0.6 (ethyl acetate/hexane 3:7). IR (cm^−1^): 3054, 2925, 1689, 1658, 1602, 1583, 1466, 1447, 1231, 1035, 755; ^1^H-NMR (400 MHz, CDCl_3_): *δ* 5.45 (d, *J* = 12.0 Hz, 1 H), 6.44 (d, *J* = 12.0 Hz, 1 H), 7.10−7.14 (m, 2 H), 7.22−7.26 (m, 2 H), 7.41−7.48 (m, 4 H), 7.57−7.61 (m, 2 H), 7.90−7.96 (m, 3 H). ^13^C-NMR (100 MHz, CDCl_3_): *δ* 57.7, 78.4, 118.1, 120.4, 122.0, 127.2, 127.5, 128.7, 128.8, 130.4, 130.6, 133.7, 134.1, 134.2, 136.9, 137.2, 161.2, 189.3, 195.1. HRMS (ESI-TOF) *m*/*z* [M + H]^+^ Calculated for C_22_H_16_ClO_3_: 363.0782. Found: 363.0793.

Synthesis of trans-3-benzoyl-2-(4-fluorophenyl)chroman-4-one (**2c**): Product obtained as a white solid in 42%. m.p.: 137–140 °C. R_f_: 0.6 (ethyl acetate/hexane 3:7). IR (cm^−1^): 3077, 2921, 1687, 1658, 1602, 1578, 1508, 1469, 1308, 1215, 1035. ^1^H-NMR (400 MHz, CDCl_3_): *δ* 5.12 (d, *J* = 12.0 Hz, 1 H), 6.00 (d, *J* = 12.0 Hz, 1 H), 7.01–7.06 (m, 2 H), 7.11–7.14 (m, 2 H); 7.42–7.61 (m, 6 H); 7.79–7.82 (m, 2 H), 7.96 (d, *J* = 8.0 Hz, *J* = 1.6 Hz, 1 H). ^13^C-NMR (100 MHz, CDCl_3_): *δ* 59.8, 81.2, 115.6 and 115.7 (d, *J* = 22 Hz), 118.2, 120.5, 122.1, 127.5, 128.6, 128.7, 129.3, and 129.4 (d, *J* = 8 Hz), 133.1 and 133.2 (d, *J* = 3 Hz), 133.7, 136.8, 137.58, 161.1, 161.1 and 164.1 (d, *J* = 246 Hz;), 189.7, 196.0. HRMS (ESI-TOF) *m*/*z* [M − H]^−^ Calculated for C_22_H_14_FO_3_: 345.0932. Found: 345.0930.

Synthesis of trans-3-benzoyl-2-(4-methoxyphenyl)chroman-4-one (**2d**): Product obtained as a white solid in 30%. m.p.: 125−126 °C. R_f_: 0.6 (ethyl acetate/hexane 3:7). IR (cm^−1^): 3032, 2830, 1693, 1673, 1605, 1587, 1515, 1469, 1255, 1047. ^1^H-NMR (400 MHz, CDCl_3_): *δ* 3.78 (s, 3 H), 5.16 (d, *J* = 12.0 Hz, 1 H), 5.96 (d, *J* = 12.0 Hz, 1 H), 6.87 (d, *J* = 8.0 Hz, 2 H), 7.08−7.12 (m, 2 H), 7.42−7.45 (m, 4 H), 7.54−7.60 (m, 2 H), 7.82 (d, *J* = 8.0 Hz, 2 H), 7.95 (d, *J* = 8.0 Hz, 1 H). ^13^C-NMR (100 MHz, CDCl_3_): *δ* 55.3, 59.6, 81.5, 114.1, 118.2 120.6, 121.8, 127.4, 128.6, 128.6, 128.8, 129.3, 133.55, 136.70, 137.7, 160.0, 161.3, 190.2, 196.2. HRMS (ESI-TOF) *m*/*z* [M + H]^+^ Calculated for C_23_H_19_O_4_: 359.1278. Found: 359.1296.

Synthesis of trans-3-benzoyl-2-(pyridin-2-yl)chroman-4-one (**2e**): Product obtained as a white solid in 61%. m.p.: 128−129 °C. R_f_: 0.6 (ethyl acetate/hexane 3:7). IR (cm^−1^): 3054, 2902, 1701, 1662, 1602, 1577, 1515, 1462, 1300, 1219, 1034. ^1^H-NMR (400 MHz, CDCl_3_): *δ* 5.71 (d, *J* = 12.0 Hz, 1 H), 6.17 (d, *J* = 12.0 Hz, 1 H), 7.07−7.12 (m, 2 H), 7.22−7.26 (m, 1 H), 7.46−7.61 (m, 5 H), 7.71−7.45 (m, 1 H), 7.91−7.94 (m, 1 H), 8.10 (d, *J* = 8.0 Hz, 2 H), 8.50 (d, *J* = 4.0 Hz, 1 H). ^13^C-NMR (100 MHz, CDCl_3_): *δ* 56.9, 81.5, 118.1, 122.0, 123.9, 123.9, 127.3, 127.8, 128.6, 129.0, 133.4, 136.6, 137.0, 137.6, 149.3, 155.2, 160.7, 189.9, 196.5. HRMS (ESI-TOF) *m*/*z* [M + H]^+^ Calculated for C_21_H_16_NO_3_: 330.1125. Found: 330.1123.

Synthesis of trans-3-benzoyl-2-(4-(benzyloxy)phenyl)chroman-4-one (**2f**): Product obtained as a white solid in 43%. m.p.: 128−129 °C. R_f_: 0.6 (ethyl acetate/hexane 3:7). IR (cm^−1^): 3066, 2904, 1692, 1663, 1602, 1577, 1515, 1465 1265, 1238, 1075. 1029. ^1^H-NMR (400 MHz, CDCl_3_): *δ* 5.03 (s, 2 H), 5.17 (d, *J* = 12.0 Hz, 1 H), 5.96 (d, *J* = 12.0 Hz, 1 H), 6.93 (d, *J* = 8.0 Hz, 2 H), 7.07−7.12 (m, 2 H), 7.36−7.46 (m, 9 H), 7.54−7.60 (m, 2 H), 7.81−7.84 (m, 2 H), 7.95 (dd, *J* = 8.0 Hz, *J* = 1.6 Hz, 1 H). ^13^C-NMR (100 MHz, CDCl_3_): *δ* 59.5, 70.0, 81.6, 115.0, 118.2, 120.6, 121.9, 127.4, 128.0, 128.6, 128.8, 129.6, 133.6, 136.8, 137.7, 159.2, 161.3, 190.2, 196.2. HRMS (ESI-TOF) *m*/*z* [M + H]^+^ Calculated for C_29_H_23_O_4_: 435.1591. Found: 435.1584.

Synthesis of trans-3-(4-methylbenzoyl)-2-phenylchroman-4-one (**2g**): Product obtained as a white solid in 80% [39]. m.p.: 167 °C. R_f_: 0.6 (ethyl acetate/hexane 3:7). IR (cm^−1^): 3041, 2917, 1695, 1672, 1605,1579, 1476, 1448, 1340, 1237, 1026. ^1^H-NMR (400 MHz, CDCl_3_): *δ* 2.39 (s, 3 H), 5.14 (d, *J* = 12.0 Hz, 1 H), 6.02 (d, *J* = 12.0 Hz, 1 H), 7.09−7.12 (m, 2 H), 7.21 (d, *J* = 8.0 Hz, 2 H), 7.31−7.37 (m, 3 H), 7.50−7.60 (m, 3 H), 7.72 (d, *J* = 8.0 Hz, 2 H), 7.96 (dd, *J* =8.0 Hz, *J* = 2.0 Hz 1 H). ^13^C-NMR (100 MHz, CDCl_3_): 21.7, 59.5, 81.9, 118.2, 120.6, 121.9, 127.4, 128.7, 129.1, 129.3, 135.2, 136.7, 137.2, 144.6, 161.2, 189.9, 195.5. HRMS (ESI-TOF) *m*/*z* [M + H]^+^ Calculated for C_23_H_19_O_3_: 343.1329. Found: 343.1338.

Synthesis of trans-2-(4-fluorophenyl)-3-(4-methylbenzoyl)chroman-4-one (**2h**): Product obtained as a white solid in 93%. m.p.: 133–134 °C. R_f_: 0.6 (ethyl acetate/hexane 3:7). IR (cm^−1^): 3046, 2917, 1699, 1664, 1604, 1580, 1514, 1462, 1345, 1302, 1226, 1030. ^1^H-NMR (400 MHz, CDCl_3_): *δ* 2.40 (s, 3 H), 5.09 (d, *J* = 12.0 Hz, 1 H), 6.00 (d, *J* = 12.0 Hz, 1 H), 7.01–7.13 (m, 4 H), 7.23 (d, *J* = 8.0 Hz, 2 H), 7.48–7.52 (m, 2 H), 7.56–7.60 (m, 1 H), 7.72 (d, *J* = 8.0 Hz, 2 H), 7.96 (dd, *J* = 8.0 Hz, *J* = 2.0 Hz 1 H). ^13^C-NMR (100 MHz, CDCl_3_): *δ* 21.7, 59.6, 81.2, 115.6 and 115.8 (d, *J* = 22 Hz), 118.1, 120.6, 122.1, 127.5, 128.7, 129.2 and 129.3 (d, *J =* 8 Hz), 129.4, 133.2 and 133.2 (d, *J =* 3 Hz), 135.2, 136.7, 144.9, 161.1, 161.6 and 164.1 (d, *J =* 247 Hz), 189.7, 195.4. HRMS (ESI-TOF) *m*/*z* [M − H] Calculated for C_23_H_16_FO_3_: 359.1089. Found: 359.1075.

Synthesis of trans-2-(4-methoxyphenyl)-3-(4-methylbenzoyl)chroman-4-one (**2i**): Product obtained as a white solid in 77%, m.p.: 131–133 °C. R_f_: 0.6 (ethyl acetate/hexane 3:7). IR (cm^−1^): 3030, 2836, 1693, 1673, 1603, 1584, 1515, 1456, 1348, 1259, 1235, 1086, 1029. ^1^H-NMR (400 MHz, CDCl_3_): *δ* 2.40 (s, 3 H), 3.78 (s, 3 H), 5.12 (d, *J* = 12.0 Hz, 1 H), 5.95 (d, *J* = 12.0 Hz, 1 H), 6.86 (d, *J* = 8.0 Hz, 2 H), 7.07–7.11 (m, 2 H), 7.23 (d, *J* = 8.0 Hz, 2 H), 7.43 (d, *J* = 8.0 Hz, 2 H), 7.55–7.58 (m, 1 H), 7.74 (d, *J* = 8.0 Hz, 2 H), 7.94 (dd, *J* = 8.0 Hz, *J* = 2.0 Hz, 1 H). ^13^C-NMR (100 MHz, CDCl_3_): *δ* 21.7, 55.3, 59.4, 81.6, 114.1, 118.2, 120.6, 121.8, 127.4, 128.7, 128.8, 129.3, 129.4, 135.3, 136.6, 144.6, 159.9, 161.3, 190.2, 195.6. HRMS (ESI-TOF) *m*/*z* [M + H]^+^ Calculated for C_24_H_21_O_4_: 373.1434. Found: 373.1435.

Synthesis of trans-2-(2-chlorophenyl)-3-(4-methylbenzoyl)chroman-4-one (**2j**): Product obtained as a white solid in 97%. m.p.: 175–176 °C. R_f_: 0.6 (ethyl acetate/hexane 3:7). IR (cm^−1^): 3036, 2971, 1658, 1601, 1573, 1470, 1459, 1230, 1026, 755. ^1^H-NMR (400 MHz, CDCl_3_): *δ* 2.41 (s, 3 H), 5.42 (d, *J* = 12.0 Hz, 1 H), 6.43 (d, *J* = 12.0 Hz, 1 H), 7.09–7.13 (m, 2 H), 7.23–7.27 (m, 4 H), 7.41–7.47 (m, 2 H), 7.57–7.61 (m, 1 H), 7,87 (d, *J* = 8.0 Hz, 2 H), 7,94 (dd, *J* = 8.0 Hz, *J* = 2.0 Hz, 1 H). ^13^C-NMR (100 MHz, CDCl_3_): *δ* 21.7, 57.6, 78.4, 118.0, 120.4, 121.9, 127.2, 127.4, 128.8, 128.9, 129.4, 130.3, 130.6, 134.1, 134.3, 134.8, 136.8, 144.8, 161.2, 189.3, 194.4. HRMS (ESI-TOF) *m*/*z* [M − H] Calculated for C_23_H_16_ClO_3_: 375.0793. Found: 375.0786.

Synthesis of trans-3-(4-chlorobenzoyl)-2-phenylchroman-4-one (**2k**): Product obtained as a white solid in 63% [40]. m.p.: 166–167 °C. R_f_: 0.7 (ethyl acetate/hexane 3:7). IR (cm^−1^): 3054, 2904, 1686, 1658, 1602, 1580, 1500, 1462, 1272, 1040, 755. ^1^H-NMR (400 MHz, CDCl_3_): *δ* 5.07 (d, *J* = 12.0 Hz, 1 H), 5.97 (d, *J* = 12.0 Hz, 1 H), 7.07–7.11 (m, 2 H), 7.30–7.38 (m, 5 H), 7.47 (d, *J* = 8.0 Hz, 2 H), 7.54–7.59 (m, 1 H), 7.72 (d, *J* = 8.0 Hz, 2 H), 7,93 (d, *J* = 8.0 Hz, 1 H). ^13^C-NMR (100 MHz, CDCl_3_): *δ* 60.0, 82.0, 118.4, 120.7, 121.1, 122.2, 127.5, 127.6, 129.0, 129.2, 129.4, 130.2, 136.2, 137.1, 137.3, 161.5, 189.8, 195.1. HRMS (ESI-TOF) *m*/*z* [M + H]^+^ Calculated for C_22_H_16_ClO_3_: 363.0782. Found: 363.0790.

Synthesis of trans-3-(4-methoxybenzoyl)-2-phenylchroman-4-one (**2l**): Product obtained as a white solid in 62% [40]. m.p.: 124–126 °C. R_f_: 0.6 (ethyl acetate/hexane 3:7). IR (cm^−1^): 3063, 2843, 1691, 1670, 1602, 1572, 1509, 1452, 1264, 1236 1064, 1025. ^1^H-NMR (400 MHz, CDCl_3_): *δ* 3.85 (s, 3 H), 5.11 (d, *J* = 12.0 Hz, 1 H), 6.02 (d, *J* = 12.0 Hz, 1 H), 6.89 (d, *J* = 8.0 Hz, 2 H), 7.09–7.12 (m, 2 H), 7.30–7.37 (m, 3 H), 7.50–7.52 (m, 2 H), 7.57–7.60 (m, 1 H), 7.81 (d, *J* = 8.0 Hz, 2 H), 7,96 (dd, *J* = 8.0 Hz, *J* = 2.0 Hz, 1 H). ^13^C-NMR (75 MHz, CDCl_3_): *δ* 55.5, 59.3, 81.9, 113.8, 118.2, 120.7, 121.9, 127.3, 127.4, 128.7, 129.1, 130.8, 131.1, 136.7, 137.3, 161.3, 163.9, 189.9, 194.1. EI *m*/*z:* 260 (100%), 247 (50%), 203 (70%), 189 (30%), 130 (20%), 91 (40%). HRMS (ESI-TOF) *m*/*z* [M + H]^+^ Calculated for C_23_H_19_O_4_: 359.1278. Found: 359.1272.

Synthesis of trans-3-(4-methoxybenzoyl)-2-(4-methoxyphenyl)chroman-4-one (**2m**): Product obtained as a white solid in 96%. m.p.: 139–140 °C. R_f_: 0.6 (ethyl acetate/hexane 3:7). IR (cm^−1^): 3032, 2836, 1689, 1672, 1599, 1571, 1510, 1454, 1265, 1251, 1230, 1072, 1037, 1019. ^1^H-NMR (400 MHz, CDCl_3_): *δ* 3.78 (s, 3 H), 3.86 (s, 3 H), 5.10 (d, *J* = 12.0 Hz, 1 H), 5.96 (d, *J* = 12.0 Hz, 1 H), 6.85–6.91 (m, 4 H), 7.07–7.11 (m, 2 H), 7.43 (d, *J* = 8.0 Hz, 2 H), 7.54–7.58 (m, 1 H), 7.83 (d, *J* = 8.0 Hz, 2 H), 7.95 (d, *J* = 8.0 Hz, 1 H). ^13^C-NMR (100 MHz, CDCl_3_): *δ* 55.3, 55.3, 59.2, 81.6, 113.8, 114.1, 118.2, 120.6, 121.8, 127.4, 128.8, 129.4, 130.8, 131.1, 136.6, 159.9, 161.3, 163.9, 190.2, 194.2. HRMS (ESI-TOF) *m*/*z* [M + H]^+^ Calculated for C_24_H_21_O_5_: 389.1384. Found: 389.1369.

Synthesis of trans-2-(4-fluorophenyl)-3-(4-methoxybenzoyl)chroman-4-one (**2n**): Product obtained as a white solid in 54%. m.p.: 134–135 °C. R_f_: 0.6 (ethyl acetate/hexane 3:7). IR (cm^−1^): 3063, 2916, 1689, 1664, 1596, 1580, 1515, 1446, 1419, 1263, 1230, 1053, 1019. ^1^H-NMR (400 MHz, CDCl_3_): *δ* 3.86 (s, 3 H), 5.04 (d, *J* = 12.0 Hz, 1 H), 6.00 (d, *J* = 12.0 Hz, 1 H), 6.90 (d, *J* = 8.0 Hz, 2 H), 7.01–7.13 (m, 4 H), 7.48–7.52 (m, 2 H), 7.56–7.60 (m, 1 H), 7.81 (d, *J* = 8.0 Hz, 2 H), 7.96 (dd, *J* = 8.0 Hz, *J* = 2.0 Hz, 1 H). ^13^C-NMR (75 MHz, CDCl_3_): *δ* 55.6, 59.4, 81.2, 113.9, 115.6 and 115.8 (d, *J =* 22 Hz), 118.1, 120.6, 122.0, 127.5, 129.2 and 129.3 (d, *J =* 9 Hz), 130.7, 131.1, 133.2 and 133.3 (d, *J =* 3 Hz), 136.7, 161.1, 161.6 and 164.1 (d, *J =* 296 Hz), 164.0, 189.8, 193.9. HRMS (ESI-TOF) *m*/*z* [M − H]^−^ Calculated for C_23_H_16_FO_4_: 375.1038. Found: 375.1052.

Synthesis of trans-2-(2-chlorophenyl)-3-(4-methoxybenzoyl)chroman-4-one (**2o**): Product obtained as a white solid in 79%. m.p.: 173–175 °C. R_f_: 0.6 (ethyl acetate/hexane 3:7). IR (cm^−1^): 3061, 2837, 1696, 1651, 1597, 1571, 1457, 1430, 1265, 1230, 1056, 1032, 766. ^1^H-NMR (400 MHz, CDCl_3_): *δ* 3.88 (s, 3 H), 5.38 (d, *J* = 12.0 Hz, 1 H) 6.43 (d, *J* = 12.0 Hz, 1 H), 6.93 (d, *J* = 8.0 Hz, 2 H), 7.09–7.12 (m, 2 H), 7.23–7.28 (m, 2 H), 7.41–7.47 (m, 2 H), 7.56–7.61 (m, 1 H), 7.90–7.96 (m, 3 H). ^13^C-NMR (100 MHz, CDCl_3_): *δ* 55.6, 57.4, 78.4, 113.9, 118.0, 120.5, 121.9, 127.1, 127.5, 128.8, 130.3, 130.6, 131.3, 134.1, 134.4, 136.8, 161.2, 164.0, 189.4, 193.1. EI *m*/*z:* 260 (100%), 247 (50%), 203 (70%), 189 (30%), 130 (20%), 91 (40%). HRMS (ESI-TOF) *m*/*z* [M + H]^+^ Calculated for C_23_H_18_ClO_4_: 393.0888. Found: 393.0898.

Synthesis of trans-3-benzoyl-6-methoxy-2-phenylchroman-4-one (**2p**): Product obtained as a white solid in 93%. m.p.: 164–165 °C. R_f_: 0.6 (ethyl acetate/hexane 3:7). IR (cm^−1^): 3043, 2841, 1697, 1669, 1619, 1594, 1572, 1487, 1275, 1225, 1062, 1037. ^1^H-NMR (400 MHz, CDCl_3_): *δ* 3.84 (s, 3 H), 5.14 (d, *J* = 12.0 Hz, 1 H), 5.97 (d, *J* = 12.0 Hz, 1 H), 7.04 (d, *J* = 8.0 Hz, 1 H), 7.18–7.21 (m, 1 H), 7.30–7.37 (m, 5 H), 7.40–7.45 (m, 3 H), 7,49–7.56 (m, 3 H), 7.79–7.81 (m, 2 H). ^13^C-NMR (100 MHz, CDCl_3_): *δ* 55.9, 59.7, 82.1, 107.5, 119.5, 120.4, 126.0, 127.4, 127.8, 128.6, 128.6, 128.7, 129.1, 133.5, 137.2, 154.4, 156.0, 189.9, 196.3. HRMS (ESI-TOF) *m*/*z* [M + H]^+^ Calculated for C_23_H_19_O_4_: 359.1258. Found: 359.1266.

Synthesis of trans-3-benzoyl-6-methoxy-2-(4-methoxyphenyl)chroman-4-one (**2q**): Product obtained as a white solid in 92%, m.p.: 162–163 °C. R_f_: 0.6 (ethyl acetate/hexane 3:7). IR (cm^−1^): 3058, 2836,1692, 1675, 1613, 1580, 1514, 1486, 1275, 1249, 1225, 1076, 1029. ^1^H-NMR (400 MHz, CDCl_3_): *δ* 3.70 (s, 3 H), 3.83 (s, 3 H), 5.12 (d, *J* = 12.0 Hz, 1 H), 5.90 (d, *J* = 12.0 Hz, 1 H), 6.85 (d, *J* = 8.0 Hz, 2 H), 7.02 (d, *J* = 8.0 Hz, 1 H), 7.17–7.18 (m, 1 H), 7.35 (d, *J* = 8.0 Hz, 1 H), 7.41–7.45 (m, 4 H), 7.54–7.58 (m, 1 H), 7.82 (d, *J* = 8.0 Hz, 2 H). ^13^C-NMR (100 MHz, CDCl_3_): *δ* 55.3, 55.8, 59.6, 81.7, 107.5, 114.1, 119.5, 120.4, 125.9, 128.5, 128.6, 128.7, 128.6, 133.5, 137.7, 154.3, 156.1, 159.9, 190.2 196.4. HRMS (ESI-TOF) *m*/*z* [M + H]^+^ Calculated for C_24_H_21_O_5_: 389.1345. Found: 389.1354.

Synthesis of trans-3-benzoyl-2-(4-fluorophenyl)-6-methoxychroman-4-one (**2r**): Product obtained as a white solid in 88%, m.p.: 125–126 °C. R_f_: 0.6 (ethyl acetate/hexane 3:7). IR (cm^−1^): 3046, 2841, 1689, 1669, 1600, 1582, 1512, 1487, 1345, 1277, 1230, 1082, 1031. ^1^H-NMR (400 MHz, CDCl_3_): *δ* 3.84 (s, 3 H), 5.08 (d, *J* = 12.0 Hz, 1 H), 5.94 (d, *J* = 12.0 Hz, 1 H), 7.00–7.05 (m, 3 H), 7.18–7.21 (m,1 H), 7.36 (d, *J* = 8.0 Hz, 1 H), 7.41–7.51 (m, 4 H), 7.54–7.58 (m, 1 H), 7.80 (d, *J* = 8.0 Hz, 2 H). ^13^C-NMR (100 MHz, CDCl_3_): *δ* 55.9, 59.8, 81.4, 107.5, 115.6 and 115.8 (d, *J=* 22 Hz), 119.5, 120.4, 126.1, 128.6, 128.7, 129.2 and 129.3 (d, *J =* 8 Hz), 133.2 and 133.2 (d, *J =* 3 Hz). 133.7, 137.6, 154.5, 155.9, 161.6 and 164.1 (d, *J =* 263 Hz). 189.7, 196.2; HRMS (ESI-TOF) *m*/*z* [M − H] Calculated for C_23_H_16_FO_4_: 3675.1038. Found: 375.1027.

Synthesis of trans-3-benzoyl-2-(2-chlorophenyl)-6-methoxychroman-4-one (**2s**): Product obtained as a white solid in 89%, m.p.: 143–144 °C. R_f_: 0.6 (ethyl acetate/hexane 3:7). IR (cm^−1^): 3069, 2846, 1685, 1655, 1617, 1594, 1576, 1485, 1295, 1270, 1071, 1032, 760. ^1^H-NMR (400 MHz, CDCl_3_): *δ* 3.83 (s, 3 H), 5.41 (d, *J* = 12.0 Hz, 1 H), 5.38 (d, *J* = 12.0 Hz, 1 H), 7.04 (d, *J* = 8.0 Hz, 1 H), 7.18–7.27 (m, 1 H), 7.23–7.27 (m, 2 H), 7.36 (d, *J* = 8.0 Hz, 1 H), 7.40–7.48 (m, 4 H), 7.56–7.60 (m, 1 H), 7.90 (d, *J* = 8.0 Hz, 2 H). ^13^C-NMR (100 MHz, CDCl_3_): *δ* 55.9, 57.8, 78.5, 107.5, 119.4, 120.3, 126.1, 127.1, 128.5, 128.7, 128.8, 130.4, 130.6, 133.7, 134.1, 134.2, 137.2, 154.4, 155.9, 189.4, 195.3. HRMS (ESI-TOF) *m*/*z* [M + H]^+^ Calculated for C_23_H_18_ClO_4_: 393.0849. Found: 393.0888.

Synthesis of trans-3-benzoyl-6-chloro-2-phenylchroman-4-one (**2t**): Product obtained as a white solid in 87% [40]. m.p.: 138–139 °C. R_f_: 0.6 (ethyl acetate/hexane 3:7). IR (cm^−1^): 3039, 2895, 1697, 1672, 1600, 1579, 1470, 1450, 1275, 1042, 760. ^1^H-NMR (400 MHz, CDCl_3_): *δ* 5.15 (d, *J* = 12.0 Hz, 1 H), 6.00 (d, *J* = 12.0 Hz, 1 H), 7.07 (d, *J* = 8.0 Hz, 1 H), 7.31–7.44 (m, 5 H), 7.47–7.58 (m, 4 H), 7.80 (d, *J* = 8.0 Hz, 2 H), 7.91 (d, *J* = 8.0 Hz, 1 H). ^13^C-NMR (100 MHz, CDCl_3_): *δ* 59.3, 82.1, 119.9, 121.3, 126.6, 127.3, 127.5, 128.6, 128.7, 128.8, 129.3, 133.8, 136.6, 136.7, 137.4, 159.6, 188.9, 195.6. HRMS (ESI-TOF) *m*/*z* [M − H] Calculated for C_22_H_14_ClO_3_: 361.0637. Found: 361.0644.

Synthesis of trans-3-benzoyl-6-chloro-2-(4-methoxyphenyl)chroman-4-one (**2u**): Product obtained as a white solid in 76%, m.p.: 172 °C. R_f_: 0.6 (ethyl acetate/hexane 3:7). IR (cm^−1^): 3079, 2832, 1698, 1672, 1600, 1582, 1513, 1465, 1254, 1220, 1053, 1026, 760. ^1^H-NMR (400 MHz, CDCl_3_): *δ* 3.78 (s, 3 H), 5.12 (d, *J* = 12.0 Hz, 1 H), 5.92 (d, *J* = 12.0 Hz, 1 H), 6.86 (d, *J* = 8.0 Hz, 2 H), 7.05 (d, *J* = 8.0 Hz, 1 H), 7.40–7.59 (m, 6 H), 7.81 (d, *J* = 8.0 Hz, 2 H), 7.90 (d, *J* = 8.0 Hz, 1 H). ^13^C-NMR (100 MHz, CDCl_3_): *δ* 55.3, 59.2, 81.8, 114.2, 119.96, 121.3, 126.6, 127.4, 128.6, 128.7, 128.8, 128.8, 133.7, 136.5, 137.4, 159.7, 160.1, 189.1, 195.7. HRMS (ESI-TOF) *m*/*z* [M + H]^+^ Calculated for C_23_H_18_ClO_4_: 393.0888. Found: 393.0898.

Synthesis of trans-3-(furan-2-carbonyl)-2-phenylchroman-4-one (**2v**): Product obtained as a white solid in 95% [41]. m.p.: 175–176 °C. R_f_: 0.6 (ethyl acetate/hexane 3:7). IR (cm^−1^): 3094, 2897, 1686, 1657, 1602, 1570, 1502, 1459, 1248, 1146, 1045. ^1^H-NMR (400 MHz, CDCl_3_): *δ* 4.92 (d, *J* = 12.0 Hz, 1 H), 5.94 (d, *J* = 12.0 Hz, 1 H), 6.53 (d, *J* = 4.0 Hz, 1 H), 7.03−7.14 (m, 4 H), 7.17 (d, *J* = 4.0 Hz, 1 H), 7.49−7.60 (m, 5 H), 7.95 (d, *J* = 8.0 Hz, 1 H). ^13^C-NMR (100 MHz, CDCl_3_): *δ* 60.5, 60.57, 80.7, 112.9, 115.6, 115.9, 118.1, 120.5, 122.1, 127.5, 129.4, 132.9, 136.8, 147.3, 152.9, 161.1, 183.6, 189.1. HRMS (ESI-TOF) *m*/*z* [M + H]^+^ Calculated for C_20_H_15_O_4_: 319.0965. Found: 319.0976.

Synthesis of trans-3-(furan-2-carbonyl)-2-(4-methoxyphenyl)chroman-4-one (**2w**): Product obtained as a white solid in 91%. m.p.: 159–160 °C. R_f_: 0.6 (ethyl acetate/hexane 3:7). IR (cm^−1^): 3095, 2841, 1695, 1655, 1606, 1578, 1513, 1462, 1297, 1255, 1170, 1048, 1029. ^1^H-NMR (400 MHz, CDCl_3_): *δ* 3.80 (s, 3 H), 4.96 (d, *J* = 12.0 Hz, 1 H), 5.90 (d, *J* = 12.0 Hz, 1 H), 6.51 (d, *J* = 4.0 Hz, 1 H), 6.88 (d, *J* = 8.0 Hz, 2 H), 7.08−7.12 (m, 2 H), 7.17 (d, *J* = 8.0 Hz, 1 H), 7.44 (d, *J* = 8.0 Hz, 2 H), 7.55−7.57 (m, 2 H), 7.96 (d, *J* 8.0 Hz, 1 H). ^13^C-NMR (100 MHz, CDCl_3_): *δ* 55.3, 60.4, 81.1, 112.8, 114.1, 118.2, 118.5, 120.5, 121.9, 127.4, 128.8, 128.9, 136.6, 147.1, 153.1, 160.1, 161.3, 183.0, 189.5. HRMS (ESI-TOF) *m*/*z* [M − H] Calculated for C_21_H_15_O_5_: 347.0925. Found: 347.0921.

Synthesis of trans-2-(4-fluorophenyl)-3-(furan-2-carbonyl)chroman-4-one (**2x**): Product obtained as a white solid in 90%. m.p.: 171−172 °C. R_f_: 0.6 (ethyl acetate/hexane 3:7). IR (cm^−1^): 3096, 2886, 1698, 1650, 1603, 1584, 1519, 1454, 1305, 1226, 1151, 1042. ^1^H-NMR (400 MHz, CDCl_3_): *δ* 4.94 (d, *J* = 12.0 Hz, 1 H), 5.94 (d, *J* = 12.0 Hz, 1 H), 6.53 (dd, *J* = 16.4 Hz, *J* = 3.5 Hz, 1 H), 7.03−7.14 (m, 4 H), 7.17 (d, *J* = 4.0 Hz, 1 H), 7.49−7.60 (m, 4 H), 7.96 (dd, *J* = 8.0 Hz, *J* = 2.0 Hz, 1 H). ^13^C-NMR (100 MHz, CDCl_3_): *δ* 60.5, 80.7, 112.9, 115.6 and 115.9 (d, *J*= 22 Hz), 118.1, 118.6, 120.5, 122.1, 127.5, 129.3, 129.4 (d, *J =* 8 Hz), 132.8 and 132.9 (d, *J=* 3 Hz), 136.7, 147.3, 152.9, 161.1, 161.7 and 164.3 (d, *J*= 246 Hz), 183.6, 18.1. HRMS (ESI-TOF) *m*/*z* [M − H] Calculated for C_20_H_12_FO_4_: 335.0725. Found: 335.0758.

Synthesis of trans-3-benzoyl-2-(furan-2-yl)chroman-4-one (**2y**): Product obtained as a white solid in 24%. m.p.: 151–153 °C. R_f_: 0.6 (ethyl acetate/hexane 3:7). IR (cm^−1^): 3033, 2860, 1605, 1592, 1546, 1508, 1468, 1026. ^1^H-NMR (400 MHz, CDCl_3_): *δ* 6.24 (s, 1 H), 6.27−6.28 (m, 1 H), 6.34−6.35 (m, 1 H), 6.90 (d, *J* = 8.0 Hz, 1 H), 7.04−7.09 (m, 1 H), 7.40−7.45 (m, 8 H), 7.95 (dd, *J* = 8.0 Hz, *J* = 2.0 Hz, 1 H). ^13^C-NMR (100 MHz, CDCl_3_): *δ* 70.7, 103.0, 110.4, 111.4, 117.8, 120.7, 121.9, 126.3, 127.7, 128.6, 131.3, 134.5, 135.5, 143.6, 153.0, 157.0, 181.3, 182.7. HRMS (ESI-TOF) *m*/*z* [M + H]^+^ Calculated for C_20_H_15_O_4_: 319.0965. Found: 319.0956.

Synthesis of trans-3-benzoyl-2-(5-nitrofuran-2-yl)chroman-4-one (**2z**): Product obtained as a white solid in 32%. m.p.: 126–127 °C. R_f_: 0.6 (ethyl acetate/hexane 3:7). IR (cm^−1^): 3122, 2876, 1611, 1593, 1524, 1495, 1464, 1356, 1141. ^1^H-NMR (400 MHz, CDCl_3_): *δ* 6.32 (s, 1 H), 6.53 (d, *J* = 8.0 Hz, 1 H), 6.99 (d, *J* = 8.0 Hz, 1 H), 7.10–7.17 (m, 3 H), 7.45–7.56 (m, 6 H), 7.96 (d, *J* = 8.0 Hz, 1 H). ^13^C-NMR (100 MHz, CDCl_3_): *δ* 70.3, 101.3, 111.7, 113.6, 117.9, 120.2, 122.8, 126.5, 127.4, 128.9, 131.7, 134.0, 136.1, 155.9, 156.5, 180.7, 184.0. HRMS (ESI-TOF) *m*/*z* [M − H] Calculated for C_20_H_12_NO_6_: 362.0670. Found: 362.0673.

Synthesis of trans-3-benzoyl-2-methylchroman-4-one (**3**): Product obtained as a white solid in 25% [41]. m.p.: 90–91 °C. R_f_: 0.7 (ethyl acetate/hexane 3:7). IR (cm^−1^): 3060, 2842, 1702, 1674, 1605, 1577, 1517, 1449, 1370, 1222, 1072. ^1^H-NMR (400 MHz, CDCl_3_): *δ* 1,52 (d, *J* = 8.0 Hz, 3 H), 4.67 (d, *J* = 12.0 Hz, 1 H), 5.07–5.15 (m, 1 H), 7.04–7.08 (m, 2 H), 7.52–7.54 (m, 3 H), 7.63–7.67 (m, 1 H), 7.90 (d, *J* = 8.0 Hz, 1 H), 8.00 (d, *J* = 8.0 Hz, 2 H). ^13^C-NMR (100 MHz, CDCl_3_): *δ* 19.9, 59.9, 76.2, 117.9, 120.6, 121.6, 127.3, 128.8, 133.8, 136.5, 137.7, 161.3, 190.1, 196.1. HRMS (ESI-TOF) *m*/*z* [M + H]^+^ Calculated for C_17_H_15_O_3_: 267.1016. Found: 267.1004.

### 3.3. Synthesis of 3-Benzoyl-3-ethyl-2-phenylchroman-4-one (***4***)

Compound **2a** (1 mmol), anhydrous potassium carbonate (2 mmol) and iodoethane (1.5 mmol) were added to a round bottom flask containing acetone (15 mL) and stirred at 50 °C for 18 h. Upon completion, a liquid–liquid extraction was performed with ethyl acetate (2 × 20 mL) and water (20 mL). The organic layer was worked up in the usual way the product was purified by flash column chromatography, eluting with ethyl acetate and hexane. Product obtained as a yellow oil in 5% yield. R_f_: 0.5 (ethyl acetate/hexane 5:95). IR (cm^−1^): 3065, 2851, 1776, 1732, 1672, 1606, 1568, 1503, 1466, 1456, 1244 e 1069. ^1^H-NMR (400 MHz, CDCl_3_): *δ* 0.93 (t, 3 H), 3.62–3.79 (m, 2 H), 6.46 (s, 1 H), 6.90–6.96 (m, 2 H), 7.21–7.28 (m, 4 H), 7.40–7.47 (m, 5 H), 7.51–7.54 (m, 1 H), 7.84 (d, *J* = 8.0 Hz, 2 H). ^13^C-NMR (100 MHz, CDCl_3_): *δ* 15.1, 70.8, 77.5, 116.22, 117.2, 119.5, 121.4, 124.4, 127.1, 128.1, 128.3, 128.4, 129.1, 132.4, 132.5, 138.9, 139.8, 155.4, 155.7, 194.5. HRMS (ESI-TOF) *m*/*z* [M + H]^+^ Calculated for C_24_H_21_O_3_: 357.1484. Found: 357.1482.

### 3.4. Synthesis of 3,4-diphenyl-1,4-dihydrochromeno[4,3-c]pyrazole (***5***)

Compound **2a** (1 mmol), hydrazine (2 mmol), sodium acetate (2 mmol), and ethanol (10 mL) were added to a round bottom flask (50 mL) and stirred for 24 h at 70 °C. Next, the reaction as cooled and a liquid–liquid extraction was performed with ethyl acetate (2 × 20 mL) and water (20 mL). The product was concentrated on the rotary evaporator and purified by column chromatography, eluting with ethyl acetate and hexane. Product obtained as a yellow solid in 57%. m.p.: 155–156 °C. R_f_: 0.3 (ethyl acetate/hexane 2.5:7.5). IR (cm^−1^): 3450, 3044, 2923, 1618, 1574, 1509, 1471, 1300, 1212, 1035; ^1^H-NMR (400 MHz, CDCl_3_): *δ* 6.64 (s,1 H), 6.93–7.02 (m, 2 H), 7.17–7.21 (m, 1 H), 7.26–7.36 (m, 5 H), 7.32–7.37 (m, 6 H), 7.43 (d, *J* = 8.0 Hz, 1 H). ^13^C-NMR (100 MHz, CDCl_3_): *δ* 75.7, 111.1, 117.4, 117.9, 122.1, 122.8, 126.6, 127.9, 128.6, 128.6, 129.0, 129.6, 129.8, 139.7, 152.3. HRMS (ESI-TOF) *m*/*z* [M + H]^+^ Calculated for C_22_H_17_N_2_O: 325.1344. Found: 325.1335.

### 3.5. Anti-Trypanosoma cruzi Activity Assay (Amastigotes and Trypomastigotes)

The *in vitro* anti-*T. cruzi* activity was evaluated on L929 cells (mouse fibroblasts) infected with Tulahuen strain of the parasite expressing the *Escherichia coli β*-galactosidase as reporter gene. Briefly, for the bioassay, 4000 L929 cells were added to each well of a 96-well microtiter plate. After an overnight incubation, 40,000 trypomastigotes were added to the cells and incubated for 2 h. Then, the medium containing extracellular parasites were replaced with 200 μL of fresh medium and the plate was incubated for an additional 48 h to establish the infection. For IC_50_ determination, the cells were exposed to each synthesized compound at serial decreasing dilutions and the plate was incubated for 96 h. After this period, 50 μL of 500 μM chlorophenol red beta-d-galactopyranoside (CPRG) in 0.5% Nonidet P40 was added to each well, and the plate was incubated for 16–20 h, after which the absorbance at 570 nm was measured. Controls with uninfected cells, untreated infected cells, infected cells treated with Benznidazole at 3.8 μM (positive control) or DMSO 1% were used. The results were expressed as the percentage of *T. cruzi* growth inhibition in compound-tested cells as compared to the infected cells and untreated cells. The IC_50_ values were calculated by linear interpolation. Quadruplicates were run in the same plate, and the experiments were repeated at least once.

### 3.6. In Vitro Cytotoxic Test of Trypanocidal Compounds

The active compounds were tested *in vitro* for determination of cellular toxicity against uninfected L-929 cells using the alamarBlue^®^ dye. The cells were exposed to compounds at increasing concentrations starting at IC_50_ value for *T. cruzi*. After 96 h of incubation with the tested compounds, the alamarBlue^®^ was added and the absorbance at 570 and 600 nm measured after 4–6 h. The cell viability was expressed as the percentage of difference in the reduction between treated and untreated cells. IC_50_ values were calculated by linear interpolation, and the selectivity index (SI) was determined based on the ratio of the IC_50_ value in the host cell divided by the IC_50_ value of the parasite. Quadruplicates were run in the same plate, and the experiments were repeated at least once.

## 4. Conclusions

In conclusion, the anti *T. cruzi* activity of 28 3-benzoylflavanones were evaluated *in vitro* against amastigote and trypomastigote forms of parasite. Most of the evaluated compounds presented promising activity against the intracellular forms of *T. cruzi*. In particular, flavanone **2z** bearing a nitrofuran moiety displayed and IC_50_ value was lower than the reference drug Benznidazole. For this reason, the *in vivo* evaluation of the lead compound is in planning. Further investigations involving colorimetric methods and indirect analysis by light microscopy to discern the mechanism of action of the nitrofuran bearing flavanone are underway and will be disclosed in a follow-up report.

## Figures and Tables

**Table 1:** In vitro trypanocidal activity, cytotoxicity, selectivity index, and physicochemical properties of bioactive flavanones.

	In Vitro Activity			Lipinski’s Rule of Five								Risk Toxicity			
Compound	Trypanocide IC_50_(μM) ± 0.2	Cytotoxicity CC_50_(μM) ± 2.0	SI	HBA	HBD	MW (g.mol^−1^)	log P	Violations	TPSA (A^2^)	Volume A^3^	NRB	M	T	I	RE
**2a**	8.8	80	9.1	3	0	328.37	4.43	0	43.38	296.63	3	NR	NR	NR	NR
**2b**	10.74	441.90	40.3	3	0	362.81	5.06	1	43.38	310.16	3	NR	NR	NR	NR
**2c**	21.95	231.14	10.5	3	0	346.36	4.59	0	43.38	301.56	3	NR	NR	NR	NR
**2d**	5.8	223.40	38.5	4	0	358.39	4.49	0	52.61	322.17	4	NR	NR	NR	NR
**2e**	183.90	243.10	1.3	4	0	329.36	3.26	0	56.27	292.47	3	NR	NR	NR	NR
**2f**	7.60	92.13	12.1	4	0	434.39	6.08	1	52.61	393.82	6	NR	NR	NR	NR
**2g**	Inactive	-	-	3	0	342.39	4.88	0	43.38	313.19	3	NR	NR	NR	NR
**2h**	22.49	111.10	4.9	3	0	360.38	5.04	1	43.38	318.12	3	NR	NR	NR	NR
**2i**	24.99	107.48	4.3	4	0	372.42	4.93	0	52.61	338.73	4	NR	NR	NR	NR
**2j**	63.02	62.48	1	3	0	376.84	5.51	1	43.38	326.72	3	NR	NR	NR	NR
**2k**	29.30	110.50	3.8	3	0	362.81	5.11	1	43.38	310.16	3	NR	NR	NR	NR
**2l**	223.40	223.40	1	4	0	358.39	4.49	0	52.61	322.17	4	NR	NR	NR	NR
**2m**	37.35	103.05	2.8	5	0	388.42	4.54	0	61.84	347.72	5	NR	NR	NR	NR
**2n**	212.70	-	-	4	0	376.38	4.65	0	52.61	327.10	4	NR	NR	NR	NR
**2o**	26.52	408.10	15.4	4	0	392.84	5.12	1	52.61	335.71	4	NR	NR	NR	NR
**2p**	Inactive	-	-	4	0	358.39	4.46	0	52.61	322.17	4	NR	NR	NR	NR
**2q**	141.18	206.12	1.4	5	0	388.42	4.52	0	61.84	347.72	5	NR	NR	NR	NR
**2r**	17.55	106.35	6.1	4	0	376.38	4.63	0	52.61	327.10	4	NR	NR	NR	NR
**2s**	18.36	102.02	5.5	4	0	392.84	5.09	1	52.61	335.71	4	NR	NR	NR	NR
**2t**	23.20	220.95	9.5	3	0	362.81	5.08	1	43.38	310.16	3	NR	NR	NR	NR
**2u**	59.17	204.95	3.5	4	0	392.84	5.14	1	52.61	335.71	4	NR	NR	NR	NR
**2v**	Inactive	-	-	4	0	318.33	3.69	0	56.52	278.19	3	NR	NR	NR	NR
**2w**	Inactive	-	-	5	0	348.35	3.74	0	65.75	303.74	4	NR	NR	NR	NR
**2x**	Inactive	-	-	4	0	336.32	3.85	0	56.52	283.12	3	NR	NR	NR	NR
**2y**	113.80	503.0	4.4	4	1	318.33	4.72	0	59.67	277.82	3	NR	NR	NR	NR
**2z**	2.6	27.54	10.6	7	1	363.32	4.80	0	105.50	301.15	6	LR	NR	NR	NR
**3**	Inactive	-	-	3	0	266.30	3.21	0	43.38	241.78	2	NR	NR	NR	NR
**4**	13.2	449.26	34.03	3	0	256.43	5.01	1	43.38	329.67	4	NR	NR	NR	NR
**5**	8.3	38.56	4.6	3	1	324.38	5.01	1	37.92	294.07	2	NR	NR	NR	NR
**Bnz**	3.8	2381	625	-	-	260.25	0.78	0	92.75	224.99	5	NR	NR	NR	HR

IC_50_: 50% inhibitory concentration. CC_50_: 50% cytotoxic concentration determined using mammalian L929 cells. SI: selectivity index calculated from CC_50_/IC_50_. LogP: octanol/water partition coefficient, TPSA: total polar surface area, HBA = hydrogen bond acceptors, HBD = hydrogen bond donors, NRB = number of rotatable bonds, M = mutagenicity, T = tumorigenicity, I = skin irritation, RE = reproductive effect, NR = no risk, LR = low risk, MR = medium risk, HR = high risk.

## Supplementary Materials

The supplementary materials are available online.
