# Supplementary material for: Trypanocidal Activity of Flavanone Derivatives
Source: Molecules. 2020 Jan 17;25(2):397. doi: 10.3390/molecules25020397 (PMC7024391; doi:10.3390/molecules25020397)

The ^{1}H NMR (400 MHz), ^{13}C NMR (100 MHz) spectra of Compound **2a** in $CDCl_3$

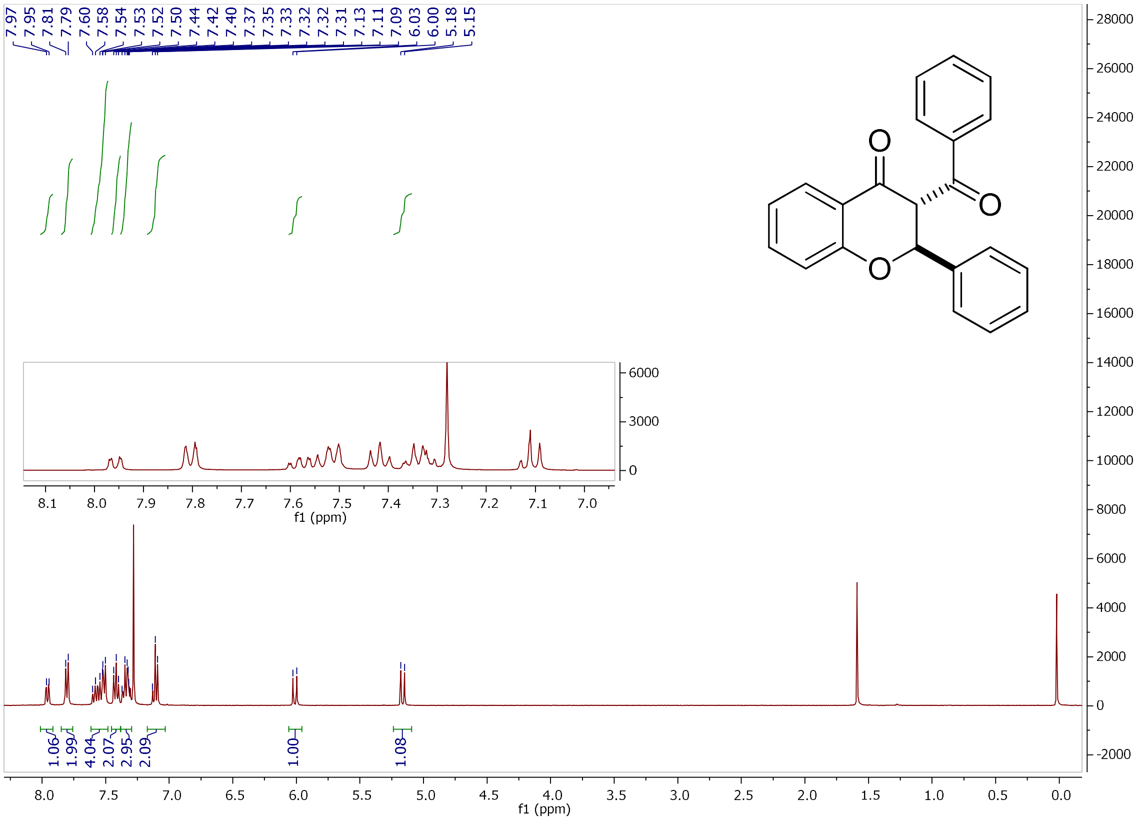

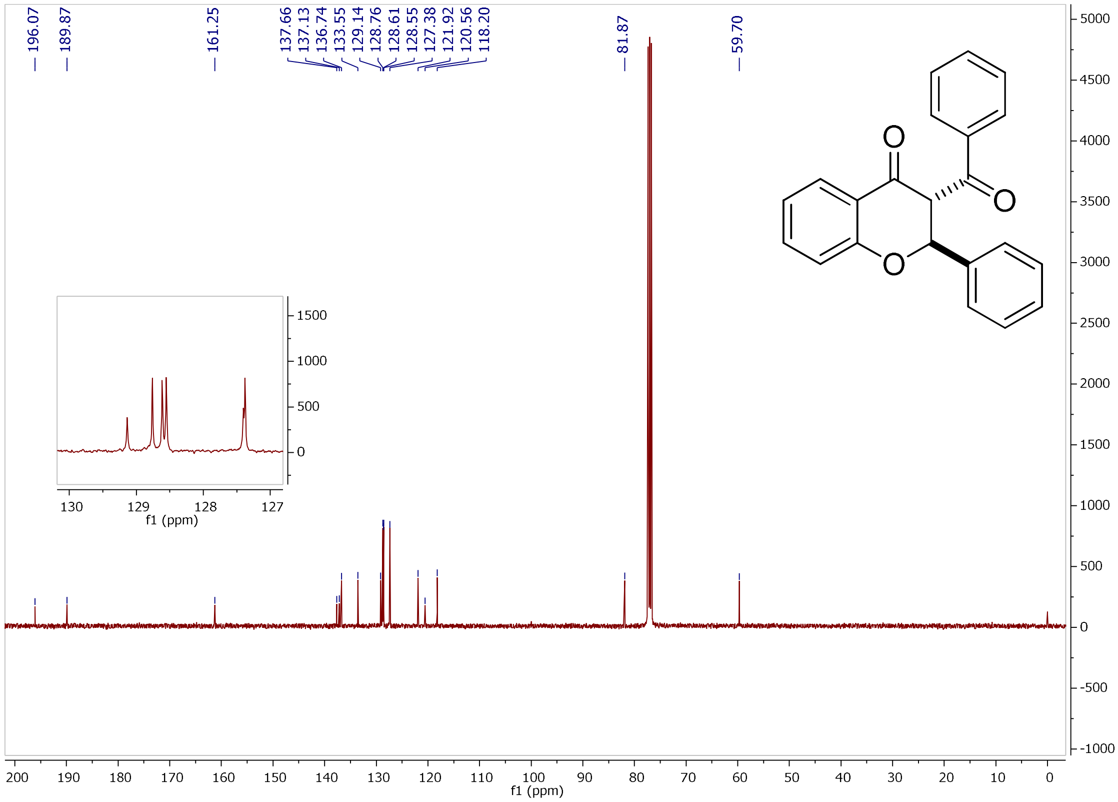

The ^{1}H NMR (400 MHz), ^{13}C NMR (100 MHz) spectra of Compound **2b** in $CDCl_3$

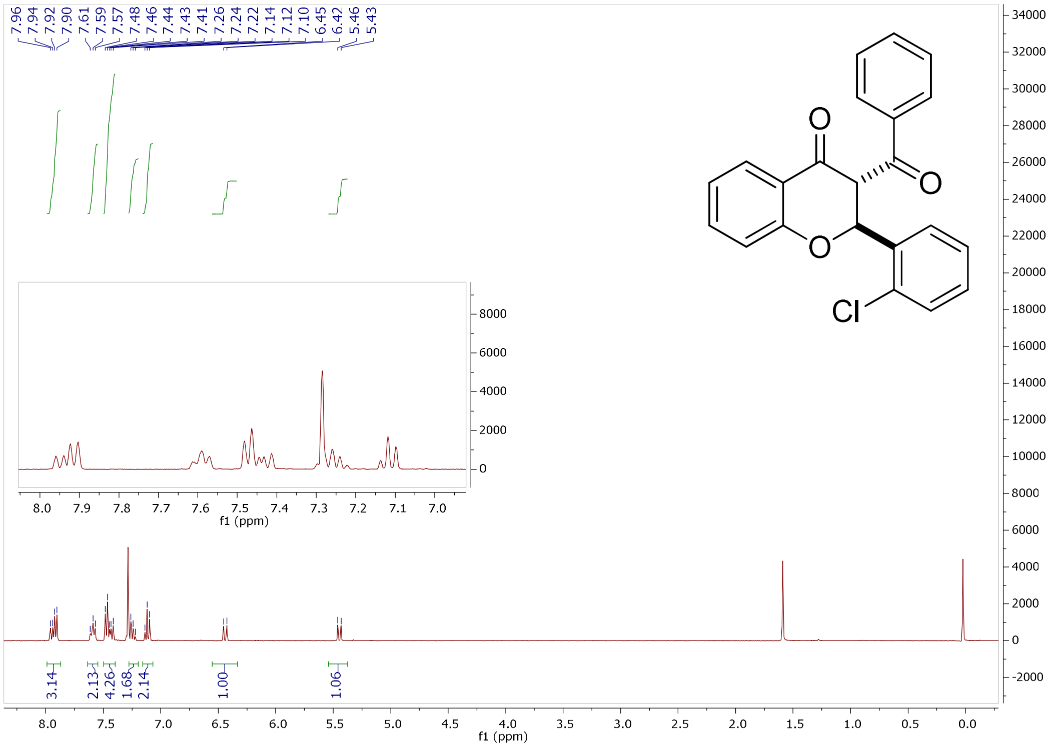

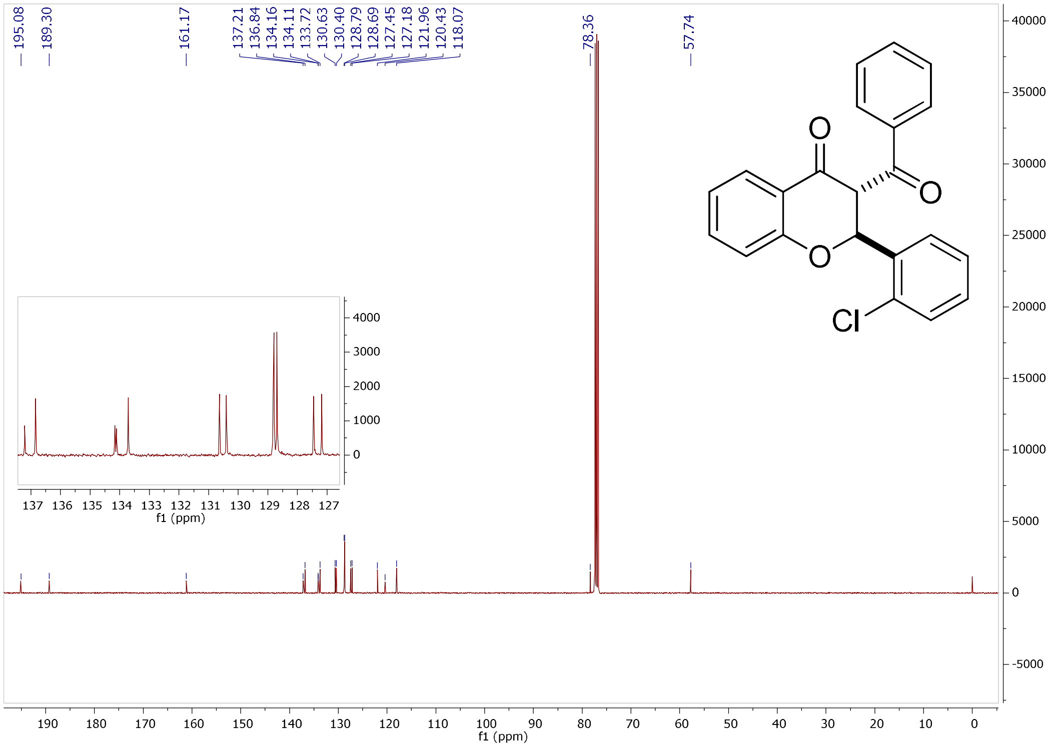

The ^{1}H NMR (400 MHz), ^{13}C NMR (100 MHz) spectra of Compound **2c** in $CDCl_3$

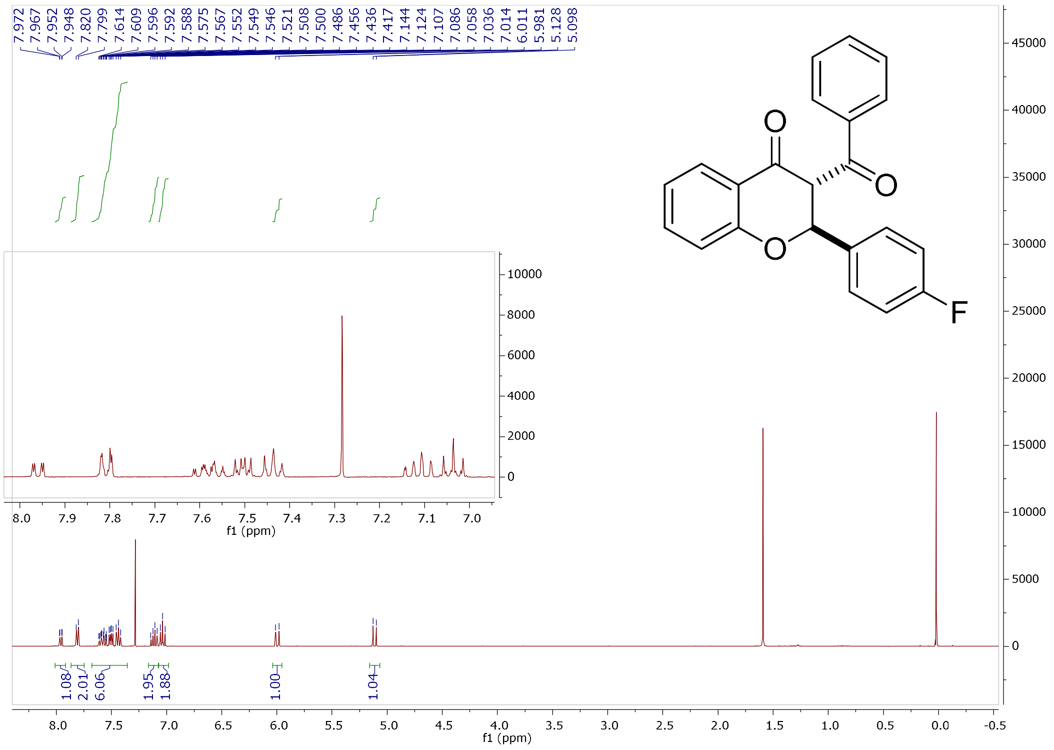

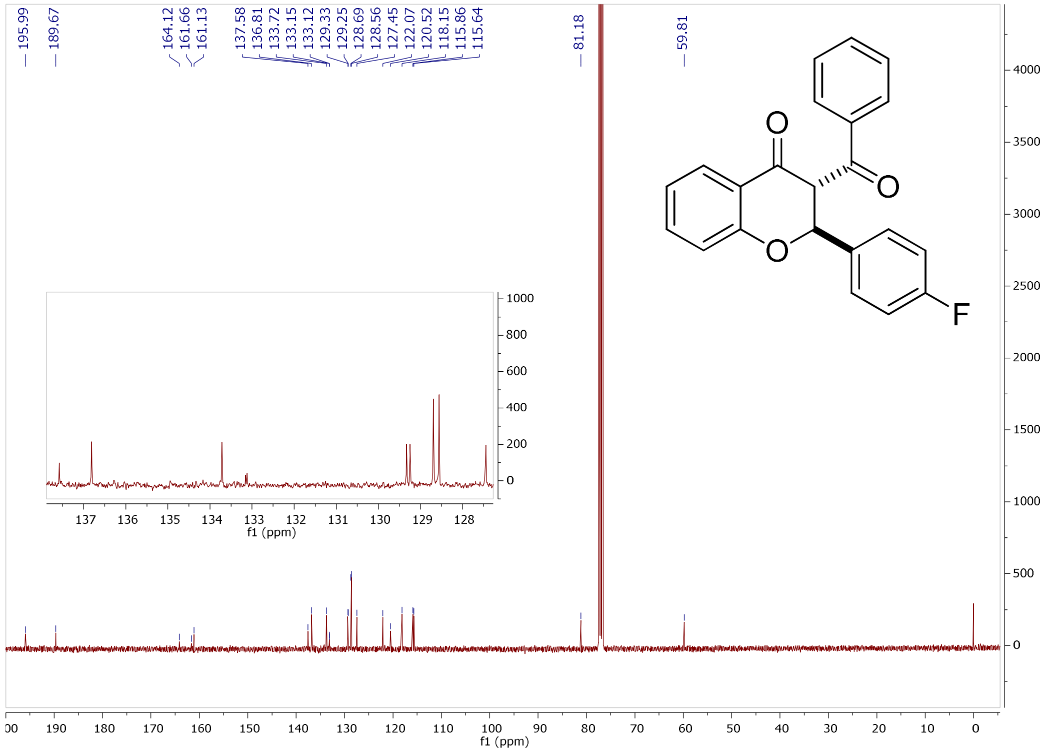

The ^{1}H NMR (400 MHz), ^{13}C NMR (100 MHz) spectra of Compound **2d** in $CDCl_3$

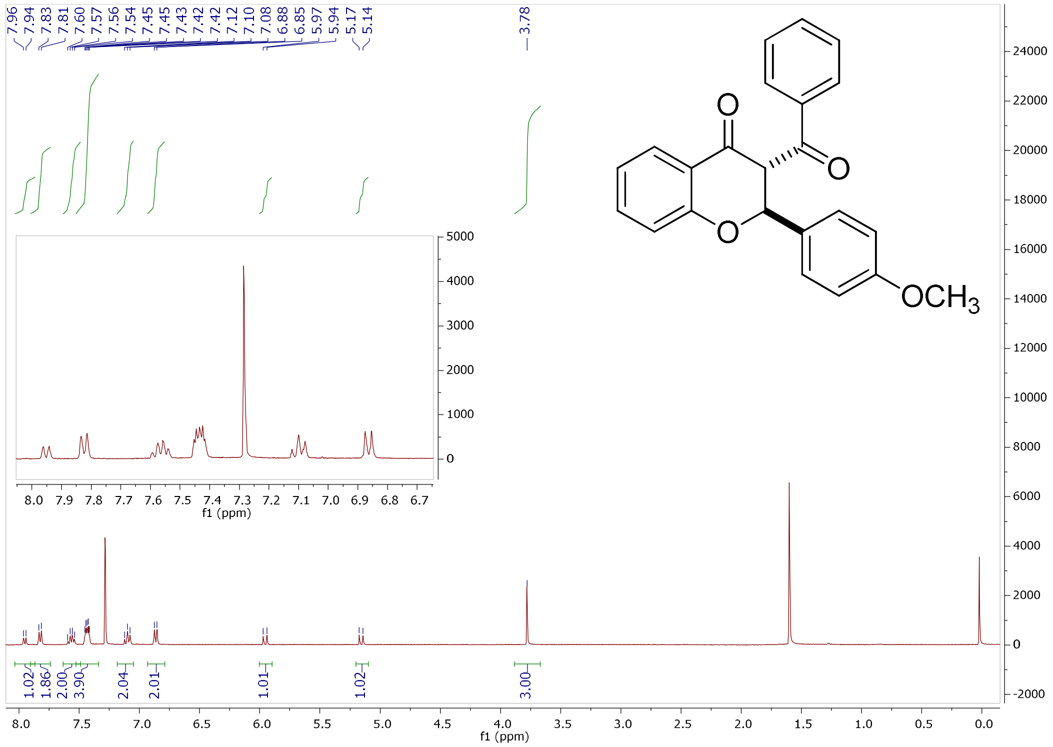

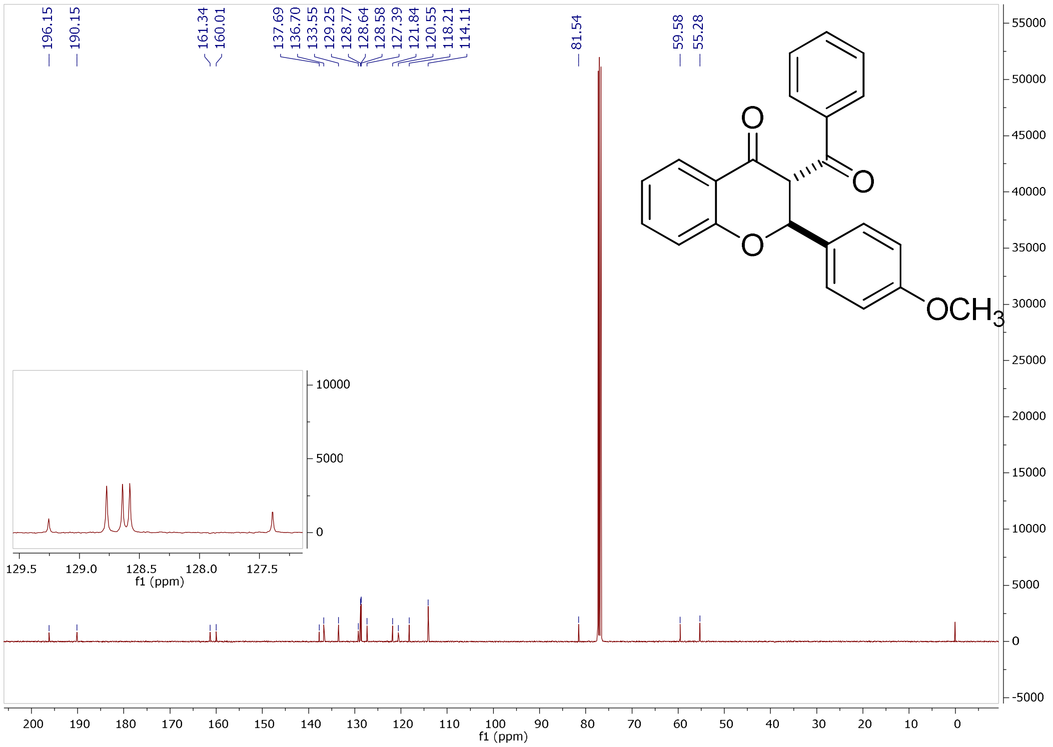

The ^{1}H NMR (400 MHz), ^{13}C NMR (100 MHz) spectra of Compound **2e** in $CDCl_3$

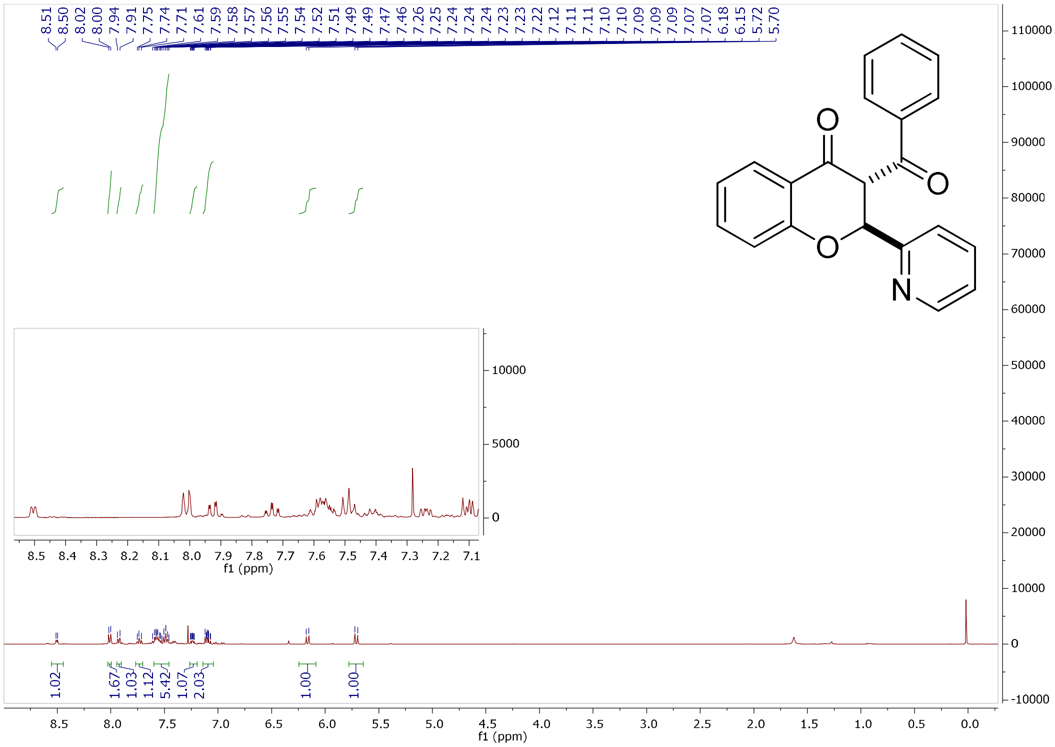

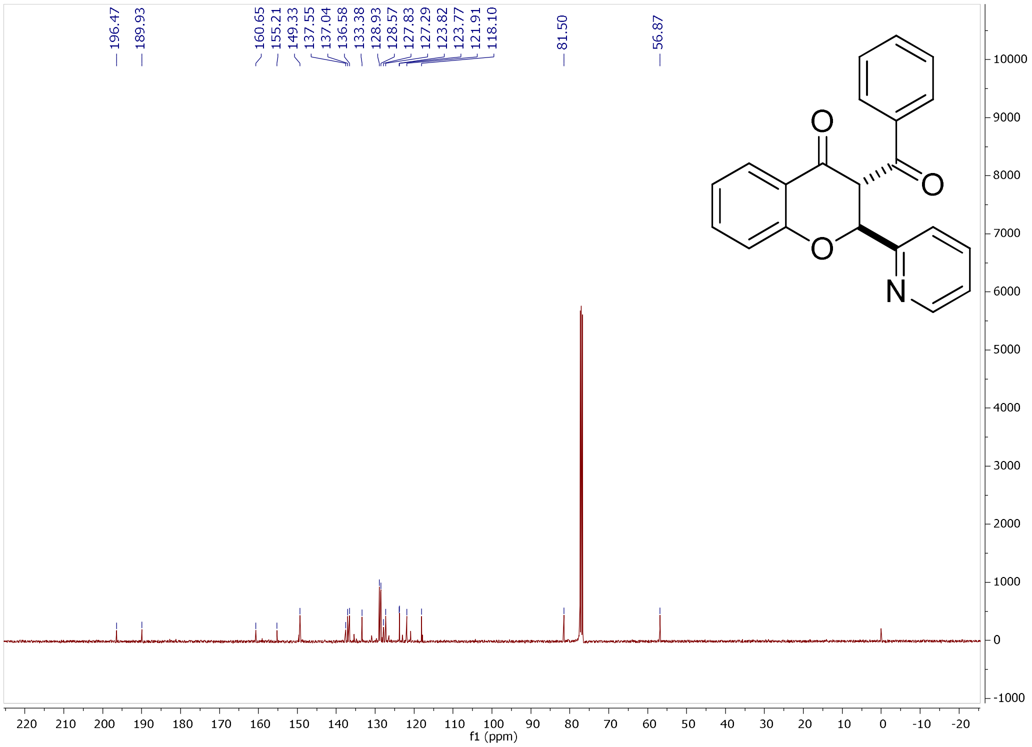

The ^{1}H NMR (400 MHz), ^{13}C NMR (100 MHz) spectra of Compound **2f** in $CDCl_3$

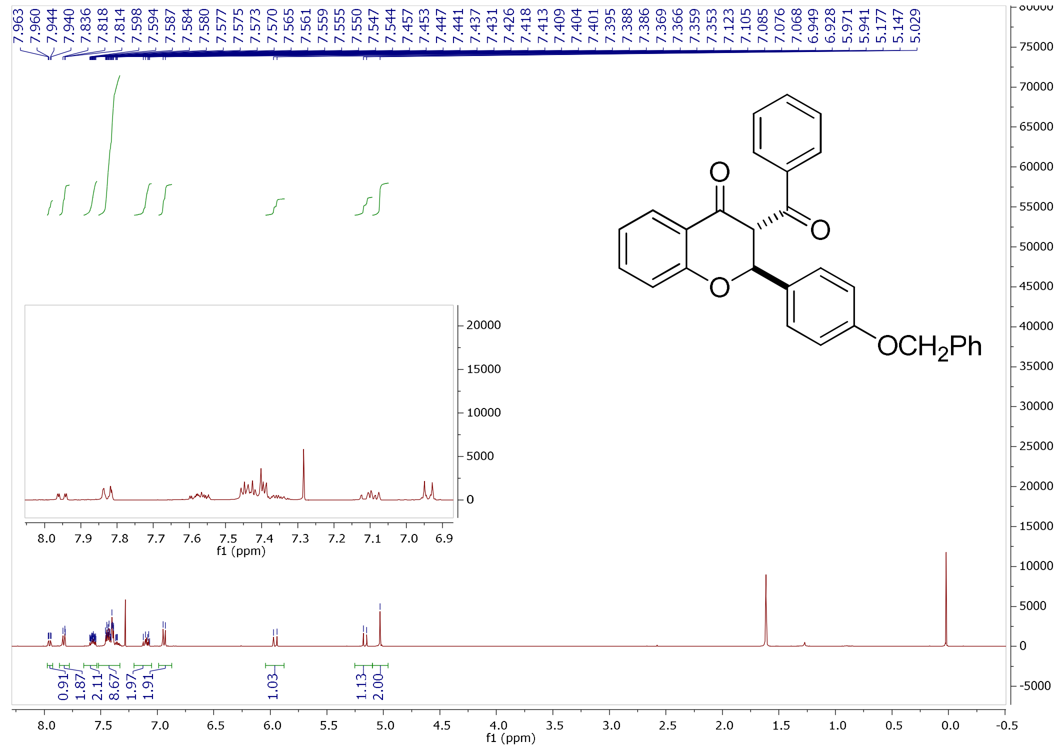

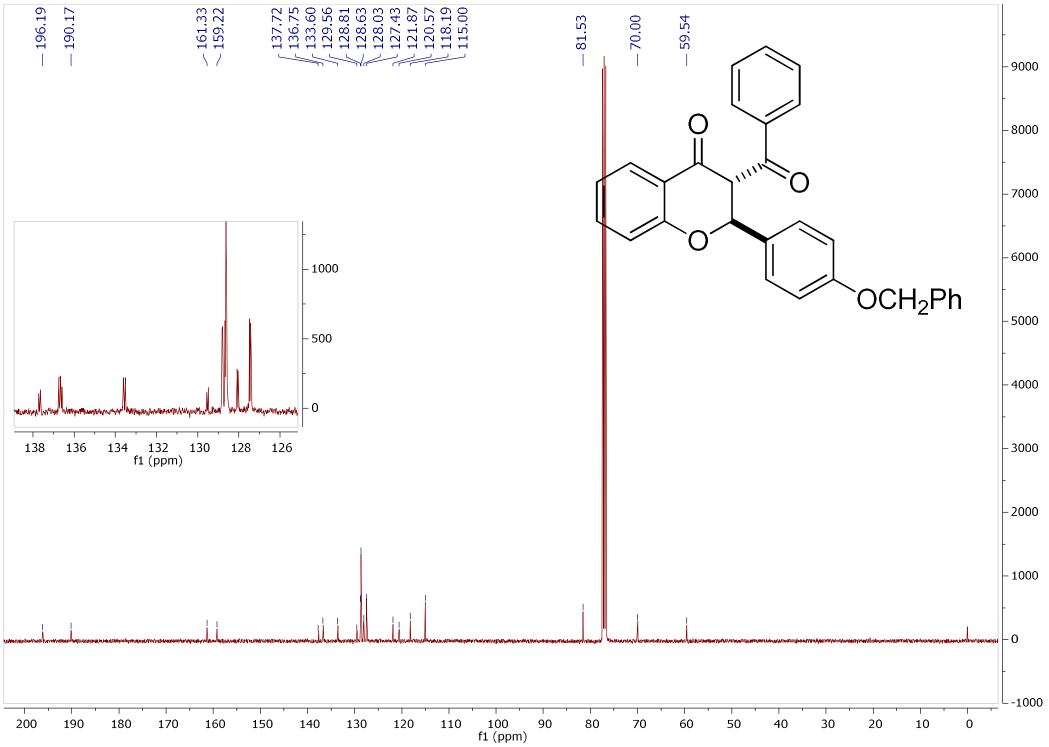

The ^{1}H NMR (400 MHz), ^{13}C NMR (100 MHz) spectra of Compound **2g** in $CDCl_3$

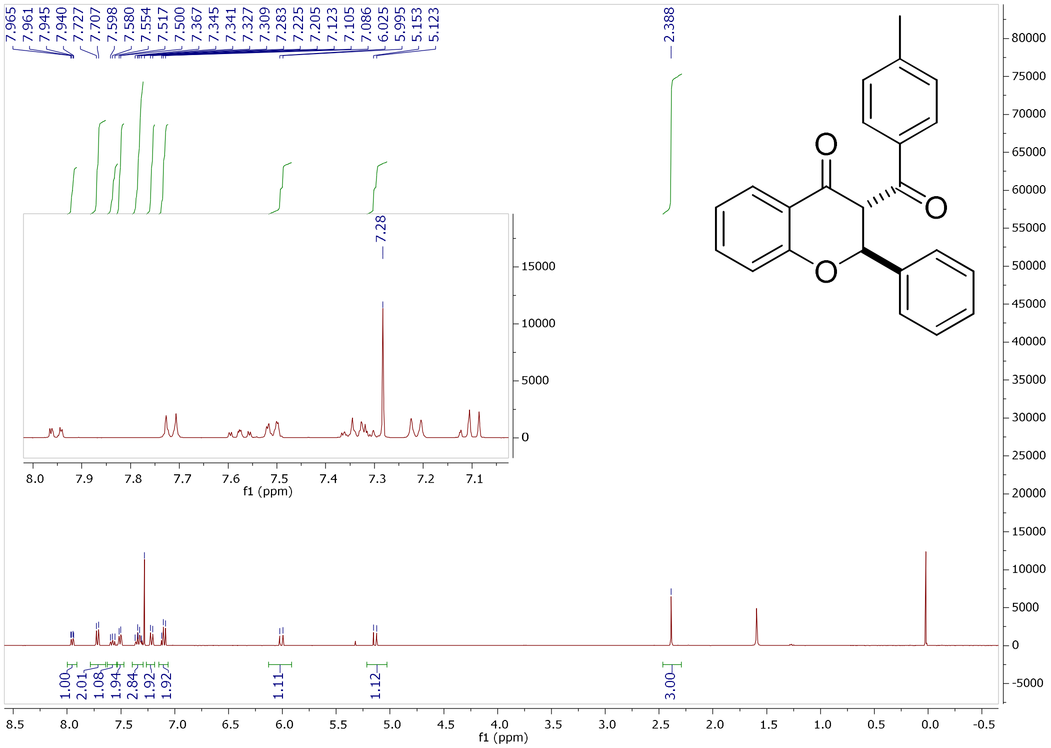

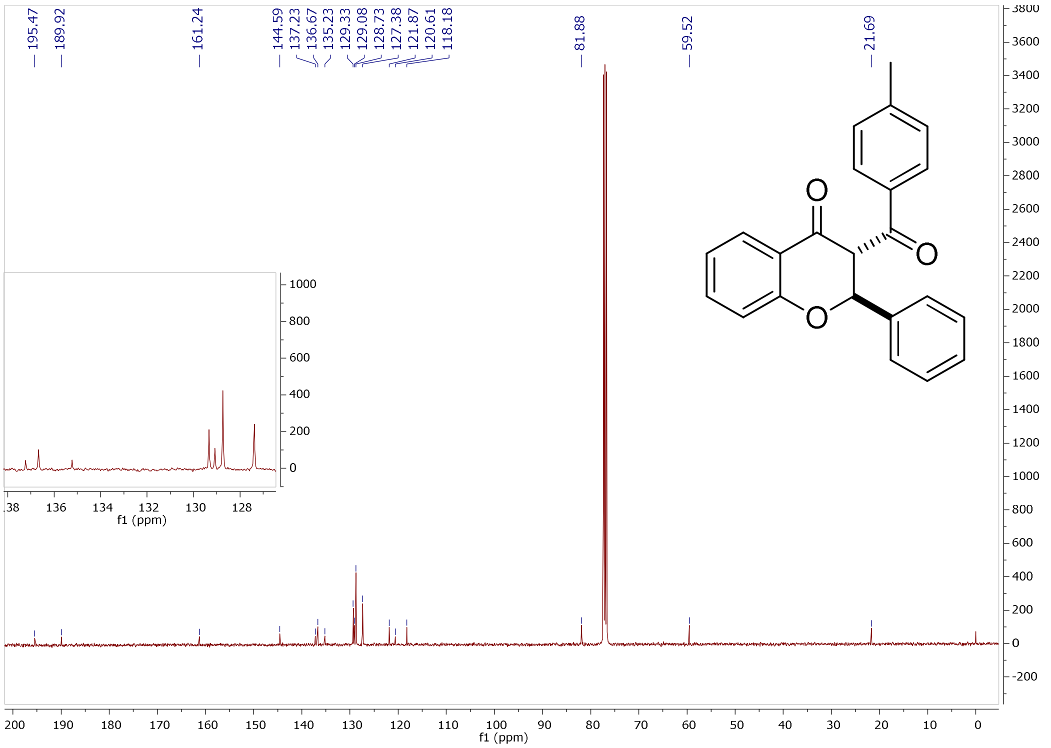

The ^{1}H NMR (400 MHz), ^{13}C NMR (100 MHz) spectra of Compound **2h** in $CDCl_3$

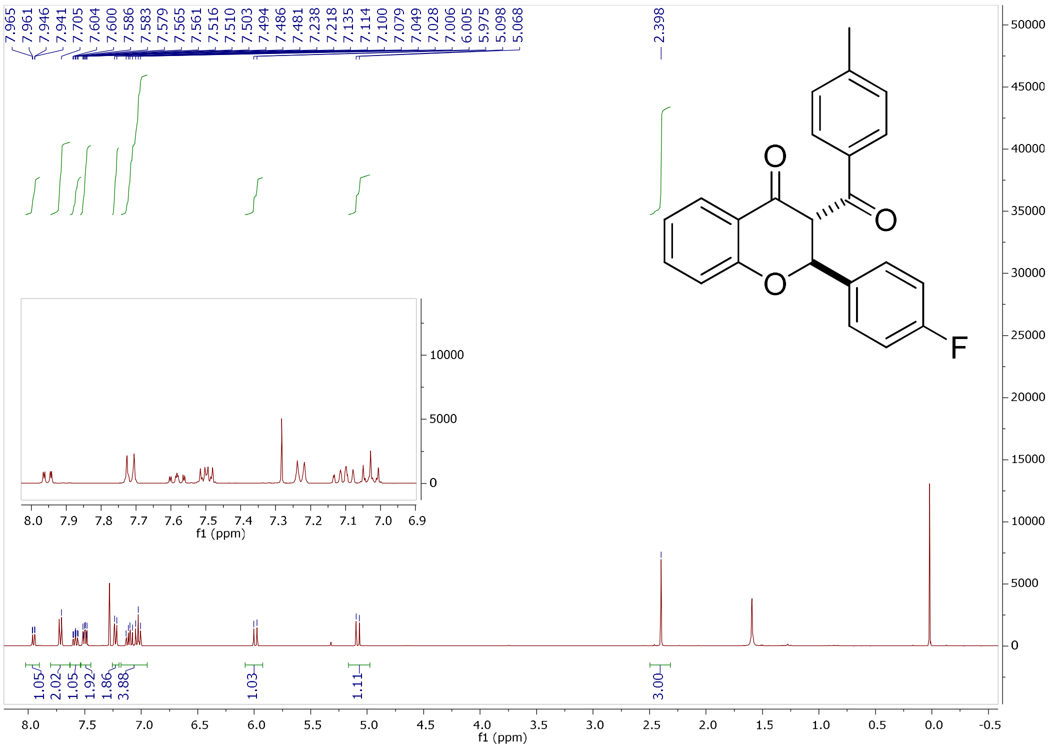

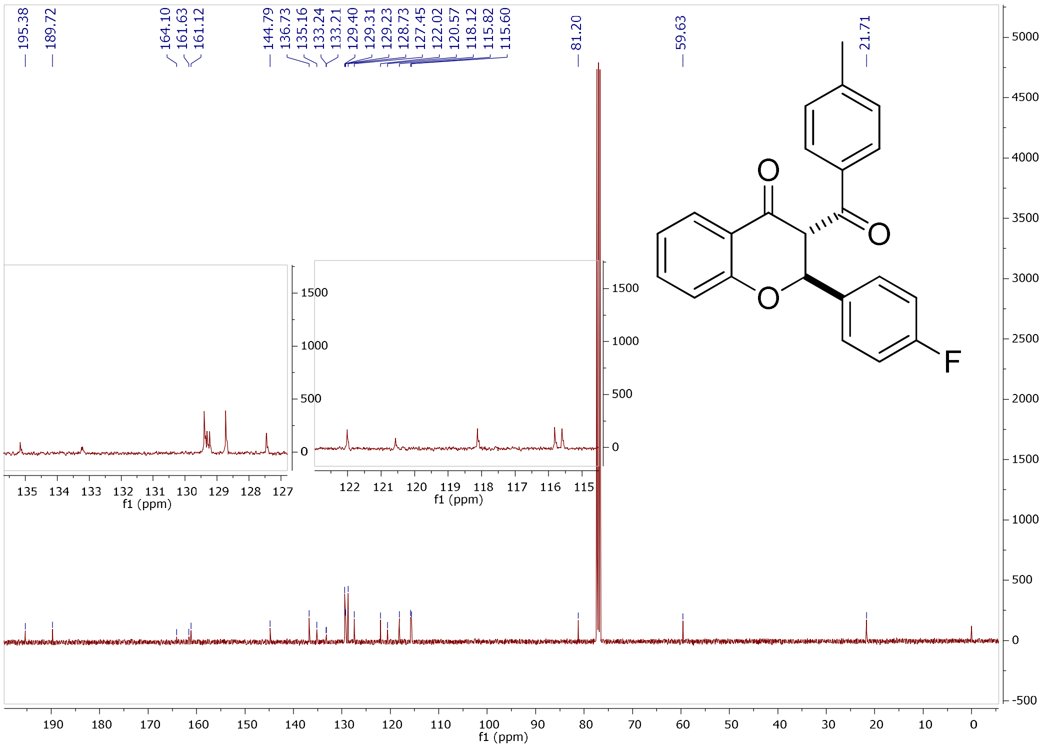

The ^{1}H NMR (400 MHz), ^{13}C NMR (100 MHz) spectra of Compound **2i** in $CDCl_3$

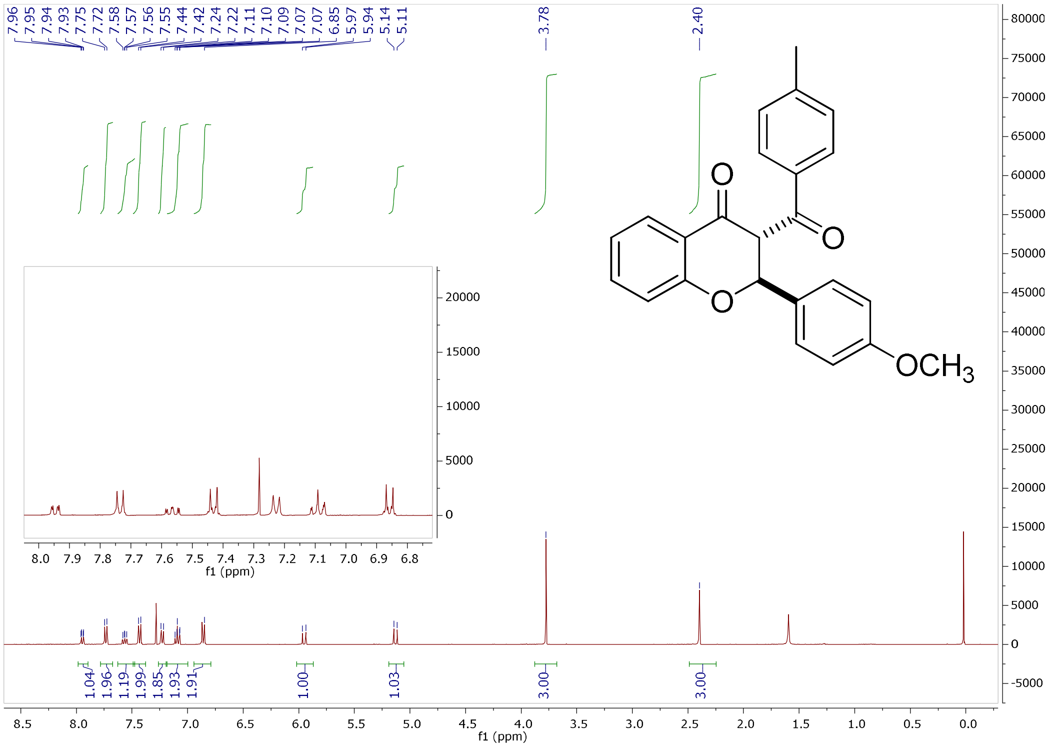

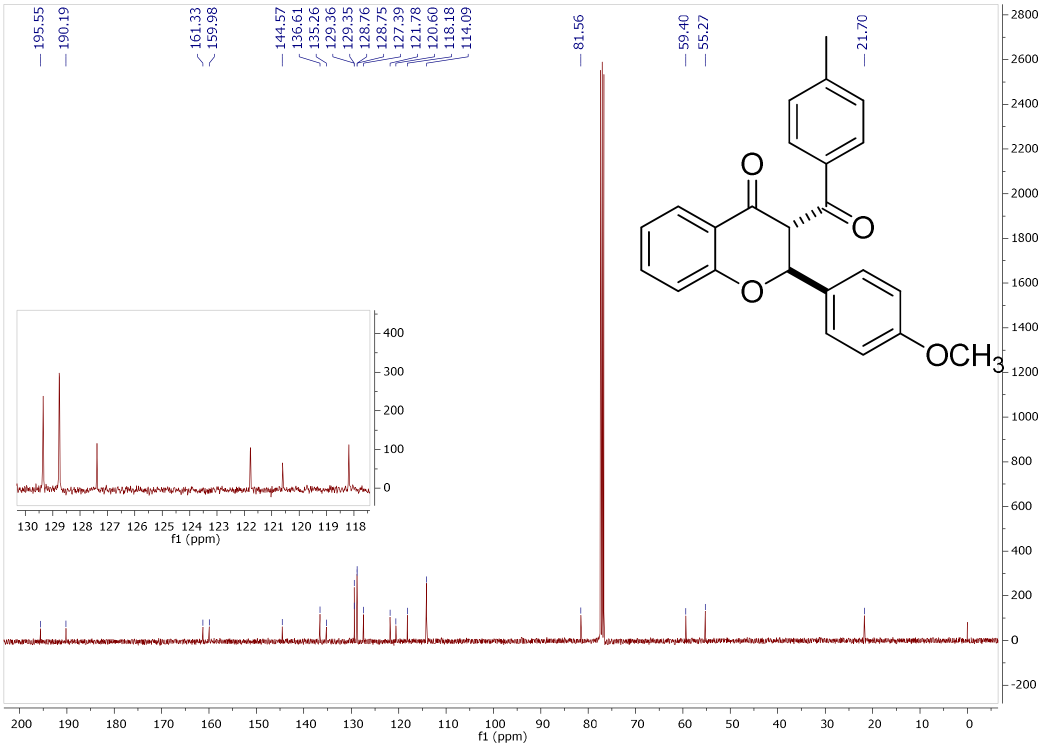

The ^{1}H NMR (400 MHz), ^{13}C NMR (100 MHz) spectra of Compound **2j** in $CDCl_3$

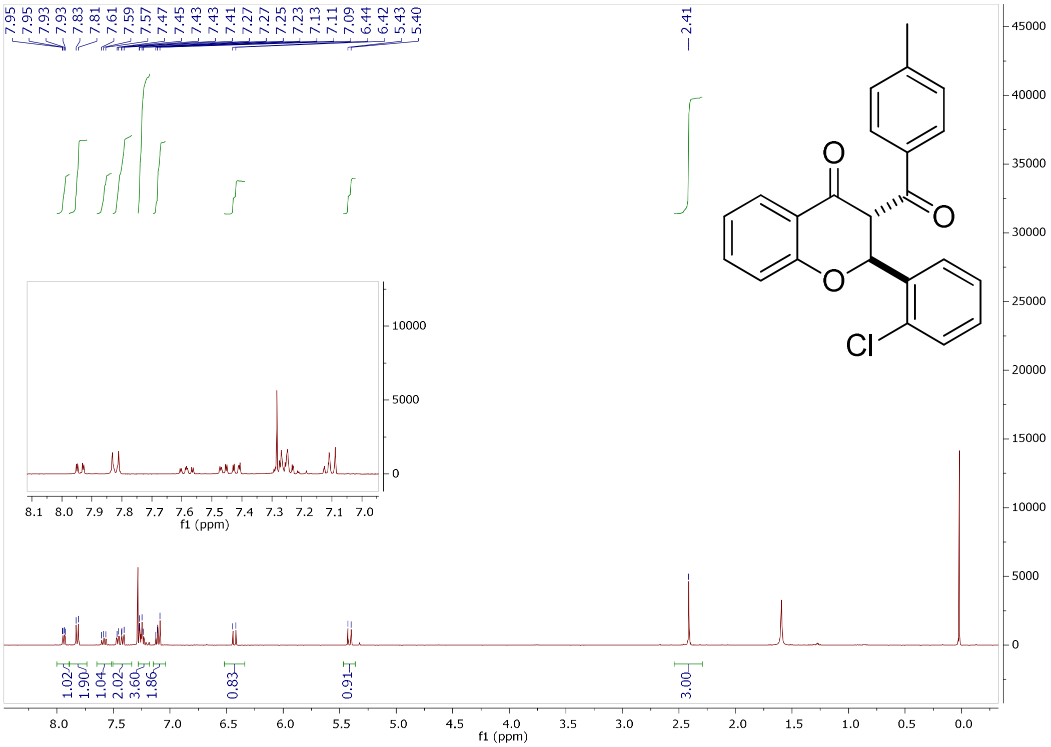

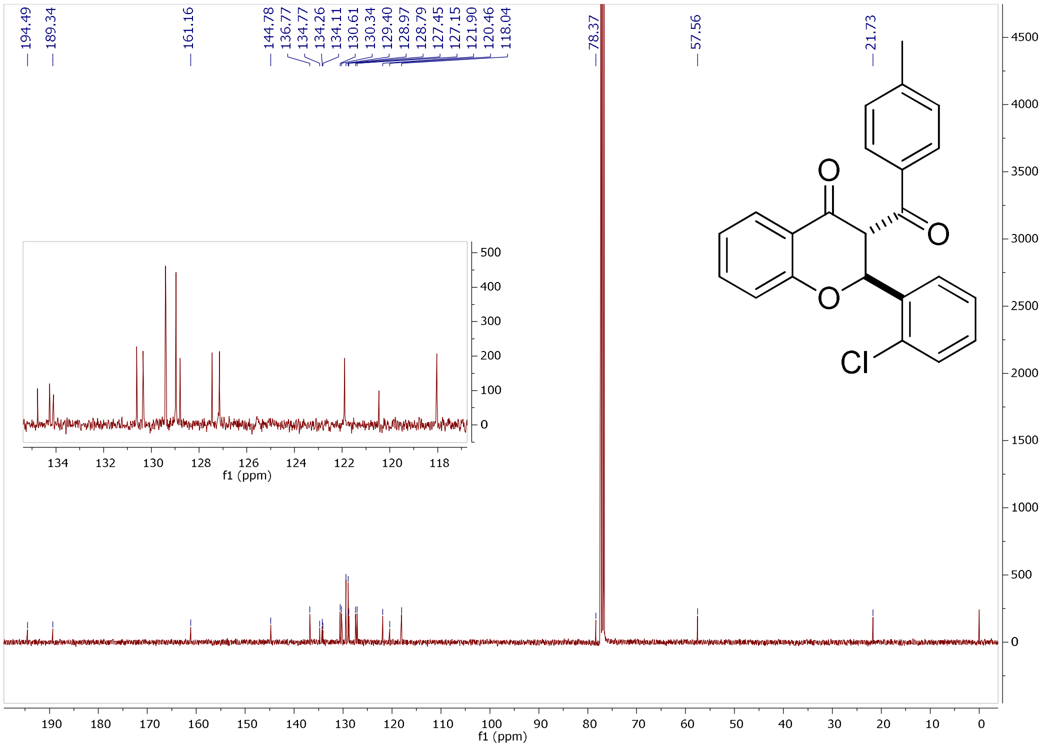

The ^{1}H NMR (400 MHz), ^{13}C NMR (100 MHz) spectra of Compound **2k** in $CDCl_3$

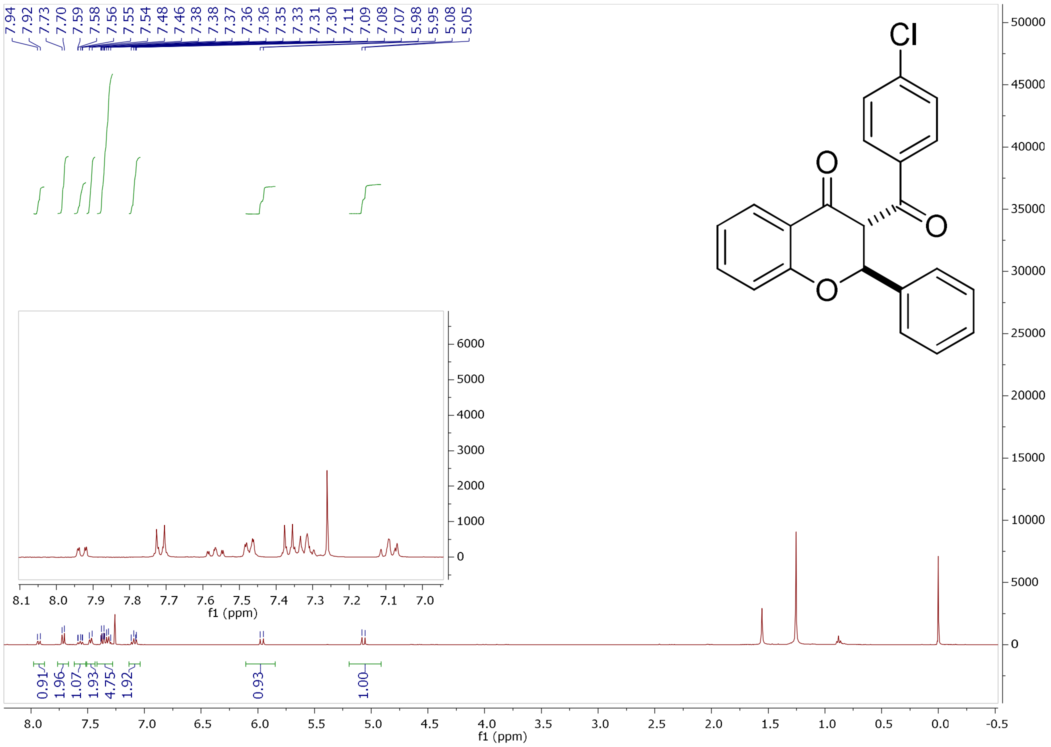

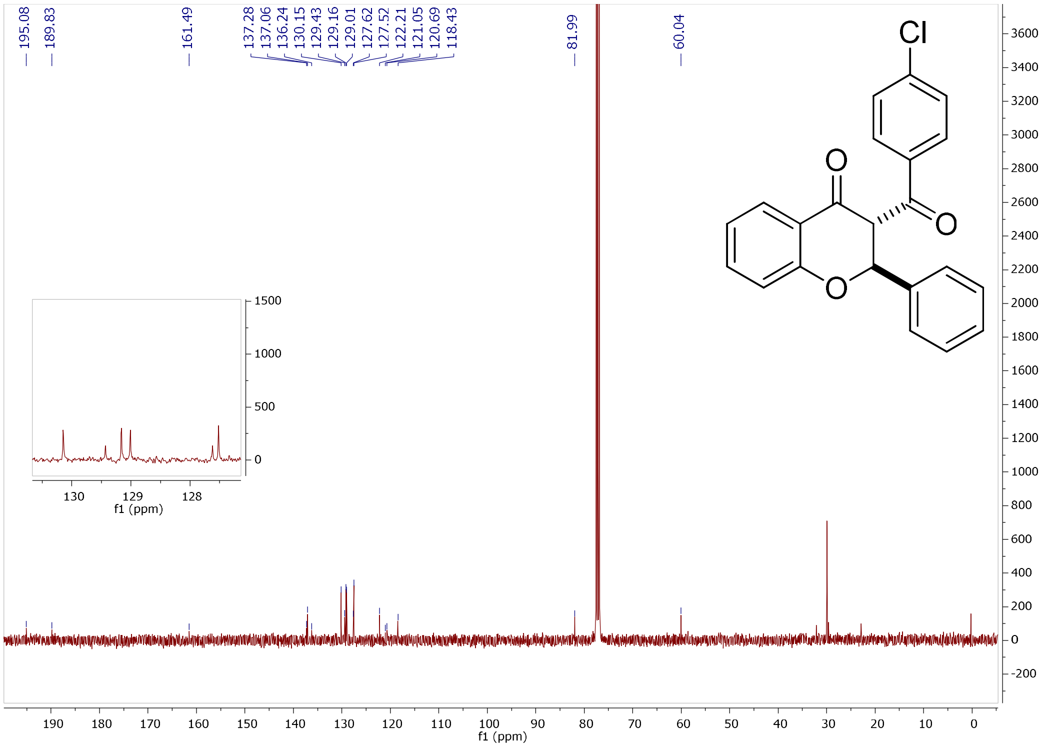

The ^{1}H NMR (400 MHz), ^{13}C NMR (100 MHz) spectra of Compound **2l** in $CDCl_3$

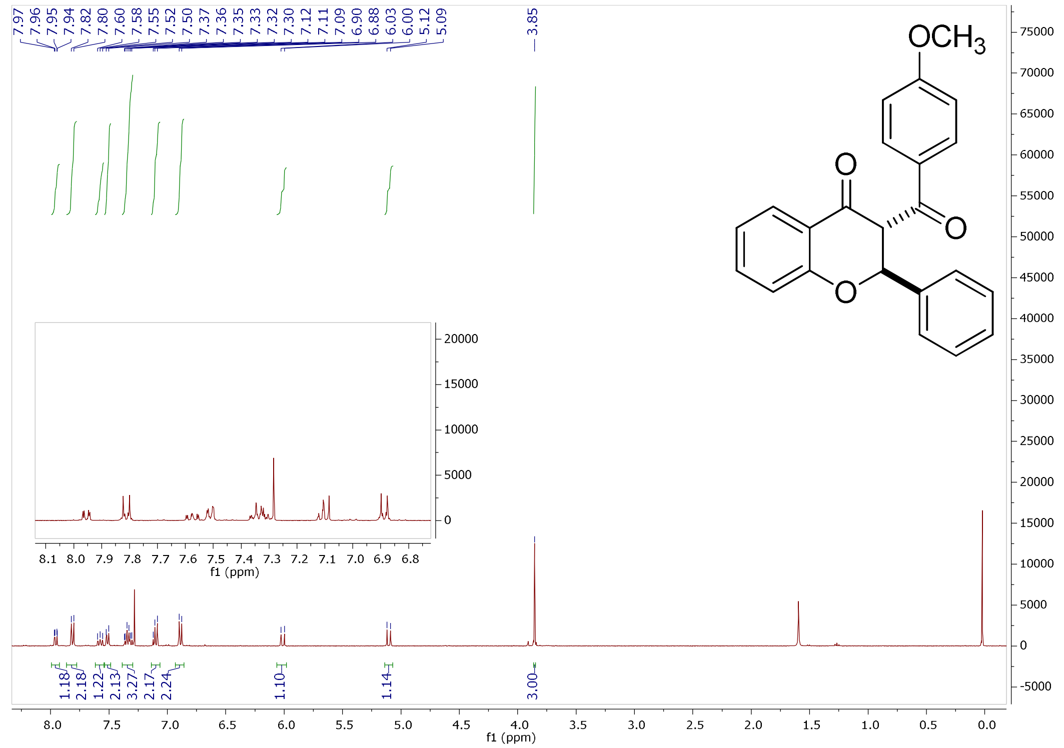

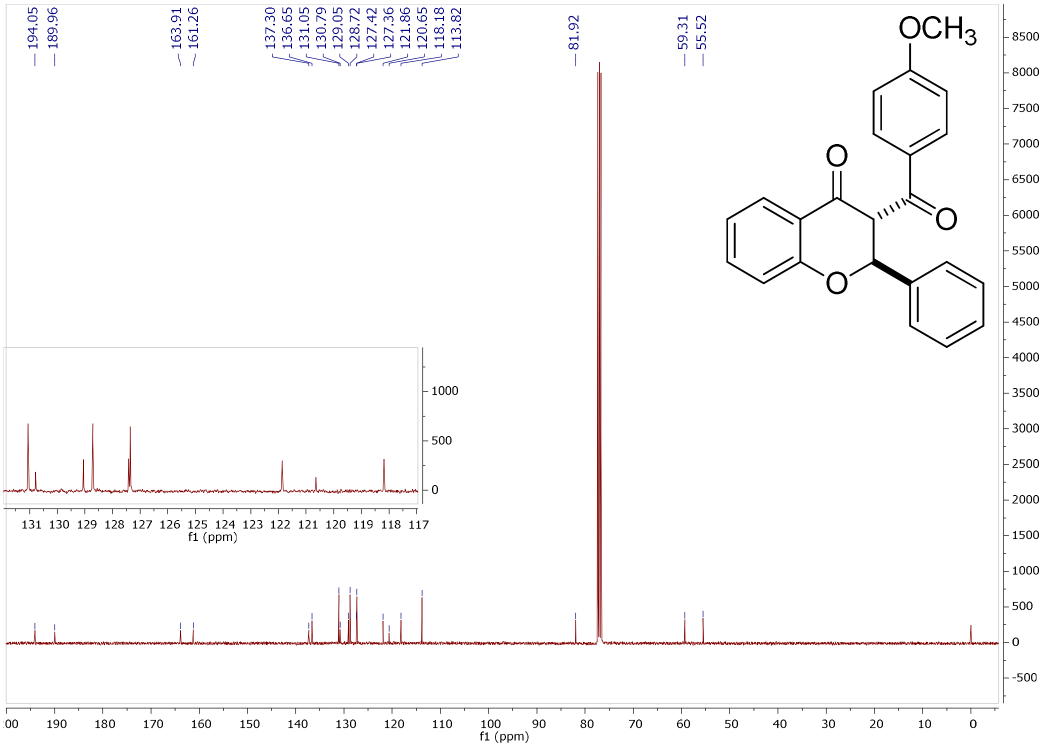

The ^{1}H NMR (400 MHz), ^{13}C NMR (100 MHz) spectra of Compound **2m** in $CDCl_3$

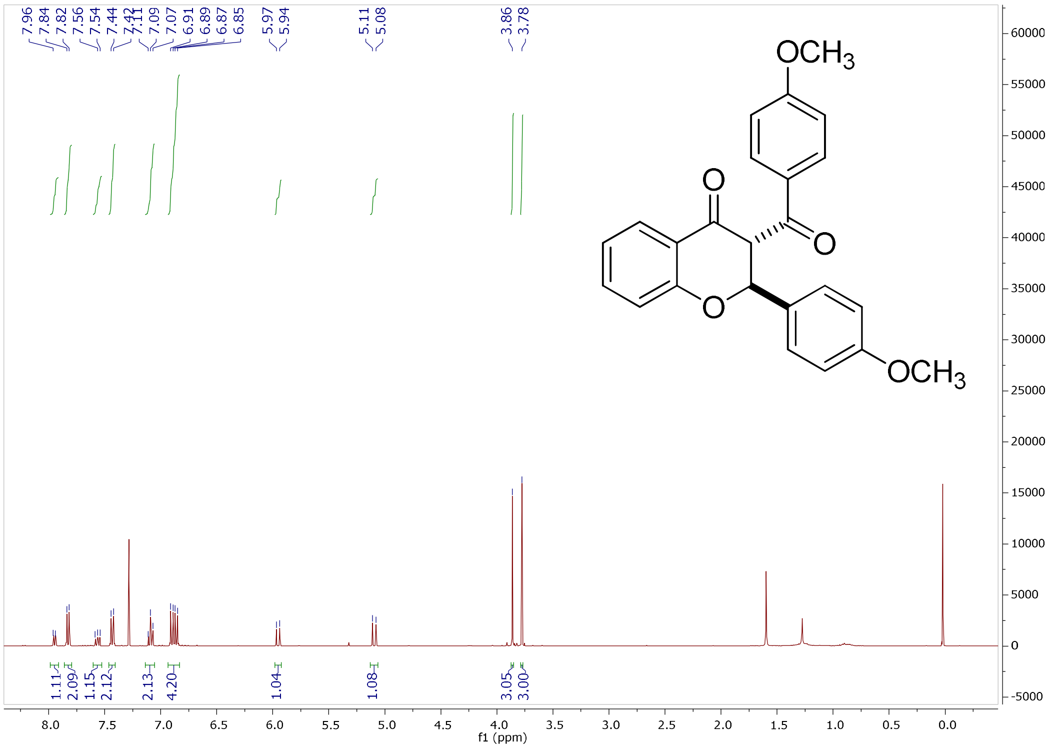

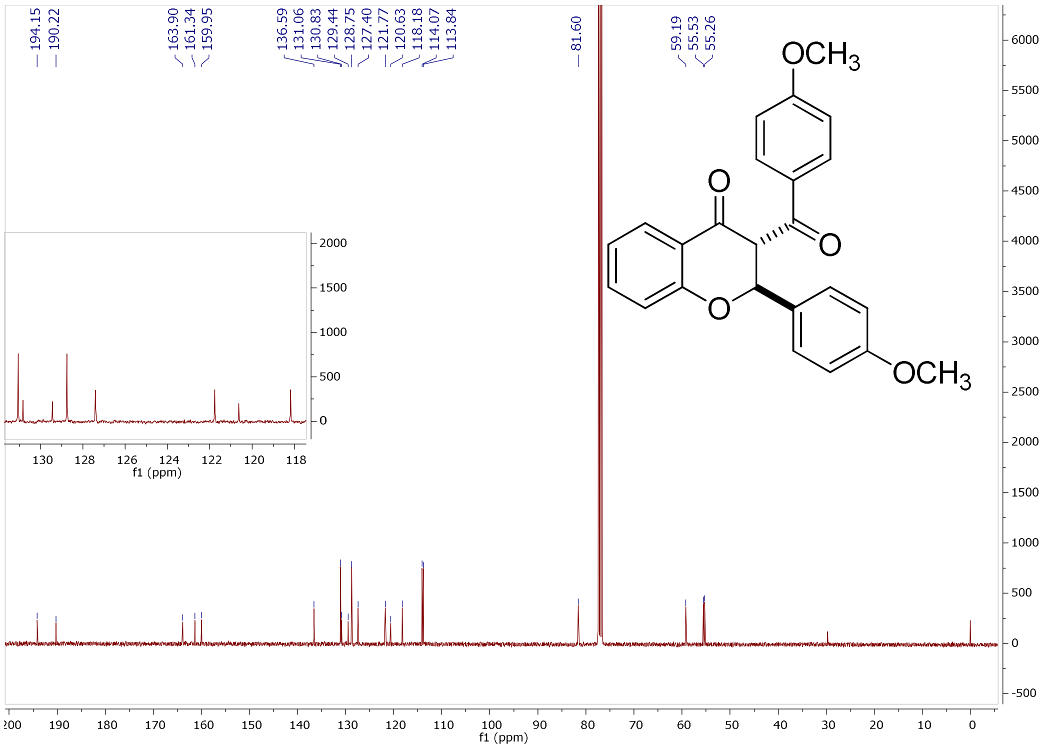

The ^{1}H NMR (400 MHz), ^{13}C NMR (100 MHz) spectra of Compound **2n** in $CDCl_3$

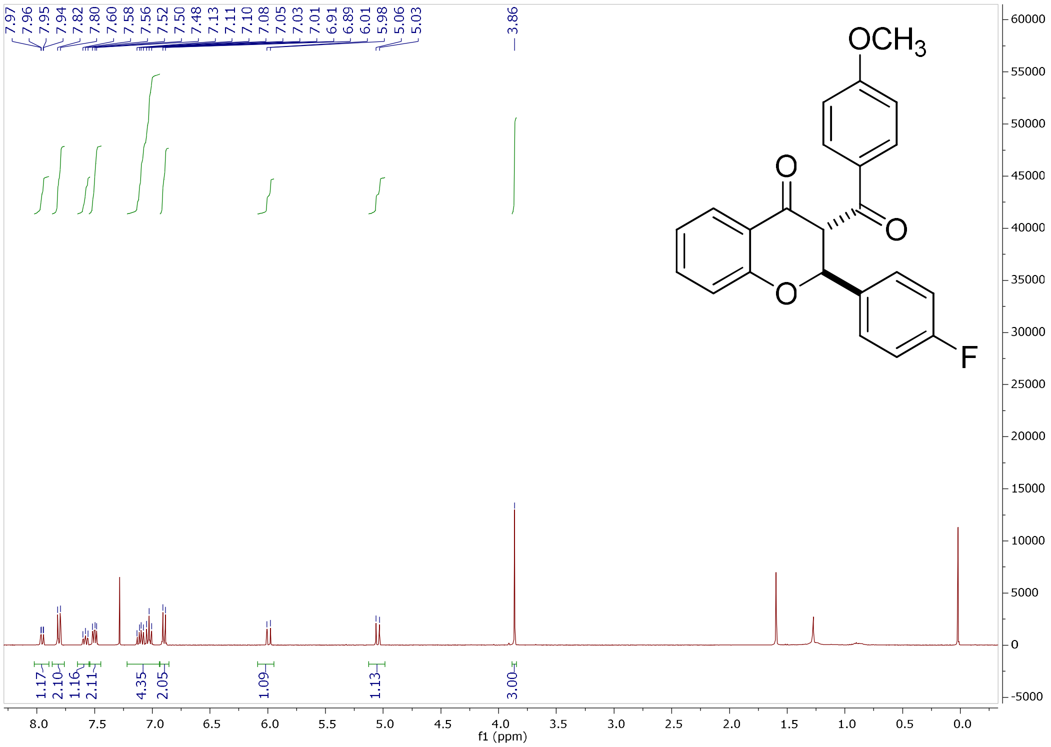

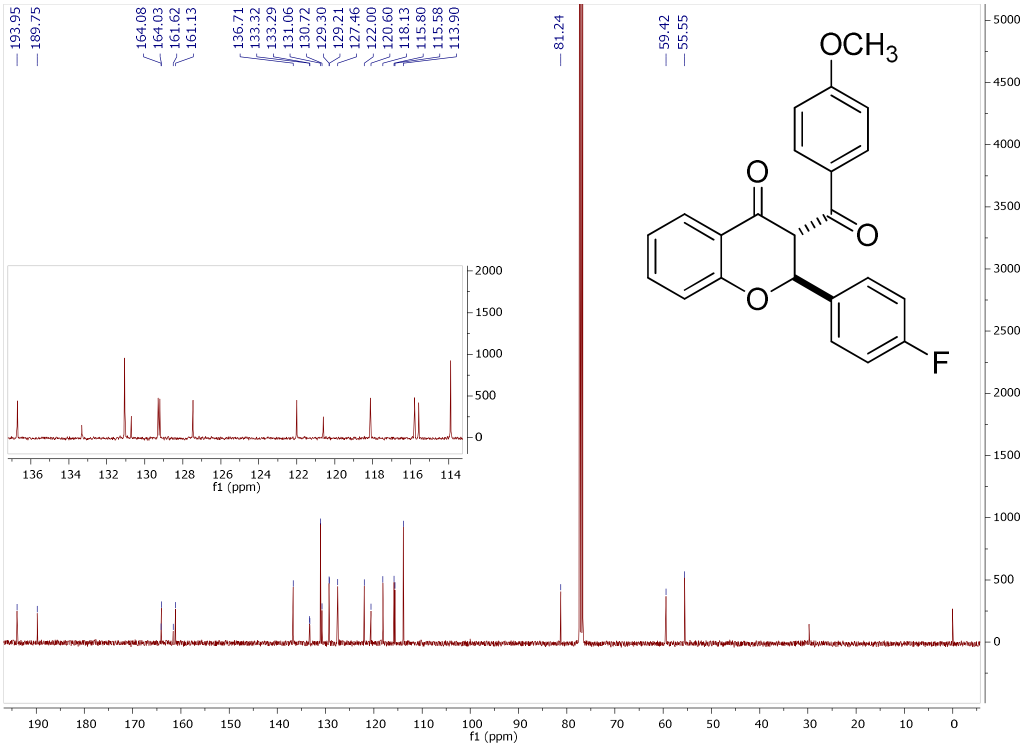

The ^{1}H NMR (400 MHz), ^{13}C NMR (100 MHz) spectra of Compound **2o** in $CDCl_3$

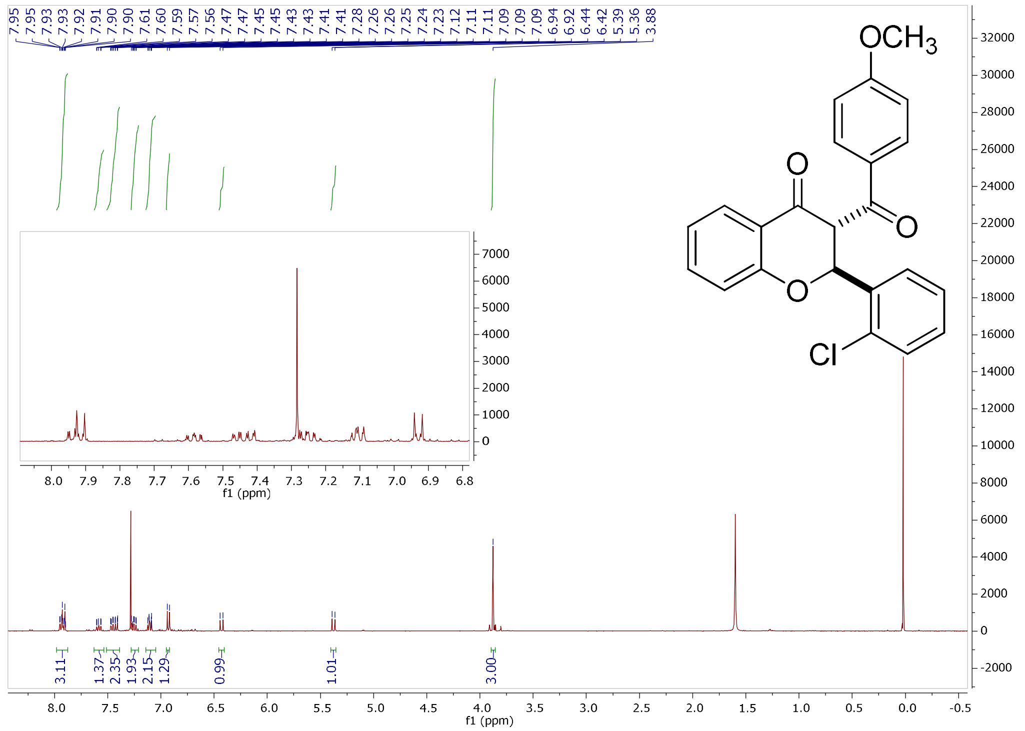

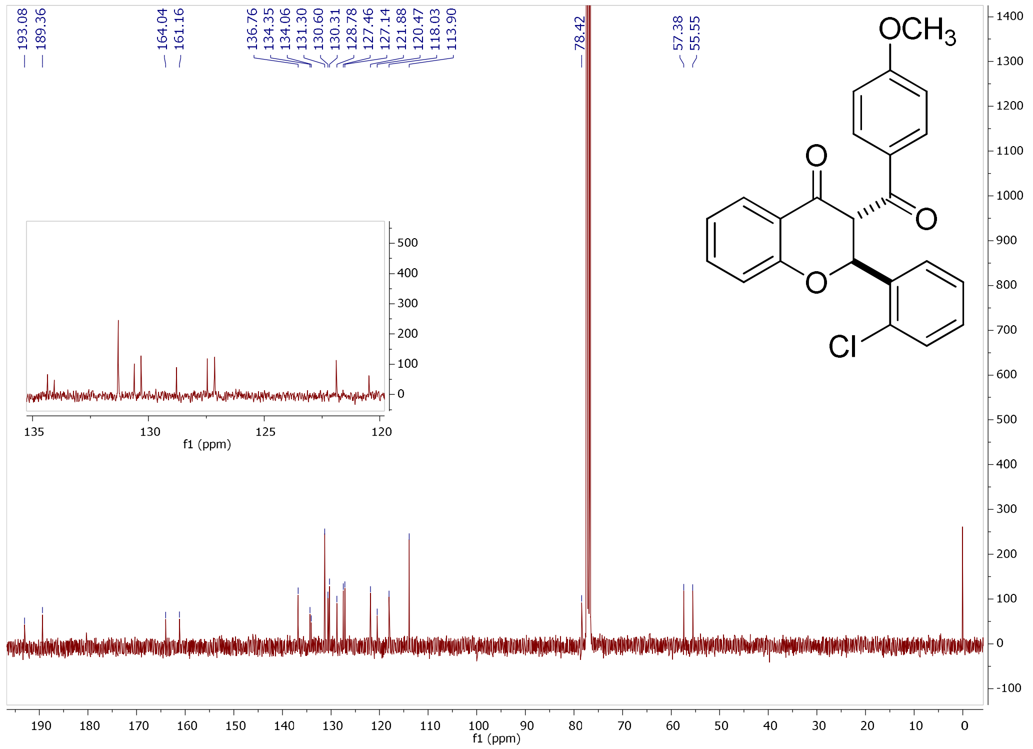

The ^{1}H NMR (400 MHz), ^{13}C NMR (100 MHz) spectra of Compound **2p** in $CDCl_3$

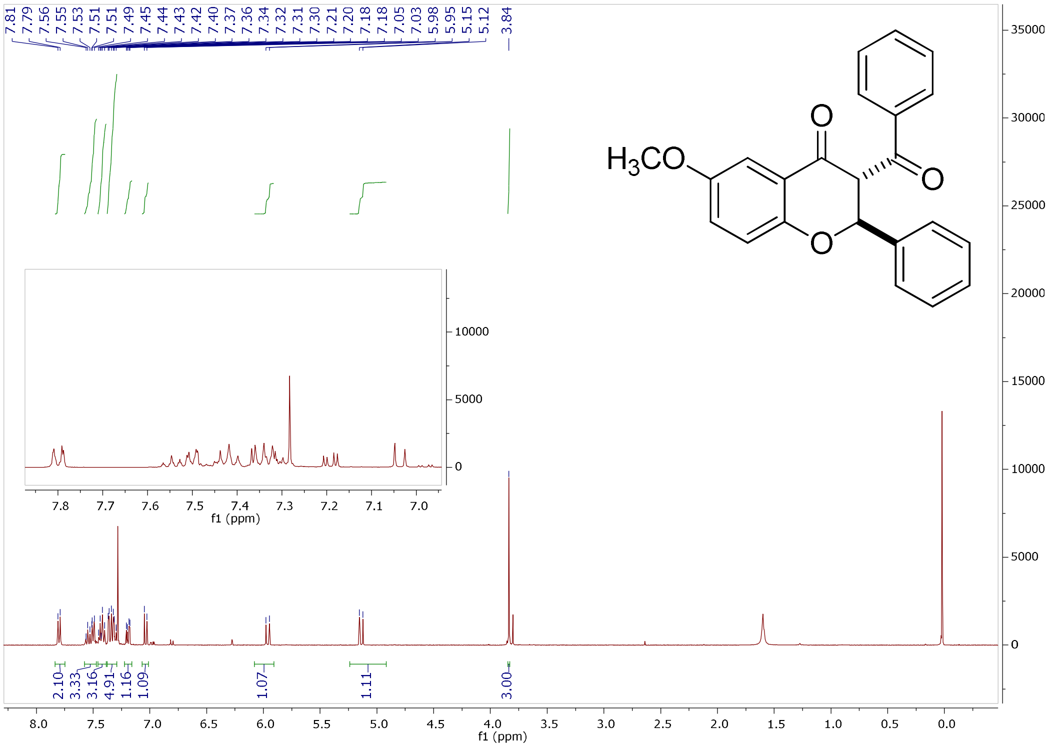

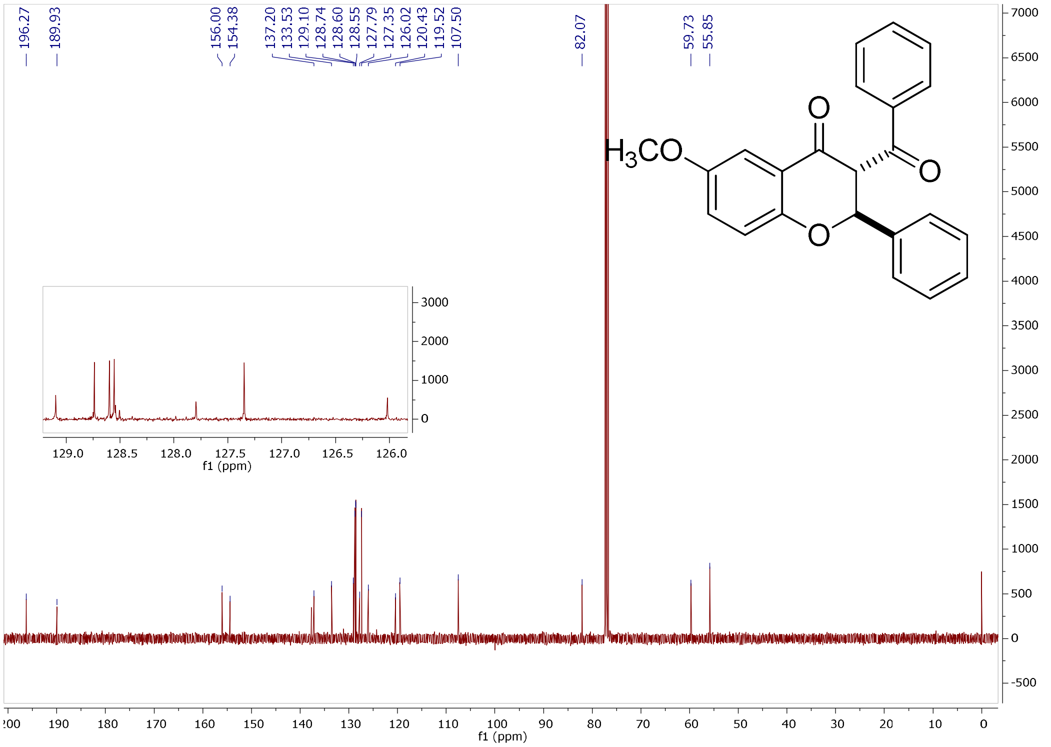

The ^{1}H NMR (400 MHz), ^{13}C NMR (100 MHz) spectra of Compound **2q** in $CDCl_3$

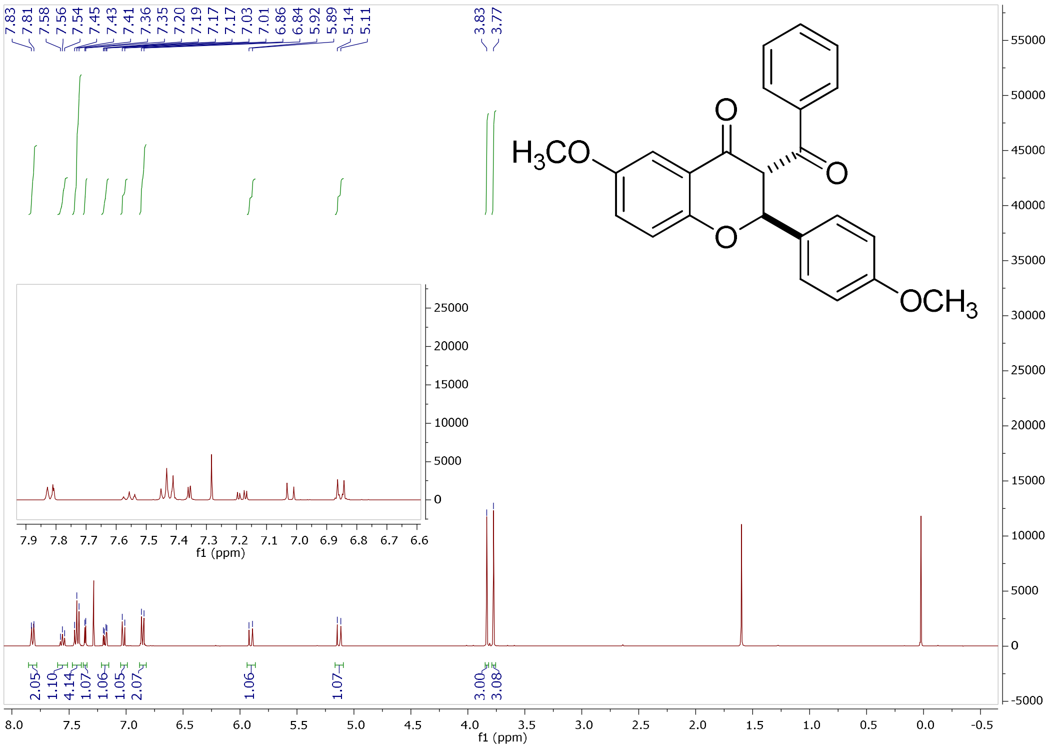

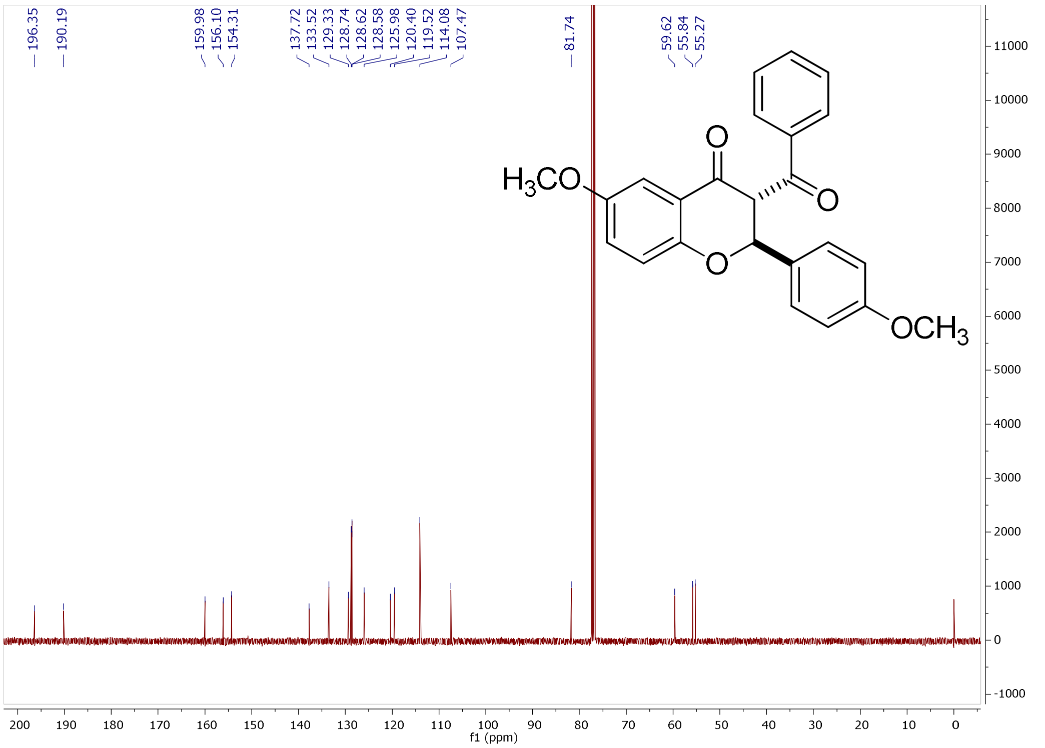

The ^{1}H NMR (400 MHz), ^{13}C NMR (100 MHz) spectra of Compound **2r** in $CDCl_3$

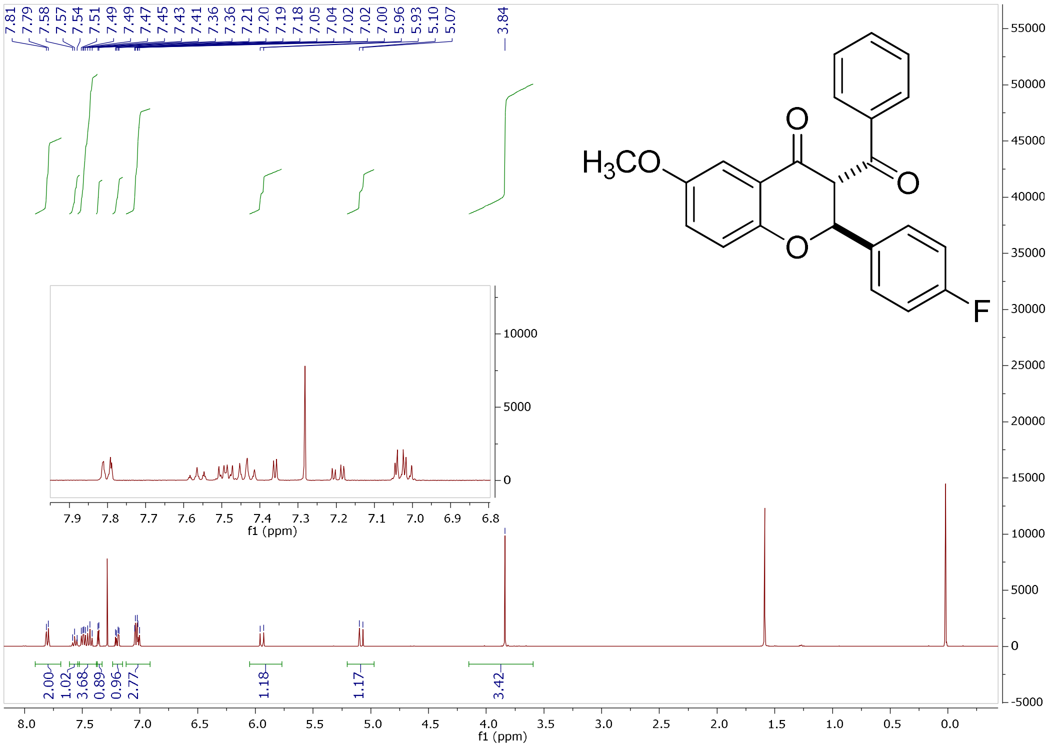

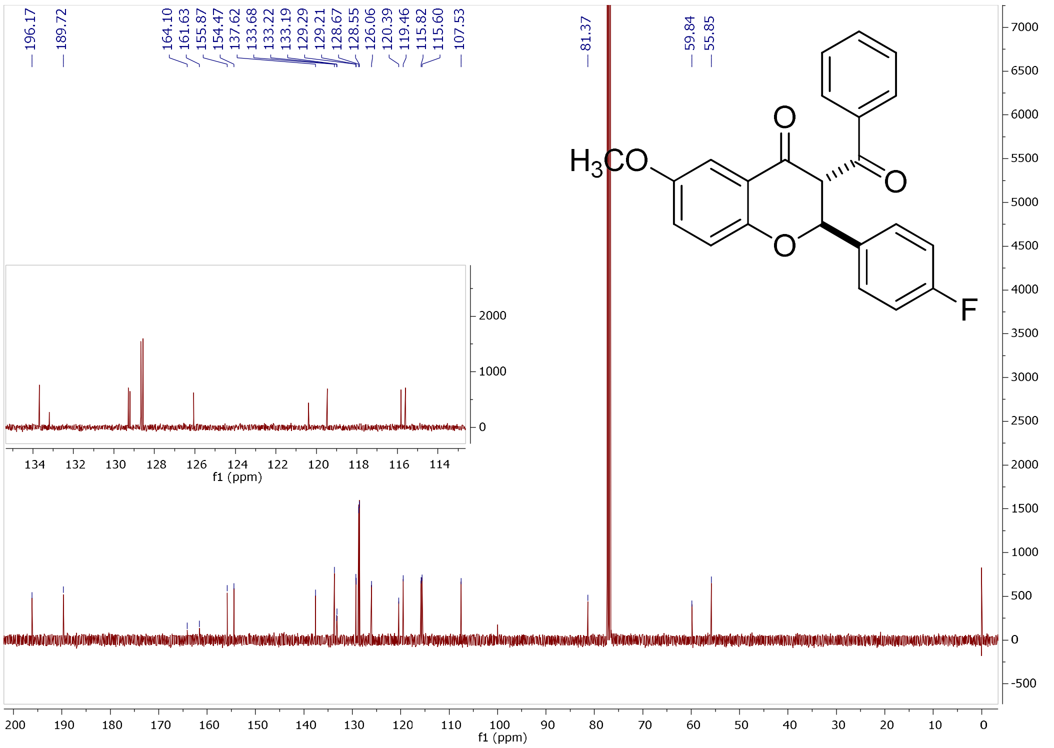

The ^{1}H NMR (400 MHz), ^{13}C NMR (100 MHz) spectra of Compound **2s** in $CDCl_3$

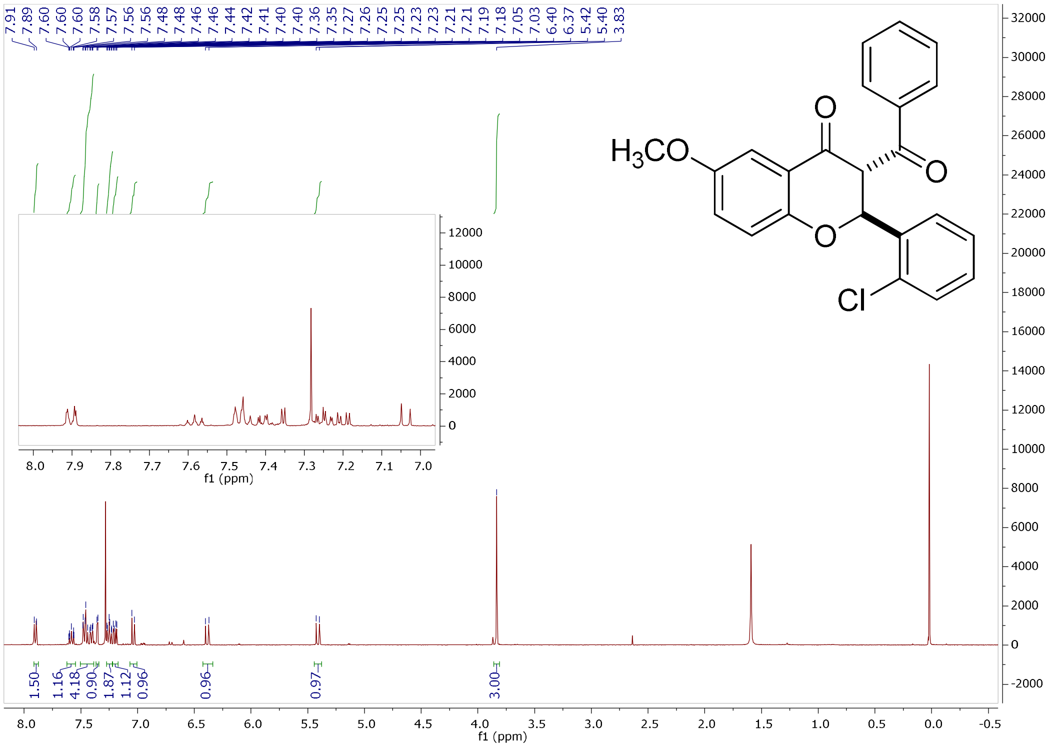

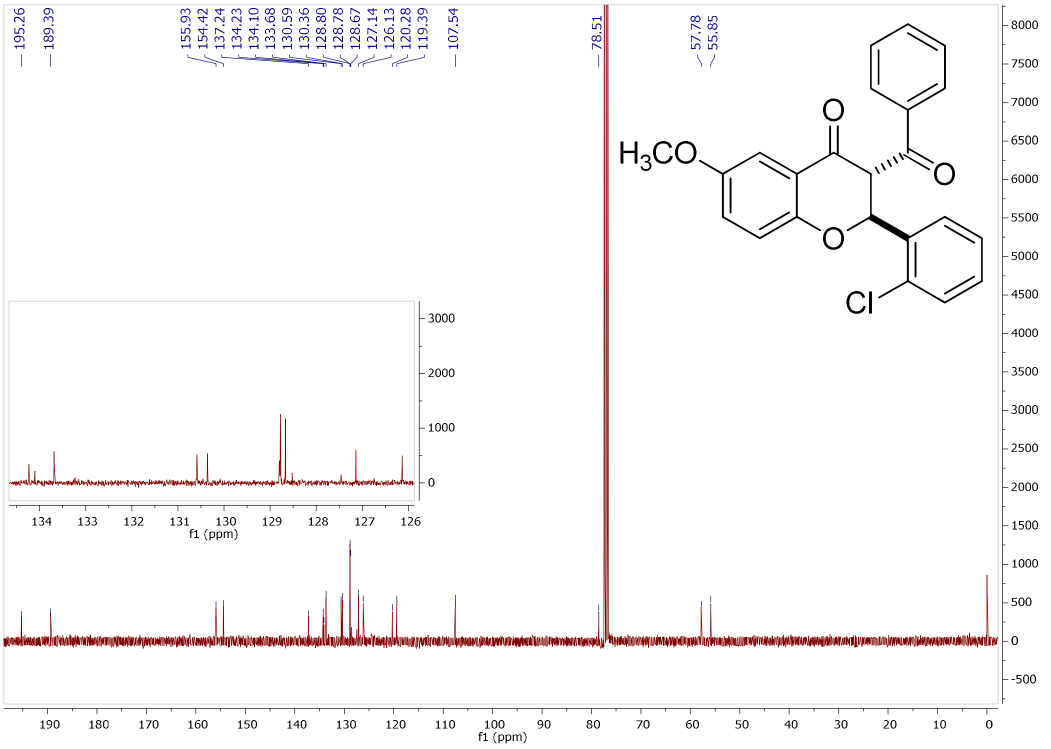

The ^{1}H NMR (400 MHz), ^{13}C NMR (100 MHz) spectra of Compound **2t** in $CDCl_3$

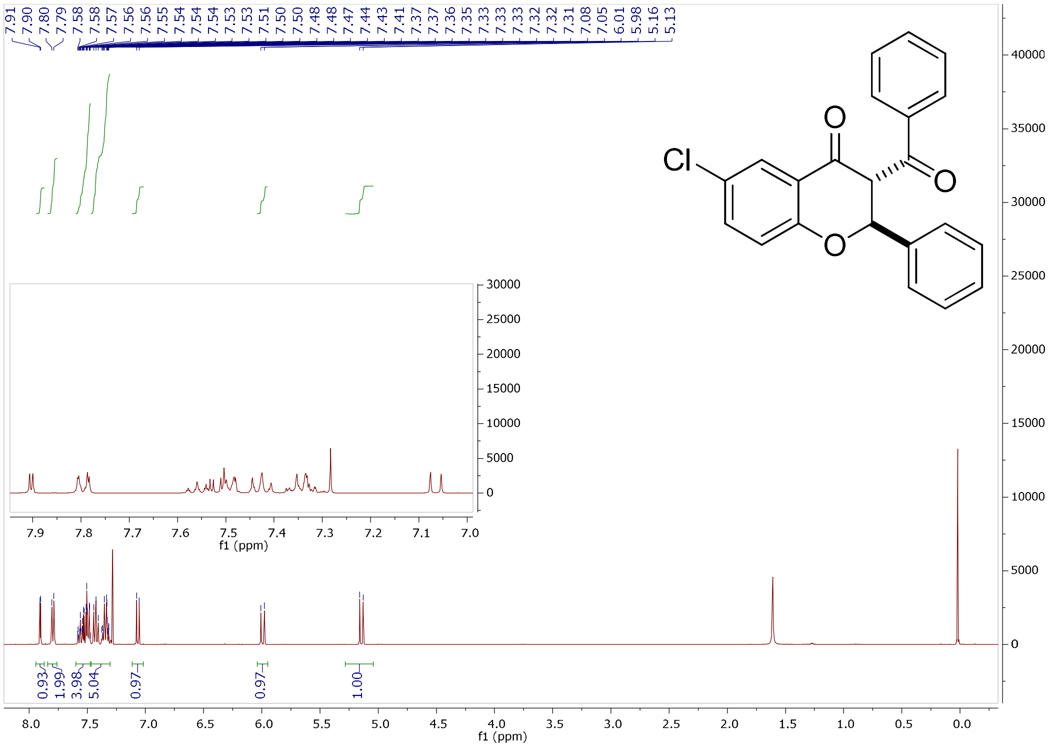

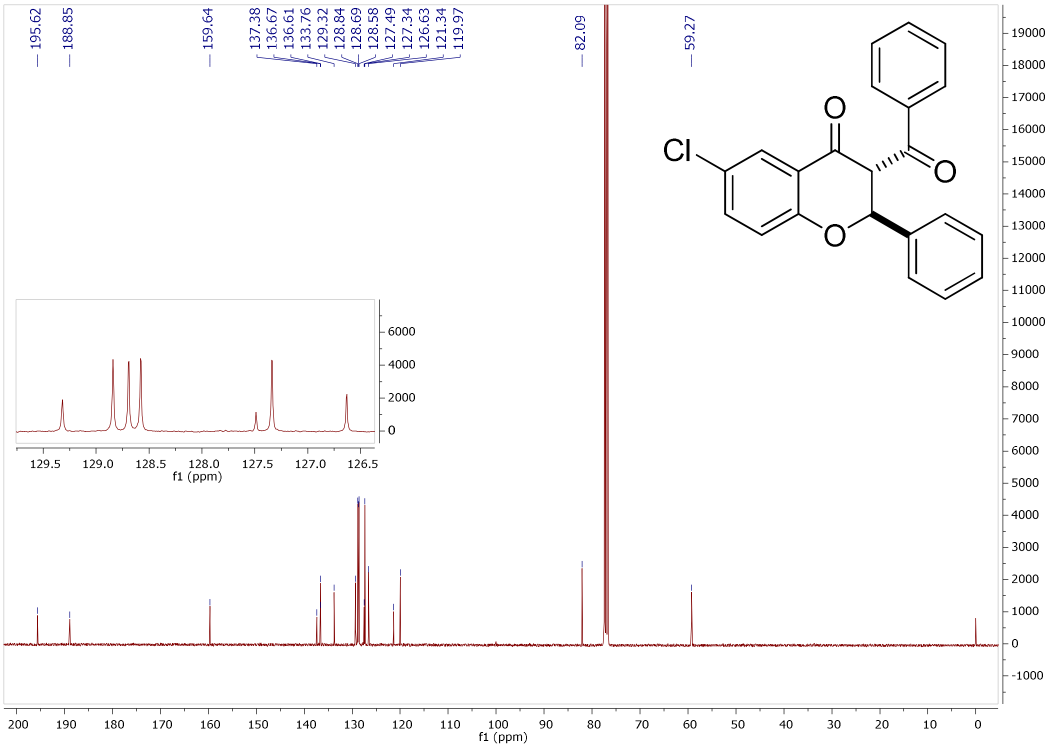

The ^{1}H NMR (400 MHz), ^{13}C NMR (100 MHz) spectra of Compound **2u** in $CDCl_3$

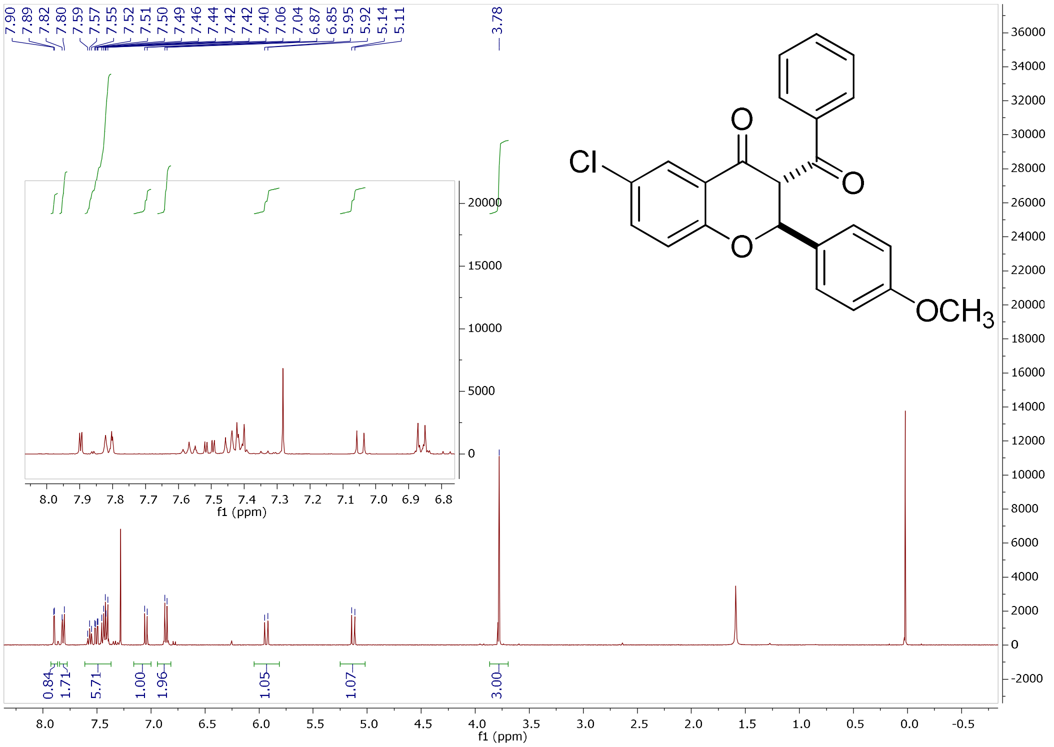

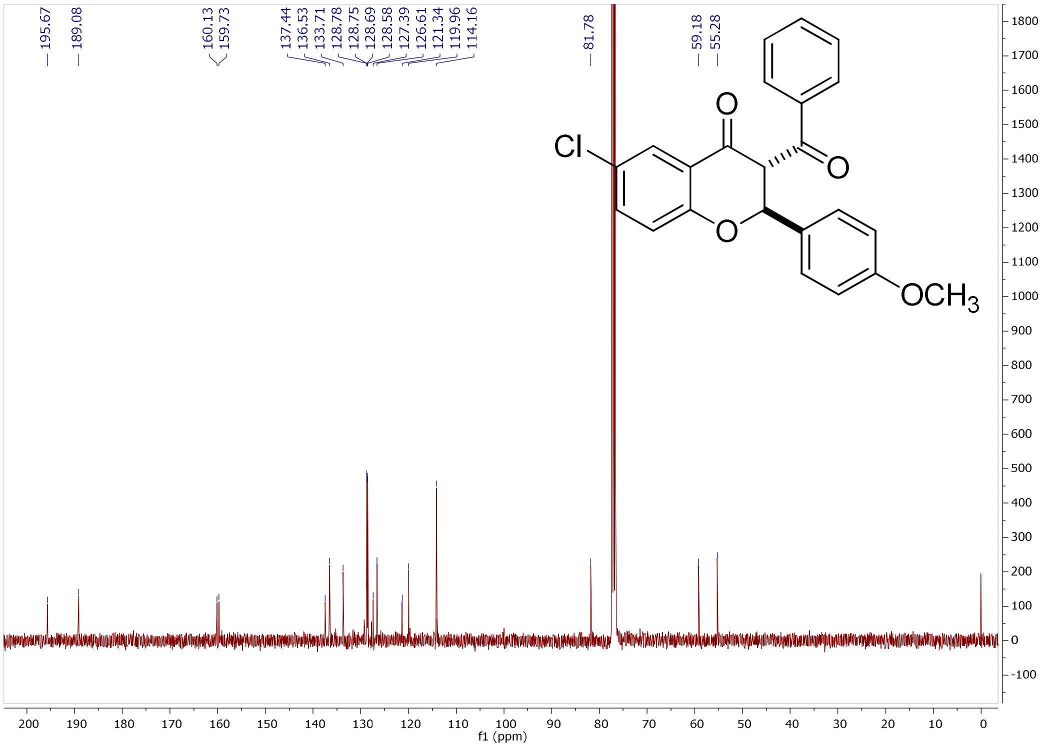

The ^{1}H NMR (400 MHz), ^{13}C NMR (100 MHz) spectra of Compound **2v** in $CDCl_3$

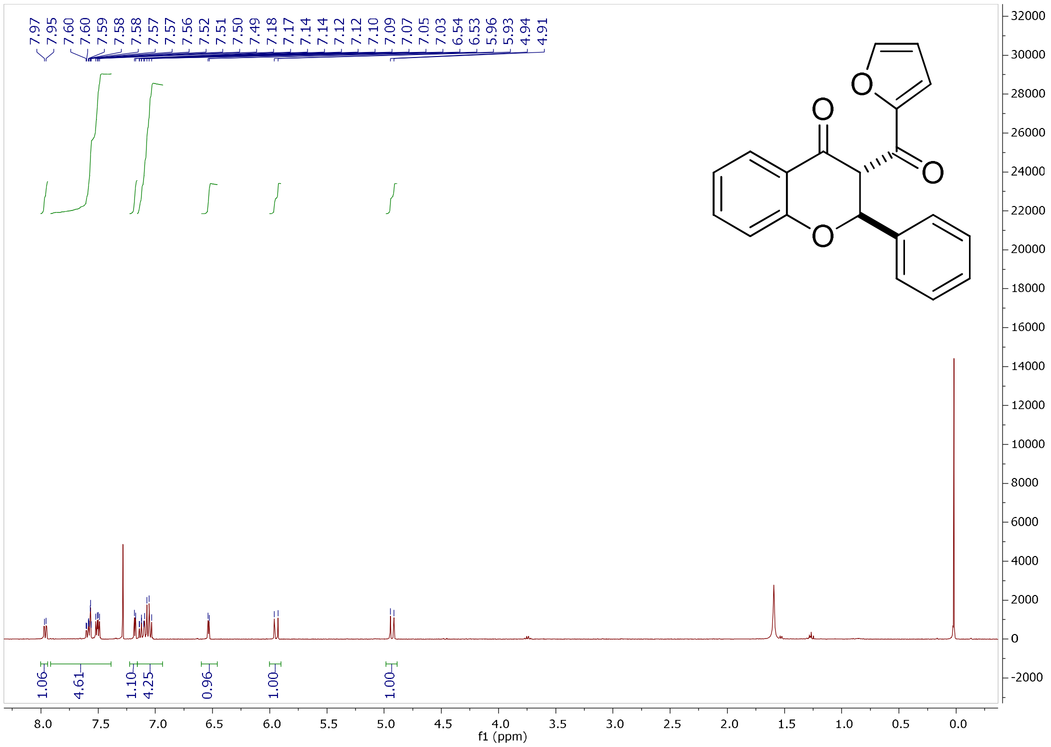

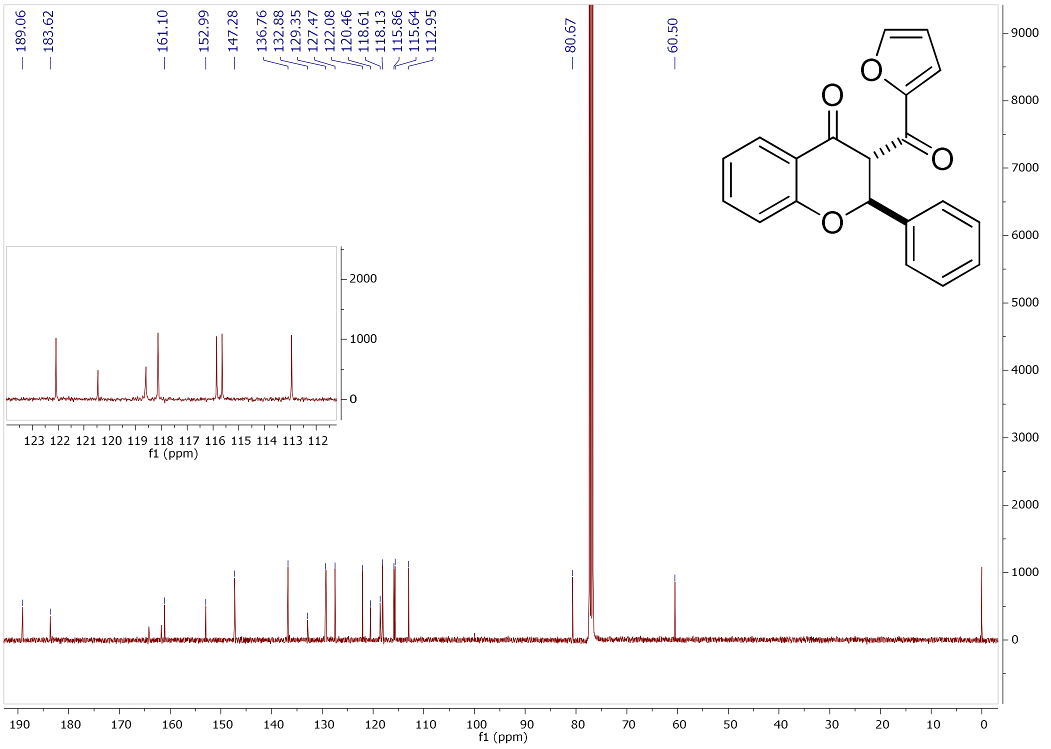

The ^{1}H NMR (400 MHz), ^{13}C NMR (100 MHz) spectra of Compound **2w** in $CDCl_3$

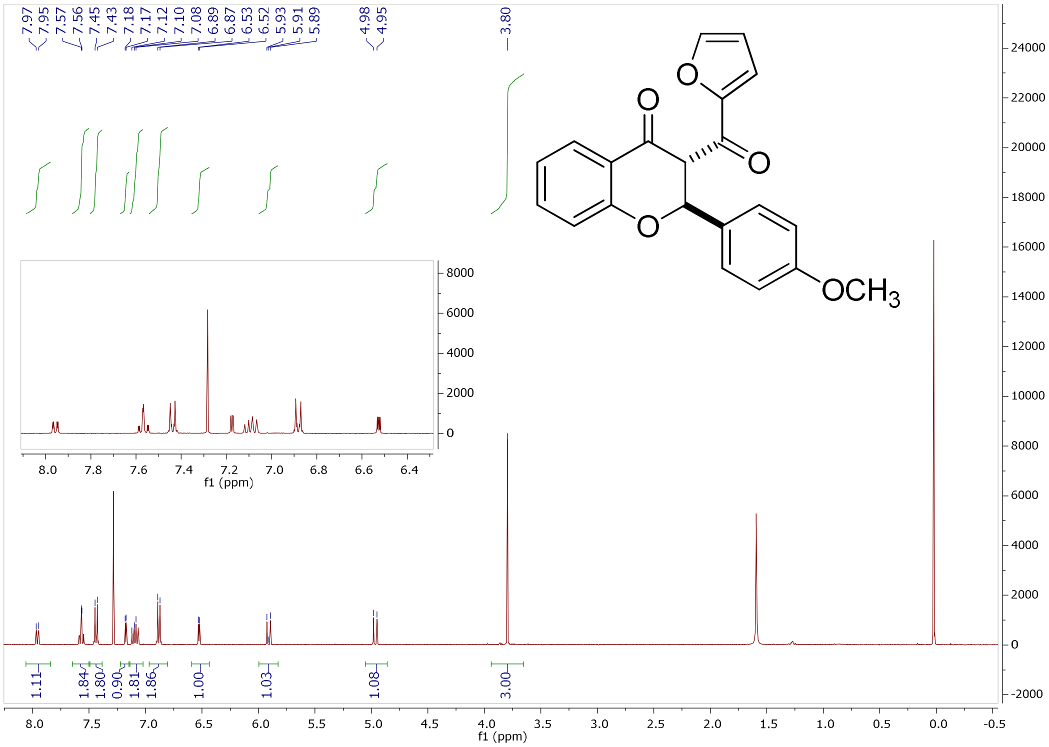

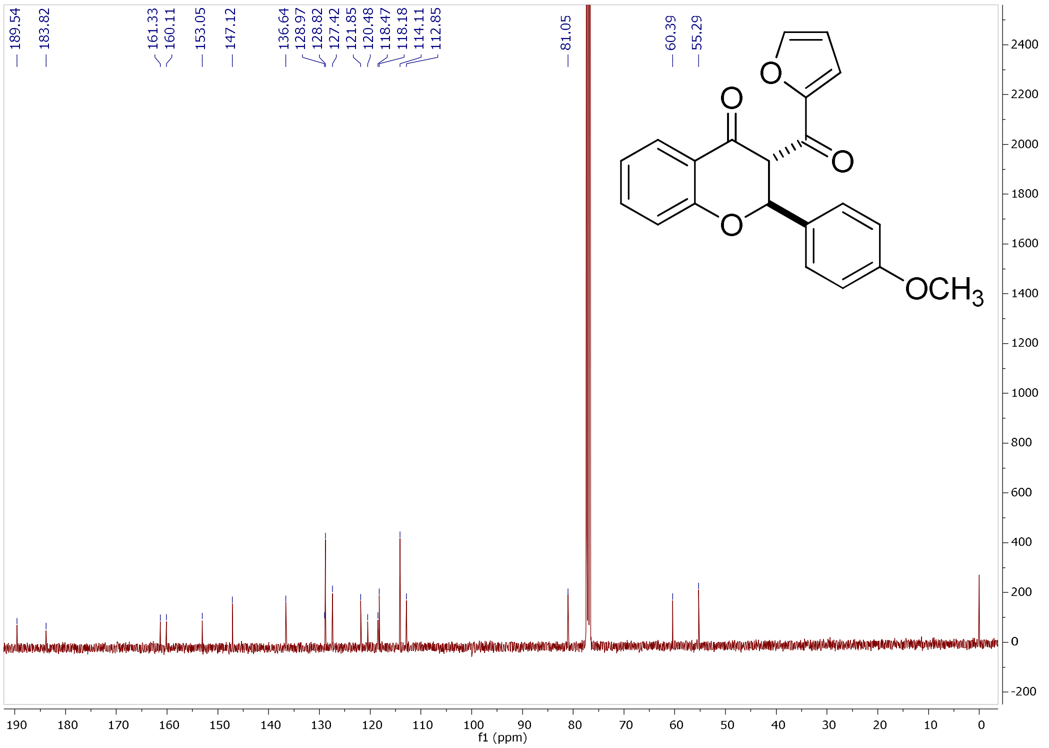

The ^{1}H NMR (400 MHz), ^{13}C NMR (100 MHz) spectra of Compound **2x** in $CDCl_3$

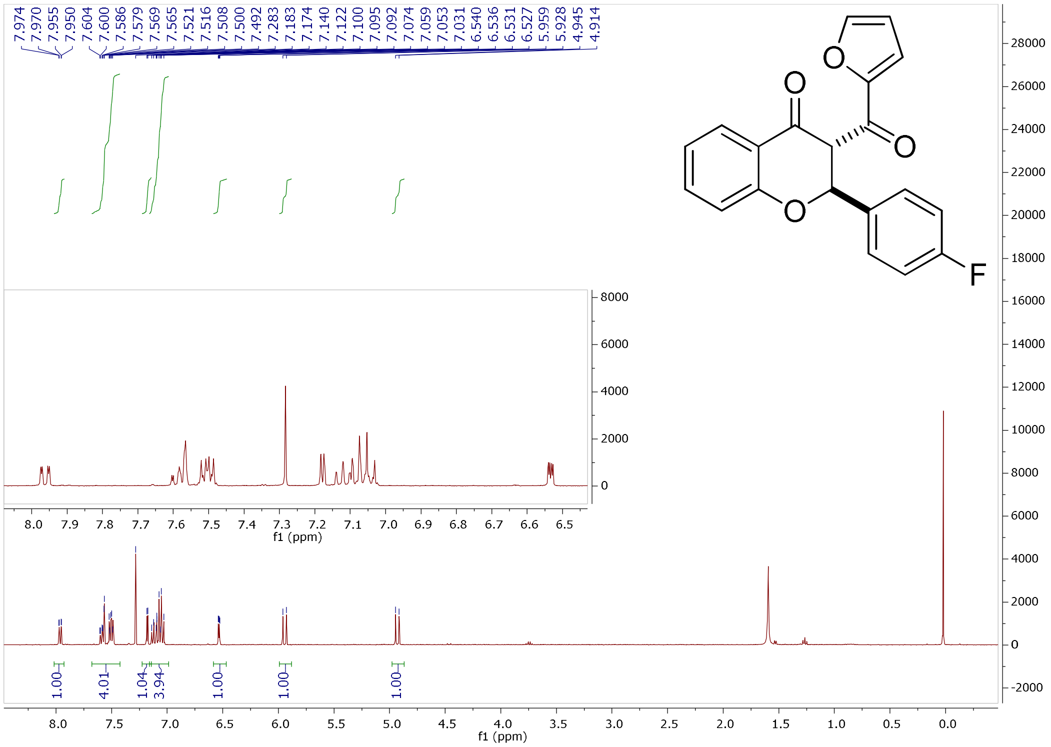

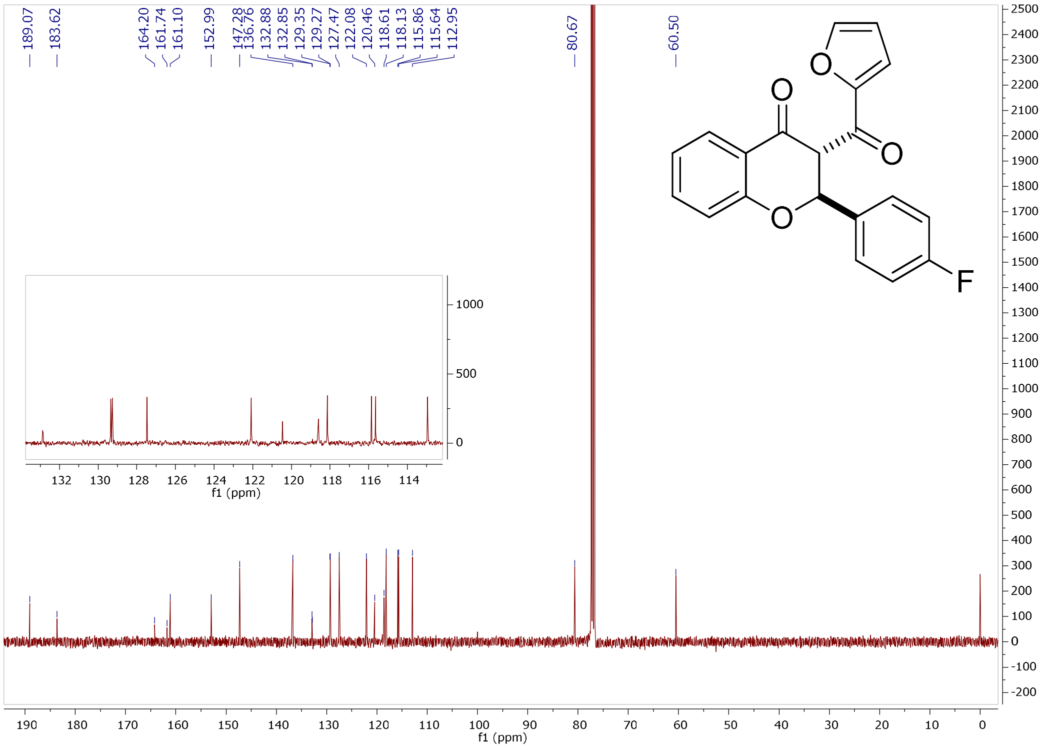

The ^{1}H NMR (400 MHz), ^{13}C NMR (100 MHz) spectra of Compound **2y** in $CDCl_3$

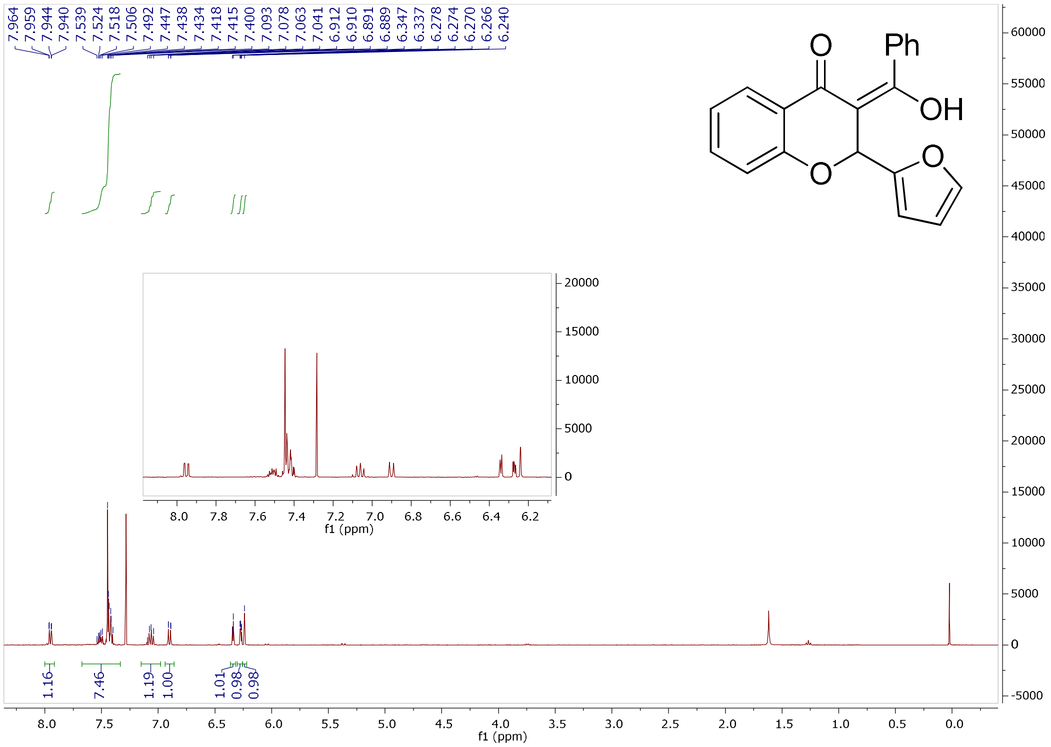

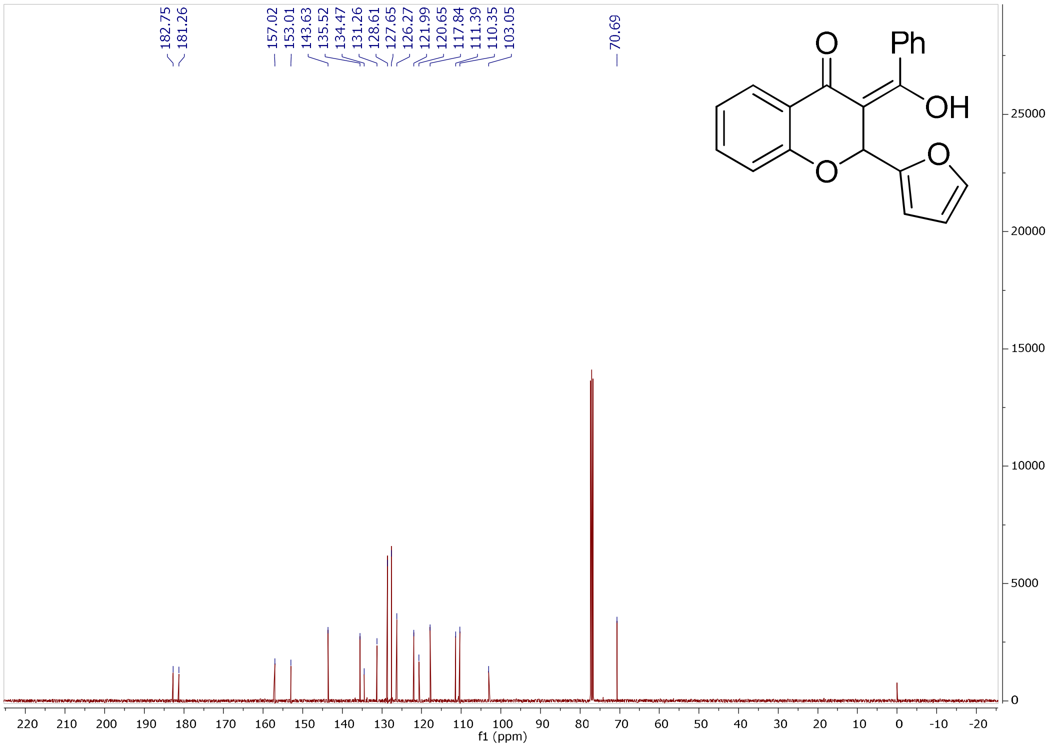

The ^{1}H NMR (400 MHz), ^{13}C NMR (100 MHz) spectra of Compound **2z** in $CDCl_3$

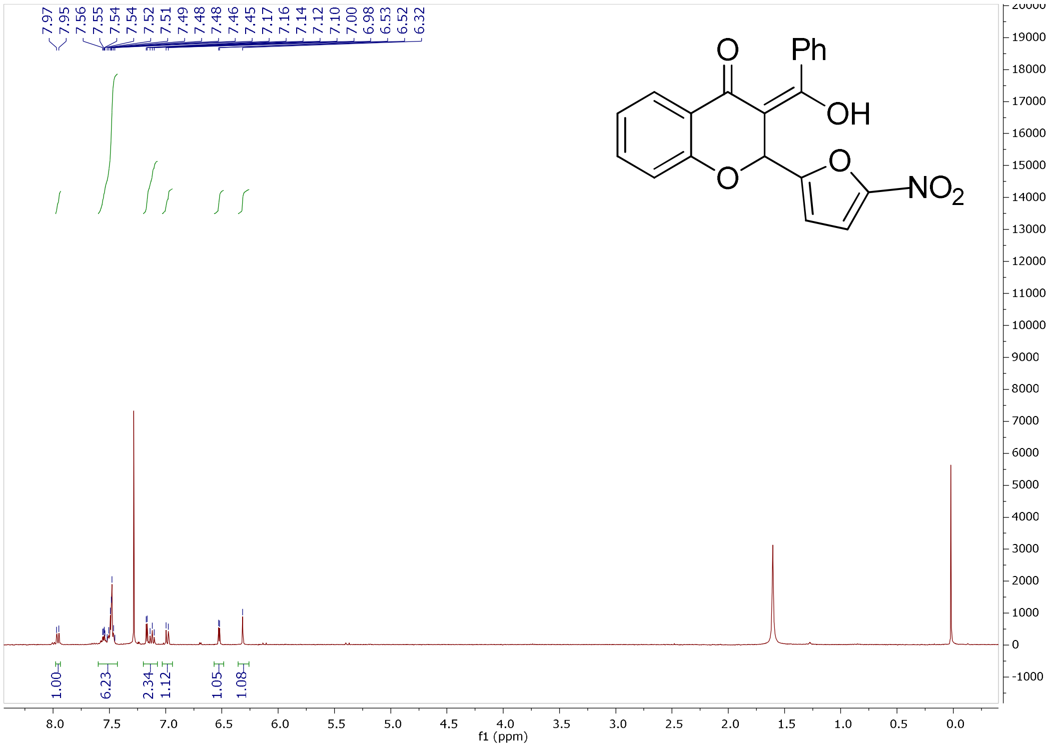

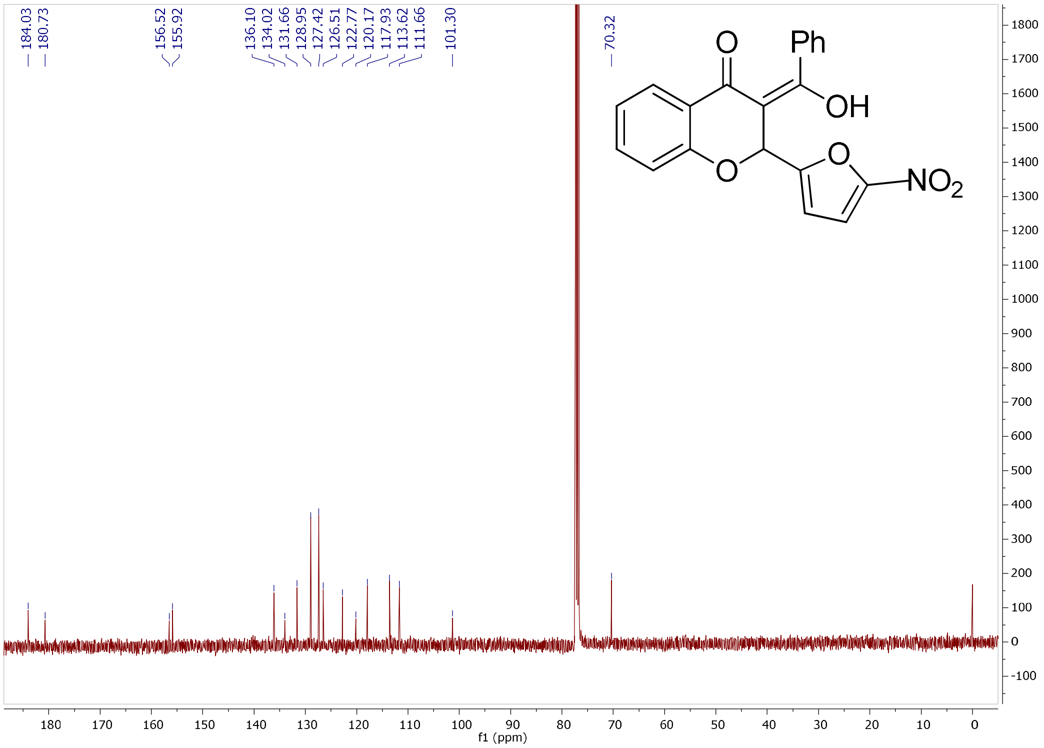

The ^{1}H NMR (400 MHz), ^{13}C NMR (100 MHz) spectra of Compound **3** in $CDCl_3$

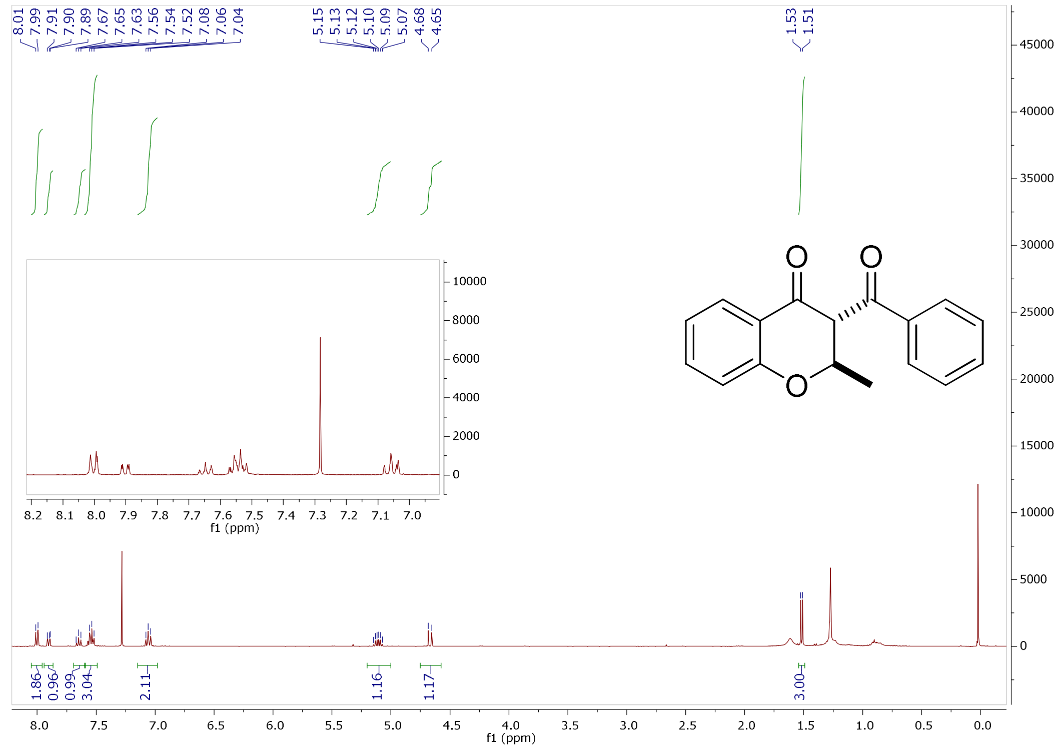

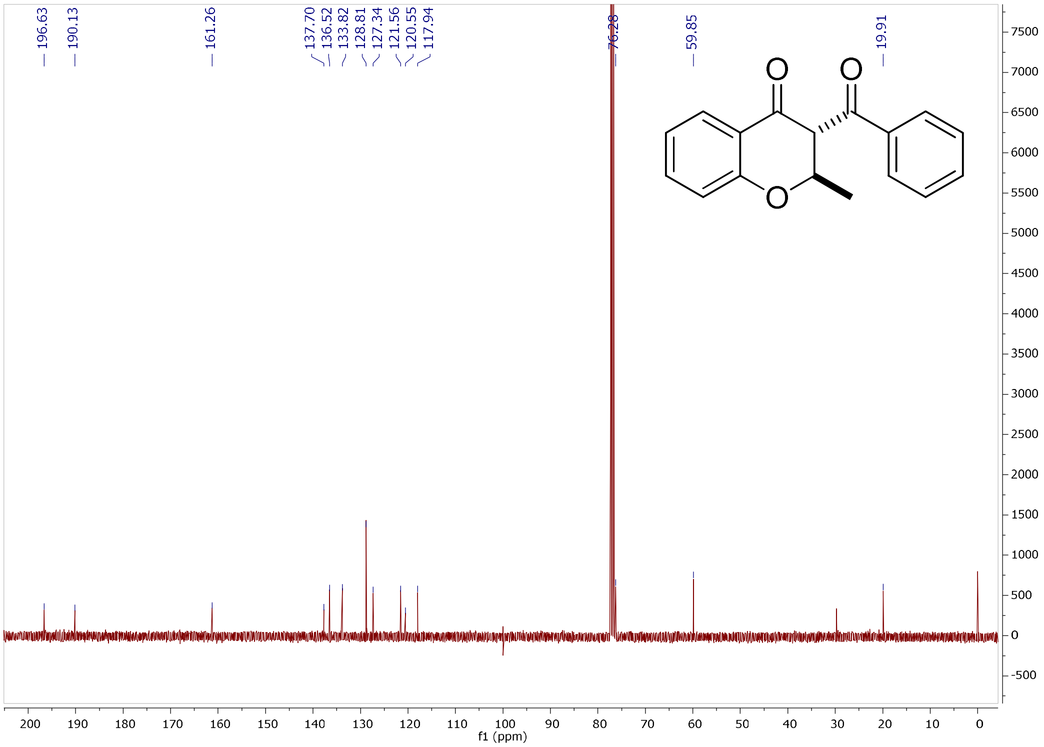

The ^{1}H NMR (400 MHz), ^{13}C NMR (100 MHz) spectra of Compound **4** in $CDCl_3$

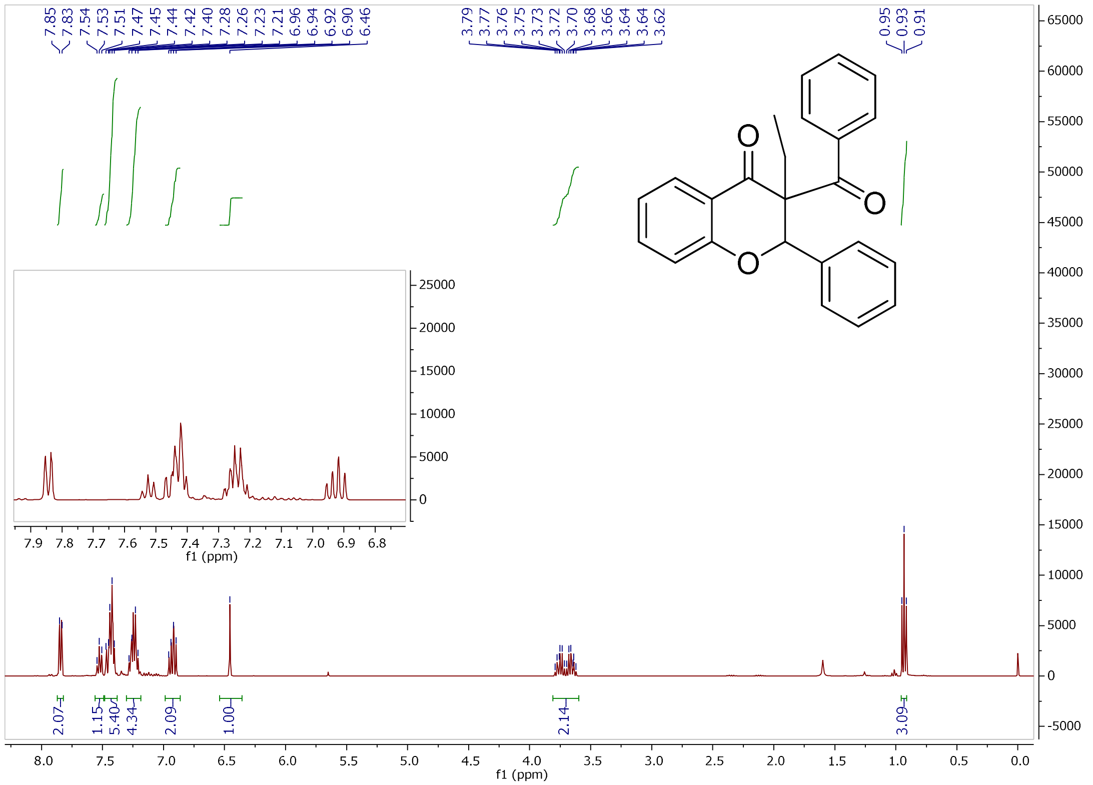

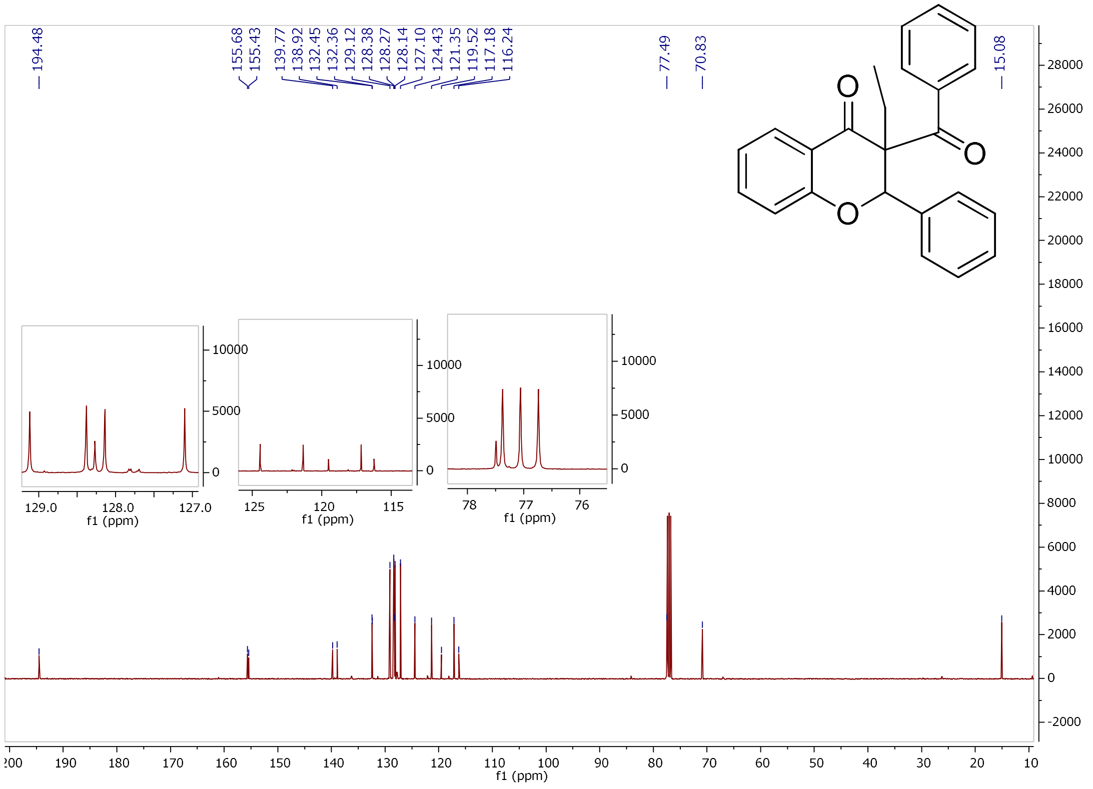

The ^{1}H NMR (400 MHz), ^{13}C NMR (100 MHz) spectra of Compound **5** in $CDCl_3$

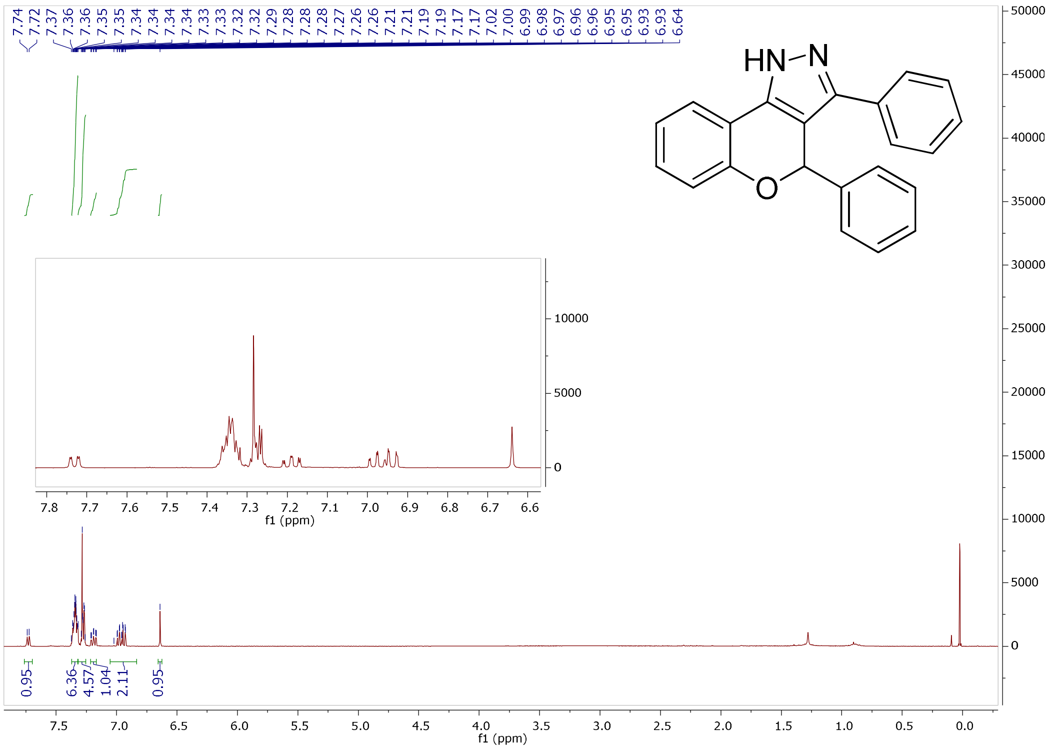

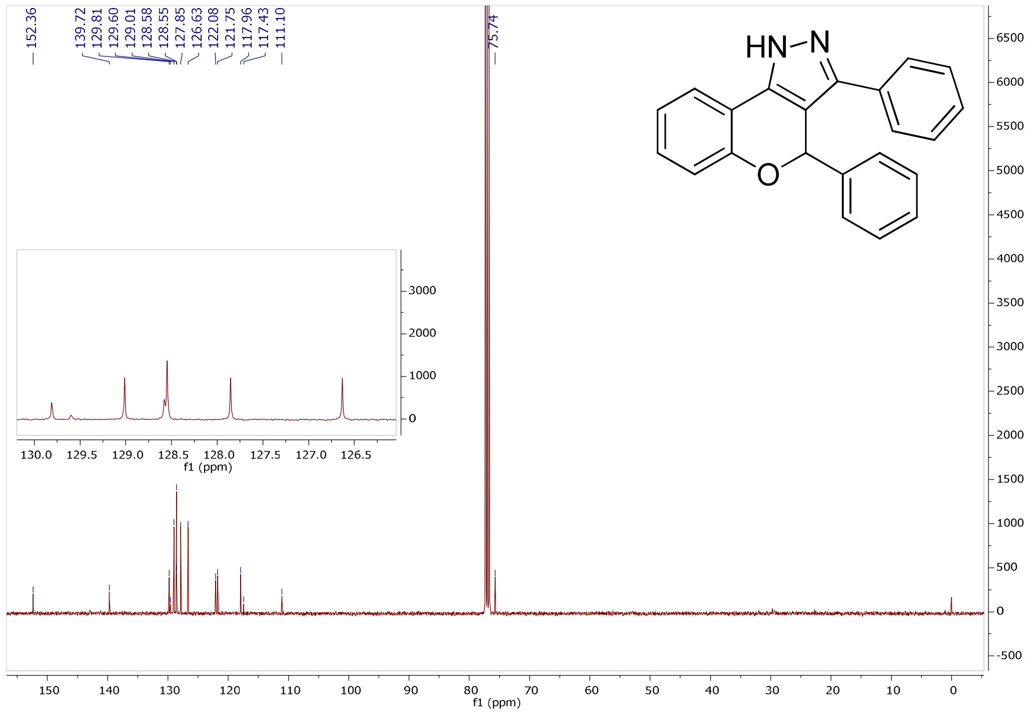

Supplement: Supplementary file 1 [file molecules-25-00397-s001.pdf]
